# Ammonia Oxidation and Nitrite Reduction in the Verrucomicrobial Methanotroph *Methylacidiphilum fumariolicum* SolV

**DOI:** 10.3389/fmicb.2017.01901

**Published:** 2017-09-27

**Authors:** Sepehr S. Mohammadi, Arjan Pol, Theo van Alen, Mike S. M. Jetten, Huub J. M. Op den Camp

**Affiliations:** Department of Microbiology, Faculty of Science, Institute for Water and Wetland Research, Radboud University, Nijmegen, Netherlands

**Keywords:** Methylacidiphilum, methanotroph, ammonia, methane, nitrite, reactive N compounds

## Abstract

The Solfatara volcano near Naples (Italy), the origin of the recently discovered verrucomicrobial methanotroph *Methylacidiphilum fumariolicum* SolV was shown to contain ammonium (${\text{NH}}_{4}^{+}$) at concentrations ranging from 1 to 28 mM. Ammonia (NH_3_) can be converted to toxic hydroxylamine (NH_2_OH) by the particulate methane monooxygenase (pMMO), the first enzyme of the methane (CH_4_) oxidation pathway. Methanotrophs rapidly detoxify the intermediate NH_2_OH. Here, we show that strain SolV performs ammonium oxidation to nitrite at a rate of 48.2 nmol ${\text{NO}}_{2}^{-}$.h^−1^.mg DW^−1^ under O_2_ limitation in a continuous culture grown on hydrogen (H_2_) as an electron donor. In addition, strain SolV carries out nitrite reduction at a rate of 74.4 nmol ${\text{NO}}_{2}^{-}$.h^−1^.mg DW^−1^ under anoxic condition at pH 5–6. This range of pH was selected to minimize the chemical conversion of nitrite (${\text{NO}}_{2}^{-}$) potentially occurring at more acidic pH values. Furthermore, at pH 6, we showed that the affinity constants (K_*s*_) of the cells for NH_3_ vary from 5 to 270 μM in the batch incubations with 0.5–8% (v/v) CH_4_, respectively. Detailed kinetic analysis showed competitive substrate inhibition between CH_4_ and NH_3_. Using transcriptome analysis, we showed up-regulation of the gene encoding hydroxylamine dehydrogenase (*haoA*) cells grown on H_2_/${\text{NH}}_{4}^{+}$ compared to the cells grown on CH_4_/${\text{NO}}_{3}^{-}$ which do not have to cope with reactive N-compounds. The denitrifying genes *nirk* and *norC* showed high expression in H_2_/${\text{NH}}_{4}^{+}$ and CH_4_/${\text{NO}}_{3}^{-}$ grown cells compared to cells growing at μ_max_ (with no limitation) while the *norB* gene showed downregulation in CH_4_/${\text{NO}}_{3}^{-}$ grown cells. These cells showed a strong upregulation of the genes in nitrate/nitrite assimilation. Our results demonstrate that strain SolV can perform ammonium oxidation producing nitrite. At high concentrations of ammonium this may results in toxic effects. However, at low oxygen concentrations strain SolV is able to reduce nitrite to N_2_O to cope with this toxicity.

## Introduction

Methane (CH_4_) is a powerful greenhouse gas, which is released in to the atmosphere both from natural and anthropogenic sources (Conrad, 2009). Understanding sources and sinks of CH_4_ is important for future models of climate change on our planet. Methane oxidizing microorganisms are one of the most important biological sinks of CH_4_ (Murrell and Jetten, 2009).

Aerobic methanotrophic bacteria belong to a physiological group of bacteria recognized as methylotrophs. The proteobacterial methanotrophs are distinctive in their ability to exploit CH_4_ as the only carbon and energy source (Hanson and Hanson, 1996). Recently, three independent research groups discovered extreme acidophilic methanotrophic *Verrucomicrobia* in geothermal regions (Dunfield et al., 2007; Pol et al., 2007; Islam et al., 2008). Prior to this finding, obligate aerobic methanotrophs were speculated to be exclusively represented in the *Alpha* and *Gamma* subclasses of the *Proteobacteria*. Analysis of the 16S ribosomal RNA and *pmoA* genes demonstrated that the new *Verrucomicrobia* species do not form a monophyletic group with this subclasses (Heyer et al., 2005), and the new genus name *Methylacidiphilum* was suggested (Op den Camp et al., 2009). Furthermore, it has been shown that growth of the new acidophilic methanotrophic bacterium *Methylacidiphilum fumariolicum* SolV is strictly dependent on the presence of lanthanides acting as a cofactor of the methanol dehydrogenase (Keltjens et al., 2014; Pol et al., 2014). Recently, new species of mesophilic acidophilic verrucomicrobial methanotrophs were isolated and characterized from a volcanic region in Italy and the new genus *Methylacidimicrobium* was proposed (Sharp et al., 2014; van Teeseling et al., 2014). This finding expands the diversity of verrucomicrobial methanotrophs and demonstrates that they could be present in more ecosystems than formerly supposed (Chistoserdova et al., 2009). The new verrucomicrobial strains from both genera were shown to be autotrophs that use CH_4_ as the sole energy source and fix CO_2_ using the Calvin-Benson-Bassham Cycle (Khadem et al., 2011; Sharp et al., 2012, 2013, 2014; van Teeseling et al., 2014), and strain SolV was shown to be able to fix N_2_ (Khadem et al., 2010).

Methanotrophic and nitrifying microorganisms share many similarities. They grow obligately on the specific substrates, CH_4_ for methanotrophs and NH_3_ for nitrifiers. These molecules are structurally comparable and both are highly reduced. Many of these types of microorganisms have intracellular membrane structures where the membrane bound ammonia monooxygenase (AMO) or CH_4_ monooxygenase (pMMO) are localized. In the first step of aerobic CH_4_ or NH_3_ oxidation, the monooxygenase enzymes introduce a single oxygen atom from O_2_ into CH_4_ or NH_3_, producing methanol from CH_4_ and hydroxylamine from NH_3_ (Stein et al., 2012). Both microorganisms are able to co-oxidize a range of other substrates and are inhibited by similar compounds (Bédard and Knowles, 1989; Stein et al., 2012). Nitrifiers are able to oxidize CH_4_, and methanotrophs are capable of nitrification. It has been shown that in nutrient limited situations, methanotrophs do participate in soil nitrification, mainly in the production of N_2_O. Nitrification by aerobic methanotrophs relies on CH_4_, because they cannot grow on NH_3_ (Stein et al., 2012). Recent studies of CH_4_ oxidation and N_2_O production in soils using stable isotopes and particular inhibitors offered more evidence for a role of methanotrophic bacteria in nitrification (Mandernack et al., 2000; Lee et al., 2009; Acton and Baggs, 2011; Im et al., 2011).

${\text{NH}}_{4}^{+}$ is a nitrogen source for methanotrophic bacteria but was also shown to inhibit CH_4_ oxidation in the model organism *Methylosinus sporium*, especially due to accumulation of ${\text{NO}}_{2}^{-}$ (He et al., 2017). The pMMO enzyme catalyzing the first step of CH_4_ oxidation in methanotrophs, also oxidizes NH_3_ (${\text{NH}}_{4}^{+}$) to hydroxylamine (NH_2_OH; Hanson and Hanson, 1996; Nyerges and Stein, 2009; Stein and Klotz, 2011; Stein et al., 2012). Ammonia-oxidizers can convey electrons from hydroxylamine oxidation to the quinone pool to conserve energy and support cellular growth (Klotz and Stein, 2008), but methanotrophs lack this system and cannot conserve energy from this oxidation. Since the intermediate NH_2_OH is highly toxic, methanotrophs use mechanisms to quickly detoxify it. In the natural environment strain SolV cells are faced with 1–28 mM ${\text{NH}}_{4}^{+}$ concentrations (Khadem et al., 2010) meaning that the cells have to balance assimilation and tolerance in response to reactive-N molecules. Detoxification can be achieved by conversion of NH_2_OH back to ${\text{NH}}_{4}^{+}$ or to ${\text{NO}}_{2}^{-}$ using a hydroxylamine dehydrogenase enzyme. Nitrite, which is also toxic, can be further converted to nitrous oxide (N_2_O) via toxic nitric oxide (NO) by denitrification enzymes under anoxic conditions (Campbell et al., 2011). Recently, Kits et al. (2015) reported the reduction of nitrate coupled with aerobic methane oxidation under extreme oxygen limited conditions in which N_2_O production was directly supported by CH_4_ oxidation in *Methylomonas denitrificans* strain FJG1^T^.

In the genome of strain SolV, genes encoding enzymes responsible for ${\text{NO}}_{2}^{-}$ reduction (*nirK)* and NO reduction (*norB* encoding the catalytic subunit, *norC* encoding the electron-accepting subunit), were identified but the gene encoding N_2_O reductase was absent. A *haoAB* gene cluster encoding hydroxylamine dehydrogenase was also identified, suggesting the ability of nitrification and handling of reactive N-compounds (Khadem et al., 2012c; Anvar et al., 2014). Previously a pH of 2–3 has been used for physiological studies of strain SolV (Khadem et al., 2010, 2011, 2012a,b,c). However, since strain SolV has a rather broad pH range for growth (Pol et al., 2007) and can be easily adapted to grow at higher pH values, we used the pH range of 5–6 in the present study. This minimized the chemical conversion of ${\text{NO}}_{2}^{-}$ occurring at acidic pH (Matthew et al., 2005; Ryabenko et al., 2009).

Recently, using growth experiments (batch and continuous cultures) together with transcriptome and kinetics analyses, *M. fumariolicum* SolV was shown to be able to grow as a real “Knallgas” bacterium on hydrogen/carbon dioxide, without addition of CH_4_ (Mohammadi et al., 2017). Cells grown on H_2_ still express active pMMO similar to the CH_4_ culture (Mohammadi et al., 2017). Since we hypothesized that the ${\text{NH}}_{4}^{+}$ oxidation is limited by the presence of CH_4_, we tested ${\text{NH}}_{4}^{+}$ oxidation to ${\text{NO}}_{2}^{-}$ using a continuous culture grown on hydrogen in the absence of CH_4_ (Mohammadi et al., 2017). Furthermore, we examined the affinity of cells for ${\text{NH}}_{4}^{+}$ using batch cultures with different concentrations of CH_4_ in a range of 0.5–8% (v/v). The aim of this study was first to investigate whether strain SolV can perform ${\text{NH}}_{4}^{+}$ oxidation, and secondly, how it could detoxify the reactive N-compounds resulting from this oxidation using physiological experiments and transcriptome analysis.

## Materials and methods

### Microorganism and medium composition

*M. fumariolicum* strain SolV used in this study was initially isolated from the volcanic region Campi Flegrei, near Naples, Italy (Pol et al., 2007). In this study the medium to obtain an OD_600_ of 1.0 was composed of 0.2 mM MgCl_2_.6H_2_O; 0.2 mM CaCl_2_.2H_2_O; 1 mM Na_2_SO_4_; 2 mM K_2_SO_4_; 2 mM (NH_4_)_2_SO_4_ (or 5 mM KNO_3_) and 1 mM NaH_2_PO_4_.H_2_O. A trace element solution containing 1 μM NiCl_2_, CoCl_2_, MoO_4_Na_2_, ZnSO_4_ and CeCl_3_; 5 μM MnCl_2_ and FeSO_4_; 10 μM CuSO_4_ and 40–50 μM nitrilotriacetic acid (NTA). The pH of medium was adjusted to 2.7 using 1 M H_2_SO_4_ (1 ml H_2_SO_4_ per 1 L medium). To avoid precipitation, CaCl_2_.2H_2_O and the rest of medium were autoclaved separately and mixed after cooling. This medium composition was used in batch and continuous cultures, unless otherwise stated.

### Chemostat cultivation

The continuous culture with CH_4_ as an electron donor and nitrate (${\text{NO}}_{3}^{-}$) as N-source (CH_4_/${\text{NO}}_{3}^{-}$), liquid volume 500 ml, was operated at 55°C with stirring at 900 rpm with a stirrer bar. The chemostat was supplied with medium at a flow rate of 14.5 ml.h^−1^ (*D* = 0.026 h^−1^), using a peristaltic pump. The cell-containing medium was removed automatically from the chemostat by a peristaltic pump when the liquid level reached the 500 ml level sensor in the reactor. A supply of 10% CH_4_ (v/v), 8% O_2_ (v/v), and 68% CO_2_ (v/v) took place by mass flow controllers through a sterile filter and was sparged into the medium just above the stirrer bar (total gas flow rate ≈20 ml.min^−1^). The initial pH was 3.4 and was regulated with 1 M carbonate connected to the vessel by a peristaltic pump. The pH was gradually increased to 6 and after obtaining a steady state, all experiments were performed at this pH. In the continuous culture with H_2_ as an electron donor and ${\text{NH}}_{4}^{+}$ as N-source (H_2_/${\text{NH}}_{4}^{+}$), liquid volume was 1.2 L and this culture was operated at 55°C with stirring at 1,000 rpm. The chemostat was supplied with medium at a flow rate of 29.9 ml.h^−1^ (*D* = 0.023 h^−1^). A gas supply of 12% H_2_ (v/v), 10% air (v/v), and 5% CO_2_ (v/v) was provided by mass flow controllers through a sterile filter and sparged into the medium (total gas flow rate ≈16.5 ml.min^−1^). The initial pH was 2.9 and the pH was regulated by 1 M NaOH. A pH range from 3 to 5.5 was investigated in the steady state. In the continuous culture with CH_4_ as an electron donor and ${\text{NH}}_{4}^{+}$ as N-source (CH_4_/${\text{NH}}_{4}^{+}$), the liquid volume was 0.3 L and the culture was operated at 55°C with stirring at 700 rpm at pH 2.7. The chemostat was supplied with medium at a flow rate of 0.35 ml.h^−1^ (*D* = 0.0012 h^−1^). A gas supply of 0.16% CH_4_ (v/v), 0.6% O_2_ (v/v), and 5% CO_2_ (v/v) was directed by mass flow controllers through a sterile filter and sparged into the medium (total gas flow rate ≈10 ml.min^−1^). An O_2_ sensor in the liquid was coupled to a Biocontroller (Applikon) regulating the O_2_ mass controller in each reactor.

### Batch cultivation

In order to obtain cells growing at maximum growth rate (μ_max_), cells were grown without any limitation in 250-ml serum bottles containing 40 ml medium (4 mM ${\text{NH}}_{4}^{+}$; pH 2.7), and sealed with red butyl rubber stoppers. The headspace contained air with (v/v) 10% CH_4_, 5% CO_2_ at 55°C with shaking at 250 rpm. Incubations were performed in duplicate.

### Gas analysis

Nitric oxide and nitrous oxide (NO and N_2_O) were analyzed on an Agilent series 6890 gas chromatograph (Agilent, USA) equipped with a Porapak Q and a Molecular sieve column, coupled to a thermal conductivity detector and a mass spectrometer (MS; Agilent 5975 Cinert MSD; Agilent, USA) as described before (Ettwig et al., 2008). For all gas analyses, 100 μl gas samples were injected into the gas chromatograph. Furthermore, nitric oxide production was monitored directly from the gas outlet of the reactors using a nitric oxide analyzer (NOA 280i, GE) with a suction rate of 11.6 ml.min^−1^.

### Dry-weight determination and elemental analysis

To determine the dry weight, samples of 8–10 ml from the culture suspension were filtered through pre-weighed 0.45 μm filters and dried to constant weight in a vacuum oven at 70°C (*n* = 3). In order to determine the total content of carbon and nitrogen, 10 ml of the culture suspension (duplicate) was centrifuged at 4,500 g for 30 min and the clear supernatant was used for the analysis. The nitrogen and carbon content in the supernatant was compared with the corresponding values in the whole cell suspension. The total carbon and nitrogen contents were measured using TOC-L and TNM-1 analyzers (Shimadzu).

### Nitrite, ammonium, and hydroxylamine analysis

To determine nitrite (${\text{NO}}_{2}^{-}$) concentrations, 50 μl of sample, and 450 μl of MilliQ water were added to a cuvette. Then, 500 μl of reagent A [1% (w/v) sulfanilic acid in 1M HCl; kept in the dark] and 500 μl of reagent B [0.1% (w/v) naphtylethylene diaminedihydrochloride (NED) in water; kept at 4°C in the dark] were added to the same cuvette and mixed well. After incubation for 10 min at room temperature, the absorbance at 540 nm was measured and the values were compared with a calibration curve using known concentrations of nitrite in a range of 0–0.5 mM. If necessary, the sensitivity of this assay could be increased 10-fold using 500 μl samples without addition of water. ${\text{NH}}_{4}^{+}$ concentrations were measured using the ortho phthaldialdehyde (OPA) method (Taylor et al., 1974). In order to determine hydroxylamine concentrations, 200 μl reagent A (50 mM potassium phosphate buffer pH 7), 160 μl demineralized water, 200 μl sample, 40 μl reagent B [12% (w/v) trichloroacetic acid in water, kept in the dark], 200 μl reagent C (1% w/v 8-hydroxyquinoline (quinolinol) in 100% ethanol, kept in the dark) and 200 μl reagent D (1 M Na_2_CO_3_) were mixed and incubated at 100°C for 1 min. The absorption was measured at 705 nm and the values were compared to a calibration curve using hydroxylamine concentrations 0.02–0.1 mM.

### Activity assays

To determine the affinity constant of pMMO for NH_3_ of each sample, a volume of 5 ml of cells from the CH_4_/${\text{NO}}_{3}^{-}$ continuous culture were washed and resuspended in the same medium at pH 6 (The pH of the medium was adjusted to 6 using MES buffer at a final concentration of 25 mM), transferred to a 60-ml serum bottle and capped. After a pre-incubation for 30 min, CH_4_ was added to each bottle at final concentrations of 0.5, 1, 2, 3, 4, and 8% (v/v). To each incubation, with a certain concentration of CH_4_, ${\text{NH}}_{4}^{+}$ was added in a range of 0.5–16 mM. The initial production of ${\text{NO}}_{2}^{-}$ was measured, and the values were normalized to the total protein content of the cells. Incubations were performed at 55°C and shaking at 380 rpm. Each condition was performed in duplicate and values did not deviate more than 5%.

### RNA isolation and transcriptome analysis

The complete genome sequence of strain SolV (Anvar et al., 2014), which is also available at the MicroScope annotation platform (https://www.genoscope.cns.fr/agc/microscope/home/), was used as the template for the transcriptome analysis (RNA-seq). A 4-ml volume of cells (OD_600_ = 1) was sampled from the continuous cultures (H_2_ and CH_4_ grown cells under O_2_ limitation) and from a batch culture (cells at μ_max_ grown on CH_4_ without limitation) and harvested by centrifugation. The pellet was further used for mRNA isolation using the RiboPure™-Bacteria Kit according to the manufacturer's protocol (ThermoFisher, Waltham, USA). Briefly, cells were disrupted by cold Zirconia beads and after centrifugation, 0.2 volumes of chloroform was added to the supernatant for initial RNA purification. Next, 0.5 volumes of 100% ethanol was added to the aqueous phase obtained after chloroform addition and the whole sample was transferred to a filter cartridge. After washing, the RNA was eluted from the filter cartridge. Afterwards, using MICROB*express*™ kit (ThermoFisher, Waltham, USA) the ribosomal RNAs were removed from the total RNA. The rRNA removal efficiency was checked using the Agilent 2100 Bioanalyzer (Agilent, Santa Clara, USA). Next, Ion Total RNA-Seq Kit v2 (ThermoFisher, Waltham, USA) was used to construct the cDNA libraries from rRNA-depleted total RNA. Briefly, the rRNA-depleted total RNA was fragmented using RNase III and then, reverse transcription was performed on the fragmented RNAs. The obtained cDNAs were amplified and further purified to prepare barcoded libraries. To prepare the template for the Ion Personal Genome Machine® (PGM™) System, a volume of 15 μl from two sample libraries with a concentration of 14 pM were mixed. This mixture of two libraries was used to prepare the template-positive Ion Sphere™ particles (ISPs) using the Ion OneTouch™ 2 instrument. Afterwards, the template-positive ISPs were enriched using the Ion OneTouch™ ES instrument. Both template preparation and enrichment were performed using the Ion PGM™ Template OT2 200 Kit (Ion Torrent, Life technologies). Enriched templates were sequenced on an Ion 318™ Chip v2 using the Ion PGM™ sequencing 200 Kit v2. Expression analysis was performed with the RNA-seq Analysis tool from the CLC Genomic Work bench software (version 7.0.4, CLC-Bio, Aarhus, Denmark). The sequencing reads were first mapped to the ribosomal RNA operon and all tRNA and ncRNA genes, and mapped reads were discarded. The remaining reads were mapped to the CDS sequences extracted from the genome sequence of strain SolV (Anvar et al., 2014). Expression values are given as RPKM (Reads per Kilo base of exon model per Million mapped reads; Mortazavi et al., 2008). The total number of reads obtained and mapped on the coding sequences of the genome for each sample together with the calculated expression levels (RPKM) is provided in the Supplementary Material (Table S1).

## Results

### Physiological tests regarding ammonium oxidation to nitrite and nitrite reduction to N_2_O

**Figure 1:**
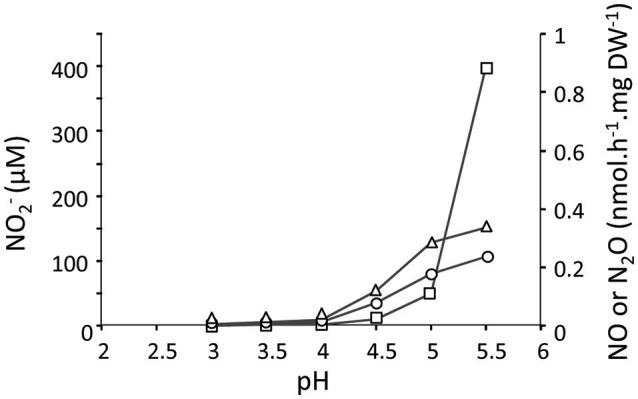
The concentration of ${\text{NO}}_{2}^{-}$ (open rectangles) and production rates of NO (open triangles) and N_2_O (open circles) at pH values from 3 to 5.5 in the H_2_/${\text{NH}}_{4}^{+}$ continuous culture (1.2 L; *D* = 0.023 h^−1^; OD_600_ = 0.85; O_2_ limited; 4 mM ${\text{NH}}_{4}^{+}$). The amounts of ${\text{NO}}_{2}^{-}$, NO and N_2_O were determined when cells in the reactor reached the steady state. Each data point represents the average of two replicates with deviation of individual values below 5%.

**Figure 2:**
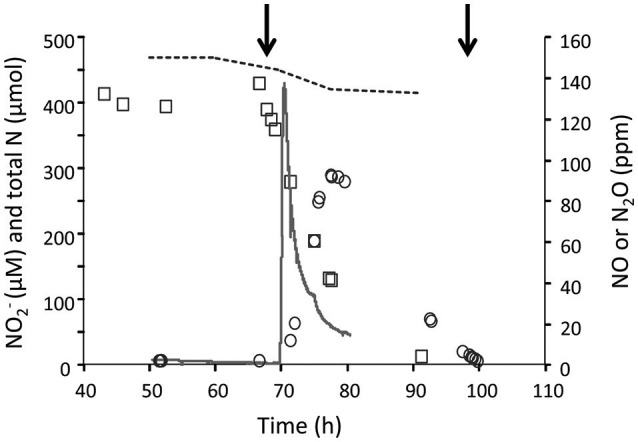
The concentrations of ${\text{NO}}_{2}^{-}$, NO and N_2_O in the H_2_/ ${\text{NH}}_{4}^{+}$ continuous culture at pH 5.5 (1.2 L; *D* = 0.023 h^−1^; OD_600_ = 0.85; O_2_ limited; 4 mM ${\text{NH}}_{4}^{+}$). Nitrite (open rectangles), NO (solid line), N_2_O (open circles) were determined under O_2_ limitation and anoxic conditions. The first arrow indicates the oxic to anoxic, the second arrow indicates the anoxic to oxic condition and the dashed line shows the total N during the experimental phase. The decrease of N_2_O levels was because of the continuous dilution of the gas present in the reactor headspace (total gas flow rate in the outlet ≈15 ml.min^−1^).

To study the effect and conversion of nitrogenous compounds, three different continuous cultures were used which are referred to as CH_4_/${\text{NH}}_{4}^{+}$, H_2_/${\text{NH}}_{4}^{+}$, and CH_4_/${\text{NO}}_{3}^{-}$. In the second and third cultures, oxygen was limiting. Using a NOx analyzer and GC-MS, we demonstrated that in the CH_4_/${\text{NH}}_{4}^{+}$ culture with low actual CH_4_ concentrations in the liquid (0.3 μM) and with ${\text{NH}}_{4}^{+}$ (4 mM), ${\text{NO}}_{2}^{-}$ was not detected, and N_2_O production rate was only 0.015 nmol N_2_O.h^−1^.mg DW^−1^ (Table 1) which was 12,000-fold less than the CH_4_ conversion rate (180 nmol.h^−1^.mg DW^−1^). To increase ${\text{NO}}_{2}^{-}$ concentrations and study potential toxic effects of this compound, we used the H_2_/${\text{NH}}_{4}^{+}$ continuous culture applying different conditions. Initially, the production of ${\text{NO}}_{2}^{-}$, NO, and N_2_O were measured under steady state conditions at a pH range of 3–5.5 under O_2_ limitation (Figure 1). We showed that the ${\text{NO}}_{2}^{-}$, NO and N_2_O concentrations were elevated by increasing the pH from 3 to 5.5 in the presence of 4 mM ${\text{NH}}_{4}^{+}$. Changing pH from 3 to 5.5 introduces more NH_3_ in the medium. The NH_3_ concentration in a range of 12 nM to 5 μM was calculated using the Henderson–Hasselbalch equation (Hütter, 1992), considering the temperature of 55°C at pH 3 to 5.5, respectively. At pH 5.5, we measured a ${\text{NO}}_{2}^{-}$ concentration at steady state of about 420 μM in the reactor (Figure 2) resulting from a production rate of ≈ 48 nmol ${\text{NO}}_{2}^{-}$.h^−1^.mg DW^−1^, while nitrite production at pH 3 was very limited. Based on the clear effect of increasing pH on the production of ${\text{NO}}_{2}^{-}$, one could speculate that the real substrate for pMMO to produce ${\text{NO}}_{2}^{-}$ is NH_3_ (not ${\text{NH}}_{4}^{+}$). Furthermore, the ${\text{NO}}_{2}^{-}$ reduction activities (NO and N_2_O production) were measured at 0.81 nmol ${\text{NO}}_{2}^{-}$.h^−1^.mg DW^−1^ (1.7% of ${\text{NH}}_{4}^{+}$ oxidation rate) which is 53-fold higher than that in the CH_4_/${\text{NH}}_{4}^{+}$ culture (Table 1). A rapid ${\text{NO}}_{2}^{-}$ consumption (≈83 nmol ${\text{NO}}_{2}^{-}$.h^−1^.mg DW^−1^) was observed when O_2_ supply was switched off completely (Figure 2), and the ${\text{NO}}_{2}^{-}$ reduction rate (as NO and N_2_O) increased about 100-fold (74.4 nmol ${\text{NO}}_{2}^{-}$.h^−1^.mg DW^−1^). A rapid initial increase of NO suggests that conversion to N_2_O is the rate limiting step. The decrease of N_2_O levels was due to the continuous dilution of the gas present in the reactor headspace (total gas flow rate in the outlet ≈ 15 ml.min^−1^). Concentrations of 1–5 μM NH_2_OH were measured in data points before and after switching off O_2_ supply.

**Table 1:** Overview of ${\text{NH}}_{4}^{+}$ oxidation and ${\text{NO}}_{2}^{-}$ reduction rates calculated in each continuous culture at two different pH values.

	**Continuous cultures**			
	**CH_4_/**${\text{NH}}_{4}^{+}$		**H_2_/**${\text{NH}}_{4}^{+}$	
	**pH 3**	**pH 5.5**	**pH 3**	**pH 5.5**
${\text{NH}}_{4}^{+}$ (NH_3_)^a^	4 (0.02)	4 (5)	4 (0.02)	4 (5)
${\text{NH}}_{4}^{+}$ oxidation^b^	BDL^d^	ND^e^	0.12^f^	48.2
${\text{NO}}_{2}^{-}$ reduction^b^	ND	ND	BDL	0.8
${\text{NO}}_{2}^{-}$ reduction^b^^c^	0.015	ND	0.011	74.4

a *${\mathrm{NH}}_{4}^{+}$ and NH_3_ concentrations are in mM and μM, respectively*.

b *${\mathrm{NO}}_{2}^{-}$ production and N_2_O production values are in nmol.h^−1^.mg DW^−1^*.

c *${\mathrm{NO}}_{2}^{-}$ reduction rates under anoxic conditions*.

d *BDL, below detection limit*.

e *ND, not determined*.

f *All values are the average of two replicates of the same continuous culture with <5% difference between duplicates*.

**Figure 3:**
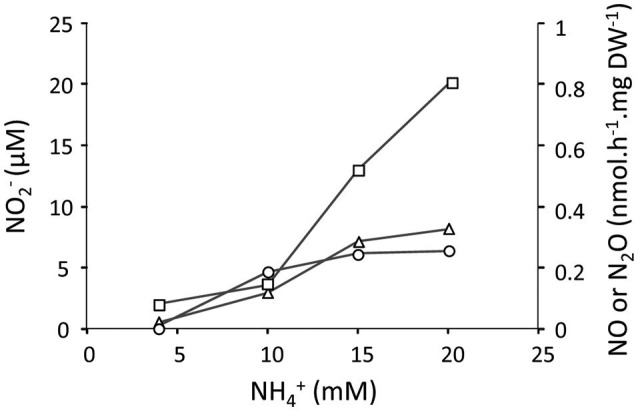
The concentration of ${\text{NO}}_{2}^{-}$ (open rectangles) and production rates of NO (open triangles) and N_2_O (open circles) in the H_2_/ ${\text{NH}}_{4}^{+}$ chemostat culture (1.2 L; *D* = 0.023 h^−1^; OD_600_ = 0.85; O_2_ limited) at ${\text{NH}}_{4}^{+}$ concentrations ranging 4–20 mM at pH 4. The amounts of ${\text{NO}}_{2}^{-}$, NO, and N_2_O were determined when cells in the reactor reached the steady state. Each data point represents the average of two replicates with deviation of individual values below 5%.

**Figure 4:**
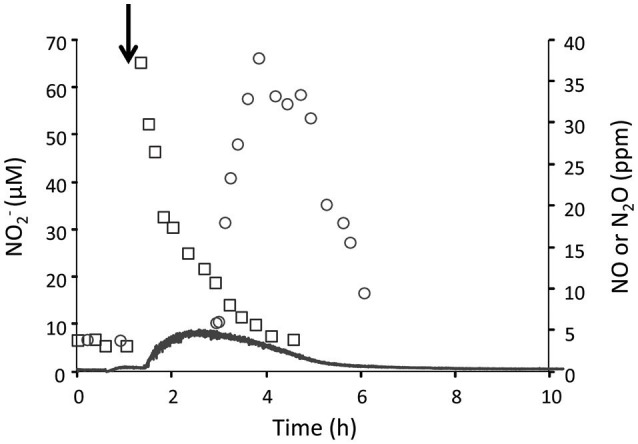
Cells from the CH_4_/${\text{NO}}_{3}^{-}$ continuous culture (0.6 L; *D* = 0.026 h^−1^; OD_600_ = 1.3; O_2_ limited) perform denitrification when nitrite was added to the reactor vessel. The concentrations of ${\text{NO}}_{2}^{-}$ (open rectangles), NO (solid line) and N_2_O (open circles) were measured before and after addition of 50 μM ${\text{NO}}_{2}^{-}$ (arrow). Duplicates of individual values do not deviate more than 5 and 10% for ${\text{NO}}_{2}^{-}$ and N_2_O, respectively.

We further tested the effect of different concentrations of ${\text{NH}}_{4}^{+}$ (4–20 mM) on the ${\text{NO}}_{2}^{-}$, NO and N_2_O production at pH 4 under oxygen limitation in the H_2_/${\text{NH}}_{4}^{+}$ continuous culture (Figure 3). We showed that the concentrations of ${\text{NO}}_{2}^{-}$, NO, and N_2_O slightly increased once the ${\text{NH}}_{4}^{+}$ concentration was gradually elevated. This observation indicates that at pH 4, even a 4-fold increase in the ${\text{NH}}_{4}^{+}$ concentration did not result in a high production of ${\text{NO}}_{2}^{-}$ similar to what we observed at pH 5.5 supporting our assumption that pH plays an important role regarding the availability of NH_3_ molecules. Furthermore, we showed that the cells in the CH_4_/${\text{NO}}_{3}^{-}$ continuous culture were able to perform ${\text{NO}}_{2}^{-}$ reduction at a rate of 120 nmol ${\text{NO}}_{2}^{-}$.h^−1^.mg DW^−1^ by converting the added ${\text{NO}}_{2}^{-}$ (50 μM) to NO and further to N_2_O in the absence of oxygen (Figure 4). Table 1 shows an overview of rates of ammonium oxidation to nitrite and nitrite reduction to NO/N_2_O) in the different continuous culture.

### Kinetics of ammonia oxidation

The affinity constants (K_s_) for ${\text{NH}}_{4}^{+}$ and NH_3_ were determined using SolV cells from the CH_4_/${\text{NO}}_{3}^{-}$ continuous culture. From the initial production rates of nitrite the best fitting curves to Michaelis–Menten kinetics were predicted (Figure S1). Since part of the ${\text{NH}}_{4}^{+}$ is present as NH_3_ at pH 6 (1 M ${\text{NH}}_{4}^{+}$ is about 3 mM NH_3_ at pH 6), the Michaelis–Menten curves were also produced based on the NH_3_ concentrations (Figure S1). Therefore, we calculated apparent affinity constants (K_s_) for both ${\text{NH}}_{4}^{+}$ and NH_3_ in strain SolV (Table 2).

**Table 2:** Kinetics of ${\text{NH}}_{4}^{+}$ oxidation with variable CH_4_ supply at pH 6.

**CH_4_**	**Affinity constant^c^ [K_s(app)_]**		**V_max_^d^**
	**${\text{NH}}_{4}^{+}$ (mM)**	**NH_3_ (μM)**	
0.5^a^(0.005)^b^	1.25	4.9	1.61
1 (0.01)	1.50	5.8	1.61
2 (0.02)	6	23.3	1.43
3 (0.03)	9	35.0	1.43
4 (0.04)	30	116.7	1.43
8 (0.08)	70	272.3	1.43

a *CH_4_ concentrations in % (v/v)*.

b *CH_4_ concentrations in the liquid in mM*.

c Affinity constants were calculated based on two independent experiments

d *V_max_ values are in μmol ${\mathrm{NO}}_{2}^{-}$.h^−1^.mg protein^−1^*.

To identify the type of inhibition, the Michaelis–Menten curves were transformed to Lineweaver-Burk plots. Figure S2 shows a set of double reciprocal plots, obtained with different ${\text{NH}}_{4}^{+}$ concentrations in the presence of CH_4_ at a range of 2, 4, and 8% (v/v). Increasing the CH_4_ concentration resulted in a group of lines with a common intercept on the 1/V_0_ axis but with different slopes. The intercept is 1/V_max_ and V_max_ is constant regardless of increasing CH_4_ concentration (V_max_ = 1.61 ± 0.05 μmol.h^−1^.mg protein^−1^). The constant intercept of all lines suggests a competitive inhibition between CH_4_ and NH_3_. The ${\text{NO}}_{2}^{-}$ production rate in the absence of CH_4_ was about 3- to 4-fold lower compared to the rate in the presence of 0.5% (v/v) CH_4_ suggesting that traces of CH_4_ are essential for the pMMO activation. Table 2 shows an overview of affinity constants calculated for NH_3_ (${\text{NH}}_{4}^{+}$) obtained in the incubations with different CH_4_ concentrations. Affinity constants for NH_3_ were calculated based on the Henderson–Hasselbalch equation considering a temperature of 55°C (Hütter, 1992). These results showed that increasing CH_4_ concentration limits the affinity of pMMO for NH_3_ significantly, which correlates with the observed competitive inhibition between CH_4_ and NH_3_.

### Whole genome transcriptome analysis of strain SolV

Expression levels of housekeeping genes and genes involved in metabolism of nitrogenous compounds were determined for H_2_- and CH_4_-grown cells (both under O_2_ limited conditions). These values were compared to the expression values in cells growing at μ_max_ on CH_4_ (without limitation). To compare baseline expression levels, we selected a group of 384 housekeeping genes (in total 437.9 kbp) involved in energy generation, ribosome assembly, carbon fixation (CBB cycle), C1 metabolism (except for *pmo*), amino acid synthesis, cell wall synthesis, translation, transcription, DNA replication, and tRNA synthesis (Khadem et al., 2012a,b). All ratios of expression levels of the housekeeping genes under these conditions were between 0.5 and 2 (Table S1). The robustness of the transcriptome data were tested using the method of Chaudhuri et al. (2011). In this method, the logarithmic value of RPKM + 1 of each condition (in duplicates) was calculated and the values were plotted against each other. This resulted in correlation coefficients of 0.80, 0.82, and 0.87 (Figure S3), showing the high robustness of the transcriptome data.

The transcriptome data showed that genes encoding the enzymes involved in ${\text{NH}}_{4}^{+}$ assimilation in strain SolV including glutamine synthase (GlnA)/glutamate synthase (GltB) and the alanine and glutamate dehydrogenases (Ald, Gdh) were equally expressed under all conditions (Table 3). Among these genes, only *glnA* was about 2.5-fold less expressed in the continuous cultures compared to the cells grown at μ_max_ (Table 3). We also found that the *carAB* operons (encoding the glutamine hydrolyzing carbamoyl-phosphate synthase) were constitutively expressed. The conversion of glutamine and carbon dioxide into glutamate and carbamoyl phosphate is performed by this enzyme (Khadem et al., 2012a). Similarly, the *argDHFG* operons (encoding enzymes from the urea cycle) were expressed under all conditions. Interestingly, we detected the ammonium/ammonia transporter (*amtB*) was at least 3-fold up-regulated in the CH_4_/${\text{NO}}_{3}^{-}$ continuous culture compared to the other conditions reflecting that cells may have a preference for ${\text{NH}}_{4}^{+}$ as N-source. In addition, the genes encoding the ${\text{NO}}_{3}^{-}$/${\text{NO}}_{2}^{-}$ transport (*nasA*) and the assimilatory nitrite and nitrate reductases were 9- to 45-fold up-regulated in the CH_4_/${\text{NO}}_{3}^{-}$ continuous culture compared to the cells at μ_max_ (Table 3). Both latter observations correlate with the fact that nitrate was used as N-source under this condition. Interestingly, the transcriptome analysis showed that the *nirK* and *norC* genes were up-regulated in the chemostat continuous culture compared to those at μ_max_, while results for *norB* (encoding the catalytic subunit) were less clear. This may imply that other NO reductases were active. We also found that the *haoA* gene was about 2-fold down-regulated in the CH_4_/${\text{NO}}_{3}^{-}$ continuous culture compared to the H_2_/${\text{NH}}_{4}^{+}$ and μ_max_ cultures, likely due to the absence of ${\text{NH}}_{4}^{+}$ in this condition. The *haoA* gene showed comparable high expression levels in the H_2_/${\text{NH}}_{4}^{+}$ continuous and batch μ_max_ culture (Table 3).

**Table 3:** The transcriptome analysis of the genes involved in nitrogen metabolism in *Methylacidiphilum fumariolicum* SolV.

**Enzyme**	**Gene name**	**GenBank identifier**	**Expression level (RPKM)^a^**		
			**H_2_/${\text{NH}}_{4}^{+}$**	**CH_4_/${\text{NO}}_{3}^{-}$**	**Cells at μ_max^b^_**
Glutamine synthetase type I (EC 6.3.1.2)	*glnA*	Mfumv2_1420	764	893	2,065
Glutamine synthetase regulatory protein PII	*glnB*	Mfumv2_1419	943	719	883
[Protein-PII] uridylyltransferase (EC 2.7.7.59)	*glnD*	Mfumv2_1837	124	136	156
Nitrogen regulatory protein PII	*glnK*	Mfumv2_1285	371	125	193
Alanine dehydrogenase (EC 1.4.1.1)	*ald*	Mfumv2_2049	107	106	171
Glutamate dehydrogenase (EC 1.4.1.2; EC 1.4.1.4)	*gdhA*	Mfumv2_0663	227	231	421
Glutamate synthase [NADPH] large chain (EC 1.4.1.13)	*gltB*	Mfumv2_2397	906	696	1,300
Glutamate synthase beta chain	*gltD*	Mfumv2_1978	192	328	198
Ornithine-acetylornithine aminotransferase (EC 2.6.1.11)	*argD1*	Mfumv2_1148	279	271	627
Ornithine-acetylornithine aminotransferase (EC 2.6.1.11)	*argD2*	Mfumv2_0135	145	273	357
Argininosuccinate lyase (EC 4.3.2.1)	*argH*	Mfumv2_2465	78	68	203
Ornithine carbamoyltransferase (EC 2.1.3.3)	*argF*	Mfumv2_0136	161	239	278
Argininosuccinate synthase (EC 6.3.4.5)	*argG*	Mfumv2_1907	666	654	645
Carbamoyl-phosphate synthase small chain (EC 6.3.5.5)	*carA*	Mfumv2_1926	318	350	453
Carbamoyl-phosphate synthase large chain (EC 6.3.5.5)	*carB*	Mfumv2_0408	347	674	514
Ammonium-Ammonia transporter	*amtB*	Mfumv2_1275	294	1,082	391
Nitrate ABC transporter, nitrate-binding protein	*tauA*	Mfumv2_1299	28	41	34
Assimilatory nitrate reductase catalytic subunit (EC 1.7.99.4)	*nasC*	Mfumv2_1297	20	105	13
Nitrate-nitrite transporter	*nasA*	Mfumv2_1294	67	321	23
Nitrite reductase [NAD(P)H] large subunit (EC 1.7.1.4)	*nirB*	Mfumv2_1296	140	854	19
Nitrite reductase [NAD(P)H], small subunit (EC 1.7.1.4)	*nirD*	Mfumv2_1295	63	308	33
Signal transduction histidine kinase with PAS domain	*ntrB*	Mfumv2_0271	275	180	291
Signal transduction response regulator, NtrC family	*ntrC1*	Mfumv2_1349	98	84	103
Sigma-54 dependent transcriptional regulator-response regulator	*ntrC2*	Mfumv2_1221	65	59	100
Transcriptional regulator, NifA subfamily, Fis Family	*ntrC3*	Mfumv2_2103	581	400	533
Sigma-54 dependent transcriptional regulator-response regulator	*ntrC4*	Mfumv2_0272	264	387	293
Hydroxylamine dehydrogenase (EC 1.7.2.6)	*haoA*	Mfumv2_2472	402	109	351
Hydroxylamine dehydrogenase associated protein	*haoB*	Mfumv2_2471	179	163	302
Nitric-oxide reductase subunit B (EC 1.7.99.7)	*norB*	Mfumv2_0037	125	84	178
Nitric-oxide reductase subunit C (EC 1.7.99.7)	*norC*	Mfumv2_0036	429	372	197
Copper-containing nitrite reductase (EC 1.7.2.1)	*nirK*	Mfumv2_1973	379	520	136
DNA-binding response regulator, NarL family	*mxaB*	Mfumv2_1738	163	291	288
DNA-binding response regulator, LuxR family	*citB1*	Mfumv2_1799	7,016	4,126	1,063
DNA-binding response regulator, LuxR family	*citB2*	Mfumv2_0457	137	133	307

a *The mRNA expression is shown as RPKM according to Mortazavi et al. (2008). Changes in expression in the continuous cultures (H_2_/${\mathrm{NH}}_{4}^{+}$ and CH_4_/${\mathrm{NO}}_{3}^{-}$) compared to batch culture cells growing at μ_max_ are demonstrated by shading [up-regulation >2-fold dark gray; down-regulation <0.5 (light gray)]*.

b *Cells grown on CH_4_ with ${\mathrm{NH}}_{4}^{+}$ as N-source*.

The transcriptome data showed different expression levels of two of the three different *pmo* operons in strain SolV (Table 4). We found that the *pmo*CAB2 operon including the mfumv2_1793, mfumv2_1792 and mfumv2_1791 subunits was significantly expressed (RPKM values 14,899–37,218) in the cells growing at μ_max_ with no limitation and the *pmo*CAB1 operon showed very low expression. In contrast, cells in the continuous cultures on H_2_/${\text{NH}}_{4}^{+}$ and CH_4_/${\text{NO}}_{3}^{-}$ under O_2_ limitation showed a significantly different expression pattern of the *pmoCAB* operons. We found that the *pmoCAB*1 operon including mfumv2_1796, mfumv2_1795 and mfumv2_1794 subunits was very highly expressed under these conditions (RPKM values 5,003–47,785), whereas the expression levels of the *pmoCAB*2 operon was found to be 2- to 19-fold lower in comparison to the cells growing at μ_max_. The *pmoCAB*3 operon including the mfumv2_1606, mfumv2_1605 and mfumv2_1604 subunits showed low expressed under all conditions although expression in H_2_/${\text{NH}}_{4}^{+}$ grown cells seems to be slightly up-regulated. The conversion of methanol to formaldehyde is the second step in CH_4_ oxidation pathway. Interestingly, it has been shown that strain SolV contains a XoxF-type methanol dehydrogenase (MDH) that can convert methanol directly to formate (Pol et al., 2014). We found that the *xoxFGJ* operon encoding the methanol dehydrogenase and *pqqABCDEF* operon encoding the proteins involved in biosynthesis of the methanol dehydrogenase cofactor pyrroloquinoline quinone were expressed more or less similar under all conditions tested. The last step of the CH_4_ oxidation pathway is conversion of formate to CO_2_ catalyzed by NAD-dependent formate dehydrogenase and a membrane-bound formate dehydrogenase. The genes encoding these enzymes were expressed under all conditions, although the expression levels of these enzymes (except for *fdsD* and *fdh*) in continuous cultures under O_2_ limitation was 2- to 2.5-fold lower compared to cells grown at μ_max_ (Table 4).

**Table 4:** The transcriptome analysis of the genes involved in the methane oxidation pathway of *Methylacidiphilum fumariolicum* SolV.

**Enzyme**	**Gene name**	**GenBank identifier**	**Expression level (RPKM)^a^**		
			**H_2_/${\text{NH}}_{4}^{+}$**	**CH_4_/${\text{NO}}_{3}^{-}$**	**Cells at μ_max^b^_**
Particulate CH_4_ monooxygenase_1 (EC 1.14.13.25)	*pmoC1*	Mfumv2_1796	47,785	34,734	207
	*pmoA1*	Mfumv2_1795	9,772	3,775	41
	*pmoB1*	Mfumv2_1794	9,550	5,003	164
Particulate CH_4_ monooxygenase_2 (EC 1.14.13.25)	*pmoC2*	Mfumv2_1793	18,136	5,462	37,218
	*pmoA2*	Mfumv2_1792	2,383	1,119	21,207
	*pmoB2*	Mfumv2_1791	2,139	1,265	14,899
Particulate CH_4_ monooxygenase_3 (EC 1.14.13.25)	*pmoC3*	Mfumv2_1606	539	209	181
	*pmoA3*	Mfumv2_1605	143	17	57
	*pmoB3*	Mfumv2_1604	58	13	28
Methanol dehydrogenase XoxF-type (EC 1.1.99.8)	*xoxF*	Mfumv2_1183	6,220	5,291	6,041
Extracellular solute-binding protein family 3	*xoxJ*	Mfumv2_1184	714	1,057	1,478
Cytochrome c1 protein fused with XoxJ	*xoxGJ*	Mfumv2_1185	611	829	1,042
Coenzyme PQQ precursor peptide	*ppqA*	Mfumv2_1461a	2,920	1,919	2,133
Coenzyme PQQ synthesis proteins	*pqqB*	Mfumv2_1461	1,308	588	620
	*pqqC*	Mfumv2_1462	1,165	560	622
	*pqqD*	Mfumv2_0766	144	242	60
	*pqqD*	Mfumv2_1463	451	153	249
	*pqqE*	Mfumv2_1464	747	514	509
	*pqqF*	Mfumv2_0519	408	680	718
NADPH:quinone oxidoreductase (EC 1.6.5.5)	*qor1*	Mfumv2_1937	253	287	315
	*qor2*	Mfumv2_2088	338	300	432
	*qor3*	Mfumv2_0618	60	12	130
Zn-dependent alcohol dehydrogenase (EC 1.1.1.1)	*adh1*	Mfumv2_2176	160	154	208
	*adh2*	Mfumv2_0724	252	218	288
Aldehyde dehydrogenase (EC 1.2.1.3)	*dhaS1*	Mfumv2_2408	130	317	108
	*dhaS2*	Mfumv2_0597	1,310	1,503	1,125
Dihydropteroate synthase (EC 2.5.1.15)	*folP1*	Mfumv2_0503	161	233	167
	*folP2*	Mfumv2_2400	126	95	208
Formate–tetrahydrofolate ligase (EC 6.3.4.3)	*fhs*	Mfumv2_2082	396	457	282
Methylenetetrahydrofolate dehydrogenase (NADP+) (EC 1.5.1.5) - methenyltetrahydrofolate cyclohydrolase (EC 3.5.4.9)	*folD*	Mfumv2_1033	257	173	261
GTP cyclohydrolase I (EC 3.5.4.16) type 2	*folE*	Mfumv2_0074	1,485	1,477	795
NAD-dependent formate dehydrogenase alpha subunit	*fdsA*	Mfumv2_1457	568	665	1,342
NAD-dependent formate dehydrogenase beta subunit	*fdsB*	Mfumv2_1458	569	435	1,149
NAD-dependent formate dehydrogenase gamma subunit	*fdsC*	Mfumv2_1459	475	240	672
NAD-dependent formate dehydrogenase delta subunit	*fdsD*	Mfumv2_1456	593	979	588
NAD-dependent formate dehydrogenase (EC 1.2.1.2)	*fdh*	Mfumv2_1567	738	863	1,110
Methylamine dehydrogenase light chain (EC 1.4.99.3)	*mauA*	Mfumv2_0350	119	450	108
Methylamine dehydrogenase heavy chain (EC 1.4.99.3)	*mauB*	Mfumv2_0347	99	135	235

a *The mRNA expression is shown as RPKM according to Mortazavi et al. (2008). Changes in expression in the continuous cultures (H_2_/${\mathrm{NH}}_{4}^{+}$ and CH_4_/${\mathrm{NO}}_{3}^{-}$) compared to batch culture cells growing at μ_max_ are demonstrated by shading [up-regulation >2-fold (dark gray), down-regulation <0.5 (light gray)]*.

b *Cells grown on CH_4_ with ${\mathrm{NH}}_{4}^{+}$ as N-source*.

## Discussion

In the present study, the physiological data of the H_2_/${\text{NH}}_{4}^{+}$ continuous culture showed that strain SolV is able to oxidize ${\text{NH}}_{4}^{+}$ to ${\text{NO}}_{2}^{-}$ at a rate of 48.2 nmol ${\text{NO}}_{2}^{-}$.h^−1^.mg DW^−1^ at pH 5.5. At pH 3, with less NH_3_ available this rate was about 400-fold lower (Table 1). We also detected a very limited ${\text{NH}}_{4}^{+}$ oxidation rate in the cells of the CH_4_/${\text{NH}}_{4}^{+}$ chemostat in comparison to the H_2_/${\text{NH}}_{4}^{+}$ cells. These observations indicate that the higher ${\text{NH}}_{4}^{+}$ oxidation activity occurs when CH_4_ is replaced by H_2_ as the electron donor. Nitrification was previously reported in methanotrophs. CH_4_-dependent nitrification was detected in a humisol that was enriched with CH_4_ (Megraw and Knowles, 1987). It has been shown that methanotrophs are efficient nitrifiers and produce NH_2_OH as a product of NH_3_ monooxygenation (Bédard and Knowles, 1989; Nyerges and Stein, 2009).

We observed a similar pattern in the batch experiments using cells from the CH_4_/${\text{NO}}_{3}^{-}$ continuous culture. In these batch tests, we found higher ${\text{NO}}_{2}^{-}$ production rates when the CH_4_ concentration was limited, although traces of CH_4_ seemed to be essential for activation of pMMO. In these batch tests, the calculated apparent affinity constants [K_s(app)_] for ${\text{NH}}_{4}^{+}$ were approximately between 1.25 and 70 mM. At increasing pH values the equilibrium shifts toward higher NH_3_ concentrations and the calculated K_s_ values for NH_3_ in the same tests were 4–273 μM. Comparable values have been reported in literature (Table 5). Our data showed that increasing the pH from 3 to 5.5 significantly affects the rates of ${\text{NH}}_{4}^{+}$ oxidation to ${\text{NO}}_{2}^{-}$. This reflects the fact that the pMMO of strain SolV might use NH_3_ as a substrate (and not ${\text{NH}}_{4}^{+}$). This assumption could explain why at low pH, when ${\text{NH}}_{4}^{+}$ is present, we observed very limited nitrification. In a study from O'Neill and Wilkinson (1977), they also showed that by increasing pH the rate of ${\text{NH}}_{4}^{+}$ oxidation by *M. trichosporium* OB3B increased, and they also suggested the active species to be NH_3_.

**Table 5:** Comparison of apparent K_s_ values for ${\text{NH}}_{4}^{+}$.

**Organism**	**K_*s*_ (${\text{NH}}_{4}^{+}$) mM**	**CH_4_ % (v/v)**	**pH**	**Calculated K_*s*_ (NH_3_) μM**	**References**
*M. fumariolicum*	1.25–70	0.5–8	6	4–273	This study^a^
*Mm. album*	2 and 3.9	0.5 and 5	–	–	Nyerges and Stein, 2009
*Methylocystis* sp.	0.5 and 1.1	0.5 and 5	–	–	Nyerges and Stein, 2009
*Ms. trichosporium*	4.1	–	6.5	–	O'Neill and Wilkinson, 1977
	0.6	–	7.5	–	
*Mb. capsulatus*	87^b^	–	7	–	Dalton, 1977

a *See also Table 1*.

b *At ${\mathrm{NH}}_{4}^{+}$ concentrations between 20 and 200 mM, −, not reported*.

In the present study, we showed that strain SolV performs ${\text{NO}}_{2}^{-}$ reduction to N_2_O usingcells from CH_4_/${\text{NH}}_{4}^{+}$ and H_2_/${\text{NH}}_{4}^{+}$ continuous cultures (Table 1). Under anoxic condition, higher ${\text{NO}}_{2}^{-}$ reduction rates were observed in cells from the CH_4_/${\text{NO}}_{3}^{-}$ and H_2_/${\text{NH}}_{4}^{+}$ cultures (Table 1). The reduction of ${\text{NO}}_{2}^{-}$ to N_2_O may provide a way to remove potentially toxic ${\text{NO}}_{2}^{-}$. The lower ${\text{NO}}_{2}^{-}$ reduction rate in H_2_/${\text{NH}}_{4}^{+}$ compared to the CH_4_/${\text{NO}}_{3}^{-}$ continuous cultures in the absence of oxygen could be explained by the fact that cells in the H_2_ reactor were confronted with NH_2_OH and ${\text{NO}}_{2}^{-}$ over a relatively long term. Cells might suffer under these conditions and show a decrease in ${\text{NO}}_{2}^{-}$ reduction rate. Many methanotrophs possess partial denitrification pathways and they are able to reduce ${\text{NO}}_{2}^{-}$ to N_2_O via NO (Nyerges et al., 2010; Campbell et al., 2011). Recently, two methanotrophic strains were cultured together (*Methylomicrobium album* ATCC 33003 and *Methylocystis* sp. strain ATCC 49242), one with high tolerance to ${\text{NH}}_{4}^{+}$ and one with high tolerance to ${\text{NO}}_{2}^{-}$, and the nitrite-tolerant strain was shown to be more competitive and produced more N_2_O compared to the other strain (Nyerges et al., 2010). The highest N_2_O production rate was reported at about 0.4 nmol.h^−1^ per 10^6^ cells in *M. album* ATCC 33003 (Nyerges et al., 2010). Campbell et al. (2011) reported a headspace production of 26.3 μM N_2_O after 48 h (≈0.24 ppb.h^−1^ per 10^6^ cells) in *Methylococcus capsulatus* Bath. Recently, Kits et al. (2015) reported the reduction of nitrate coupled to aerobic CH_4_ oxidation under extreme oxygen limited conditions in which N_2_O production (0.414 μmol.h^−1^.L^−1^) was directly supported by CH_4_ oxidation in *M. denitrificans* strain FJG1T. The latter N_2_O production rate is about 60-fold lower compared to our results obtained under anoxic condition in the absence of CH_4_.

In this study, the transcriptome data showed that the *pmoCAB*1 and *pmoCAB*2 operons were tightly regulated by oxygen as observed previously (Khadem et al., 2012a). Recently, the down-regulation of *pmoCAB* gene was detected in response to 30 mM ${\text{NH}}_{4}^{+}$ concentration in the medium compared to 10 mM ${\text{NO}}_{3}^{-}$ in *Methylocystis* sp. strain SC2 (Dam et al., 2014). It has been shown that CH_4_ oxidation in *Methylocystis* sp. strain SC2 cells supplied with 30 mM ${\text{NH}}_{4}^{+}$ was inhibited at CH_4_ concentrations <400 ppm (v/v; Dam et al., 2014). Our results in all cases showed no expression of the *pmoCAB*3 operon, suggesting other growth conditions could be examined to elucidate the regulation and role of this *pmo* operon. Recently, the concurrent growth of the methanotroph *Methylocella silvestris* was described on CH_4_ and propane (Crombie and Murrell, 2014). Two soluble di-iron center monooxygenase gene clusters (sMMO) were identified with different expression during bacterial growth on these alkanes, although both gene sets were essential for efficient propane utilization (Crombie and Murrell, 2014).

In our study, the *haoAB* genes encoding hydroxylamine dehydrogenase (HAO) and an associated protein were constitutively expressed in cells grown in the H_2_/${\text{NH}}_{4}^{+}$ continuous and batch cultures (Table 3). In *M. capsulatus* Bath the *haoAB* genes were shown to respond to addition of 5 mM of ${\text{NH}}_{4}^{+}$ (Poret-Peterson et al., 2008). The currently accepted model for oxidation of NH_3_ to ${\text{NO}}_{2}^{-}$ proceeds via the intermediate NH_2_OH which in a follow up reaction catalyzed by HAO is oxidized to ${\text{NO}}_{2}^{-}$. Recently, evidence was provided that HAO oxidizes NH_2_OH by only three electrons to NO under both aerobic and anaerobic conditions using purified *Nitrosomonas europaea* HAO (Caranto and Lancaster, 2017). This also implies the need for an enzyme converting NO to ${\text{NO}}_{2}^{-}$. For future research we aim at purifying the HAO from strain SolV to test its properties.

The assimilatory nitrite and nitrate reductase genes were found 9- to 45-fold up-regulated in the CH_4_/${\text{NO}}_{3}^{-}$ continuous culture compared to the cells at μ_max_. These observations are similar to the down-regulation of assimilatory nitrite and nitrate reductase genes in *Methylocystis* sp. strain SC2 under 30 mM ${\text{NH}}_{4}^{+}$ compared to 10 mM nitrate or ${\text{NH}}_{4}^{+}$ (Dam et al., 2014). It has been proposed that methanotrophs with denitrifying capacity might surpass other methanotrophs in ecosystems with high concentrations of nitrogen, because they have the ability to deal with reactive N-compounds (Nyerges et al., 2010). The ${\text{NO}}_{2}^{-}$ reducing capacity of strain SolV helps this microorganism to balance assimilation and tolerance in response to reactive-N molecules in the extreme conditions of its habitat. Our experiments show that strain SolV is well adapted to cope with the fluctuating conditions (presence of H_2_, differences in ${\text{NH}}_{4}^{+}$ and O_2_ concentrations and pH) that may occur in its natural environment.

## Author contributions

SM, AP, MJ, and HO designed the project and experiments. Experimental work was performed by SM, TvA, and AP. SM and AP maintained the chemostat cultures. SM, TvA, AP, MJ, and HO performed data analysis and data interpretation. SM and HO wrote the manuscript with input from AP, TvA, and MJ. HO and MJ supervised the research.

### Conflict of interest statement

The authors declare that the research was conducted in the absence of any commercial or financial relationships that could be construed as a potential conflict of interest.
