# Supplementary material for: Ammonia Oxidation and Nitrite Reduction in the Verrucomicrobial Methanotroph Methylacidiphilum fumariolicum SolV
Source: Front Microbiol. 2017 Sep 27;8:1901. doi: 10.3389/fmicb.2017.01901 (PMC5623727; doi:10.3389/fmicb.2017.01901)
Supplement: Supplementary file 1 [file Image1.PDF]

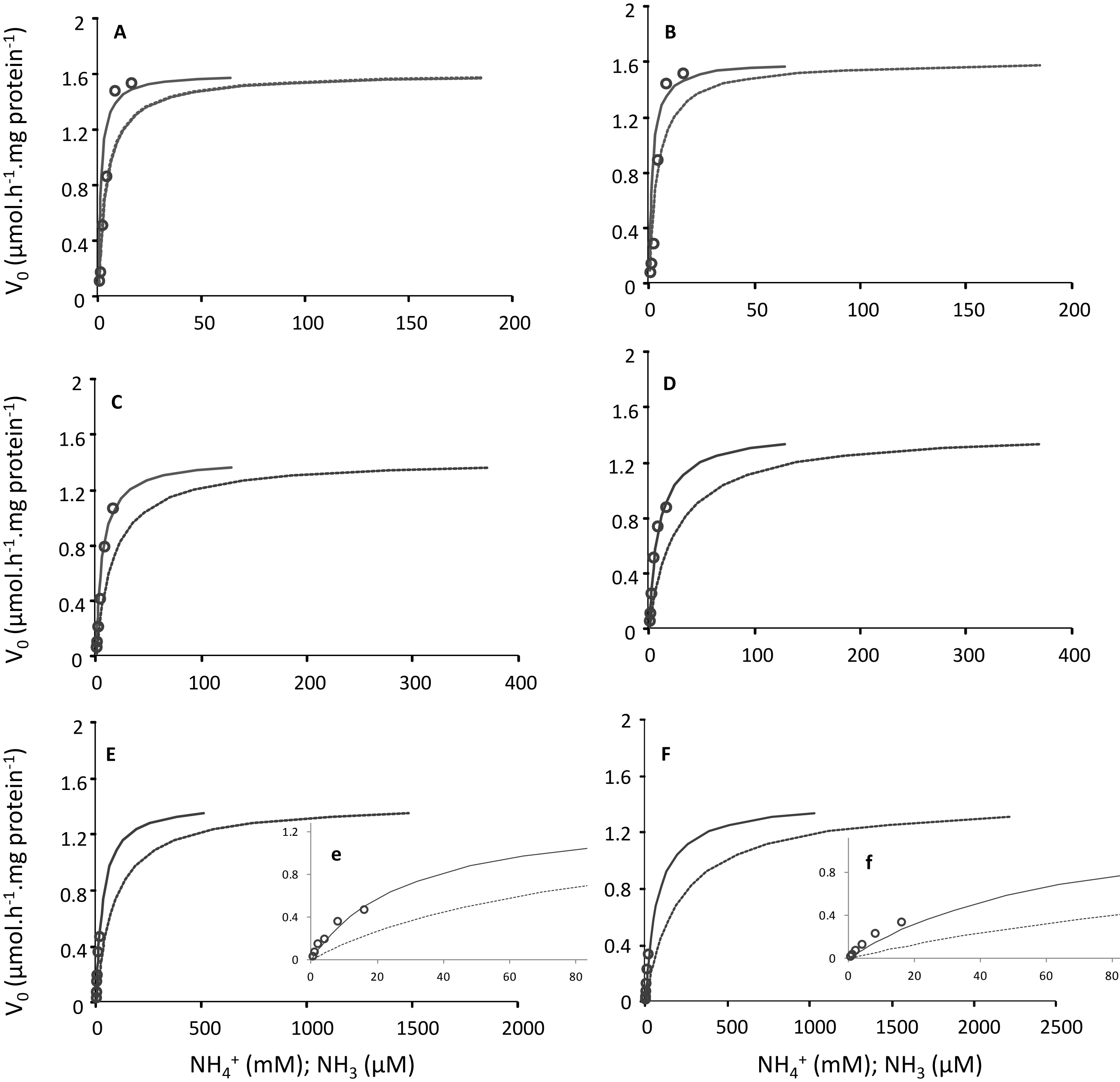

Supplementary Figure S1. Kinetics of nitrite production from ammonia with variable CH_4 supply at pH 6. NO_2^- production from NH_4^+ in the presence of 0.5 % (A), 1 % (B), 2 % (C), 3 % (D), 4 % (E and e) and 8 % CH_4 (F and f). Based on the experimental data points (open circles), the best fitting curve to Michaelis–Menten kinetics for NH_4^+ (solid line) and NH_3 (dashed line) was determined. Each data point represents the average of two replicates.
