# Supplementary material for: Ammonia Oxidation and Nitrite Reduction in the Verrucomicrobial Methanotroph Methylacidiphilum fumariolicum SolV
Source: Front Microbiol. 2017 Sep 27;8:1901. doi: 10.3389/fmicb.2017.01901 (PMC5623727; doi:10.3389/fmicb.2017.01901)
Supplement: Supplementary file 2 [file Image2.PDF]

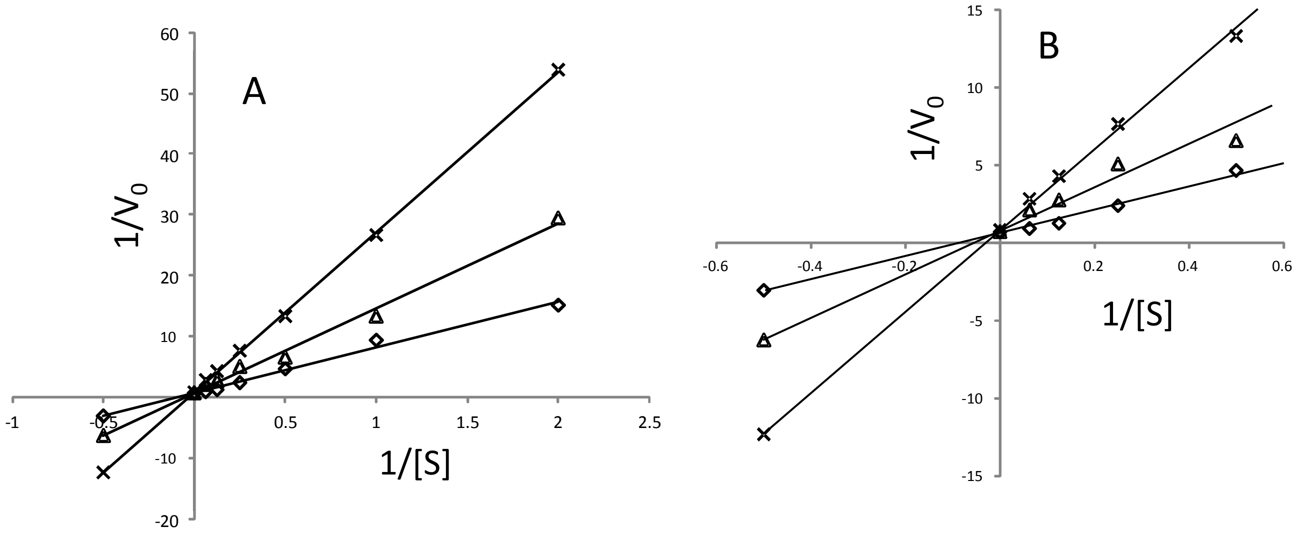

Supplementary Figure S2. The Lineweaver–Burk plot of the NO_2^- production from NH_3 based on the experimental data points from Supplementary Figure S1 with 2 % (open diamonds), 4 % (open triangles) and 8 % (solid crosses) CH_4 at pH 6. Figure B is an enlarged view of the center of figure A.
