# Supplementary material for: Ammonia Oxidation and Nitrite Reduction in the Verrucomicrobial Methanotroph Methylacidiphilum fumariolicum SolV
Source: Front Microbiol. 2017 Sep 27;8:1901. doi: 10.3389/fmicb.2017.01901 (PMC5623727; doi:10.3389/fmicb.2017.01901)
Supplement: Supplementary file 3 [file Image3.PDF]

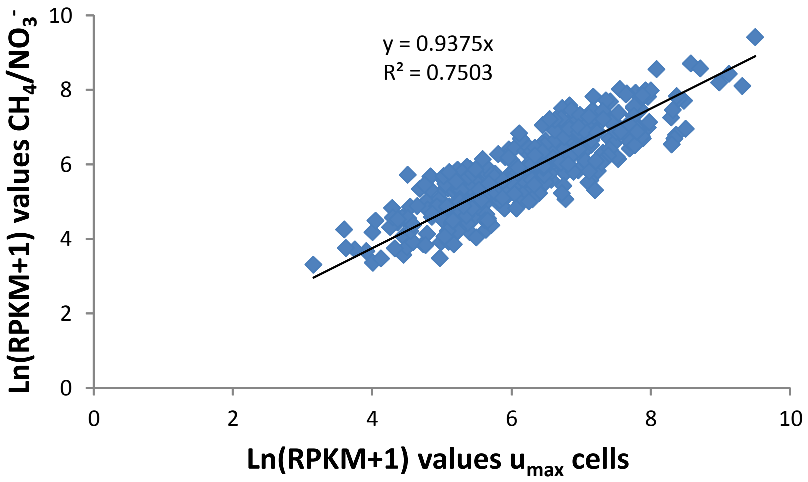

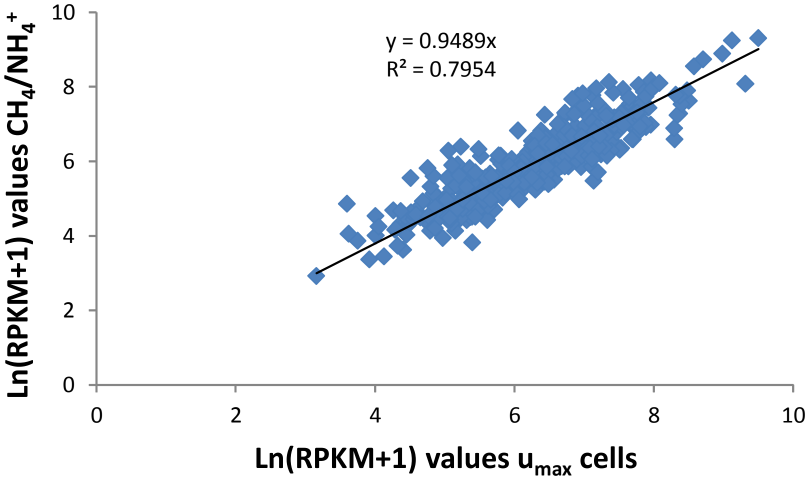

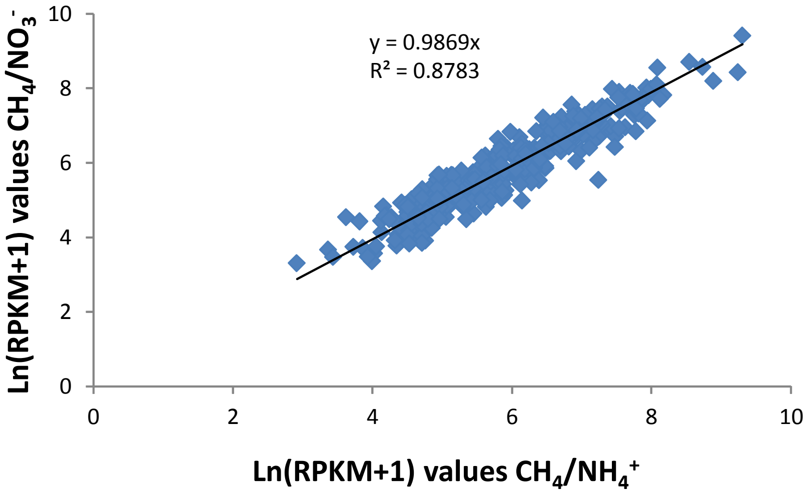

Supplementary Figure S3. Plots of Ln(RPKM + 1) values of 384 housekeeping genes (in total 427.9 kbp) involved in energy generation, ribosome assembly, carbon fixation (CBB cycle), C1 metabolism (except for *pmo*), amino acid synthesis, cell wall synthesis, translation, transcription, DNA replication, and tRNA synthesis.
