# Supplementary material for: Ammonia Oxidation and Nitrite Reduction in the Verrucomicrobial Methanotroph Methylacidiphilum fumariolicum SolV
Source: Front Microbiol. 2017 Sep 27;8:1901. doi: 10.3389/fmicb.2017.01901 (PMC5623727; doi:10.3389/fmicb.2017.01901)
Supplement: Supplementary file 4 [file Table1.PDF]

Supplementary Table S1 | RNA-Seq analysis of *Methylacidiphilum fumariolicum* SolV grown under different conditions

				H_2/NH_4^+ bioreactor		CH_4/NO_3^- bioreactor		Cells at μMax			* = housekeeping genes used to test robustness of the transcriptome data
**Name**	**Gene length**	**Gene**		**RPKM**	**Unique gene reads**	**RPKM**	**Unique gene reads**	**RPKM**	**Unique gene reads**		**Protein annotation**
Mfumv2_0001	1356	dnaA	*	260	382	356	162	222	720	*	Chromosomal replication initiator protein DnaA
Mfumv2_0002	1116	dnaN	*	308	377	299	149	620	2312	*	DNA polymerase III subunit beta
Mfumv2_0003	387			163	77	144	19	349	397		conserved protein of unknown function
Mfumv2_0005	552	lspA		125	65	28	6	98	125		Lipoprotein signal peptidase
Mfumv2_0006	927	nadA		332	373	189	78	397	900		Quinolinate synthase A
Mfumv2_0007	609			144	107	151	36	358	661		conserved protein of unknown function
Mfumv2_0008	1101		*	80	102	66	31	132	383	*	ATP-dependent DNA ligase
Mfumv2_0009	1290	tuaD		199	251	135	65	445	1809		UDP-glucose 6-dehydrogenase TuaD
Mfumv2_0010	1269	purA	*	298	431	235	156	443	1682	*	Adenylosuccinate synthetase
Mfumv2_0011	795	ispU		413	355	235	102	306	729		undecaprenyl pyrophosphate synthase
Mfumv2_0012	894	cdsA		88	96	59	33	232	676		Phosphatidate cytidylyltransferase
Mfumv2_0013	648	psd		264	165	164	42	281	572		Phosphatidylserine decarboxylase proenzyme
Mfumv2_0014	918	pssA		433	423	363	122	287	788		Phosphatidylserine synthase
Mfumv2_0015	1134	kamA	*	494	592	602	279	913	2609	*	L-lysine 2,3-aminomutase
Mfumv2_0016	1194			368	502	468	223	399	1011		SAM-dependent methyltransferase
Mfumv2_0017	2439	pps		458	1273	216	222	400	2950		phosphoenolpyruvate synthase
Mfumv2_0018	2742			180	639	144	174	254	1995		Acyl-CoA synthetase (ADP forming) alpha and beta chain
Mfumv2_0019	879	spo0J		1176	1191	2022	687	821	2055		Stage 0 sporulation protein J
Mfumv2_0020	267			18	4	0	0	74	65		protein of unknown function
Mfumv2_0021	1803	mutL	*	183	327	210	134	189	900	*	DNA mismatch repair protein MutL
Mfumv2_0022	1101	ychF	*	462	484	314	157	248	730	*	Ribosome-binding ATPase YchF
Mfumv2_0023	552	nusG		68	42	23	10	175	324		Transcriptional activator RfaH, NusG family
Mfumv2_0024	906	rfbD		392	404	317	108	501	1244		dTDP-4-dehydrorhamnose reductase
Mfumv2_0025	1242	degT	*	233	375	269	130	329	954	*	Pleiotropic regulatory protein
Mfumv2_0026	2463	imp		238	673	195	237	272	1879		Organic solvent tolerance protein OstA
Mfumv2_0027	594			159	75	148	37	852	1515		conserved protein of unknown function
Mfumv2_0028	585	kdpC		51	36	79	26	112	206		potassium translocating ATPase, subunit C
Mfumv2_0029	2049	kdpB		65	167	116	129	133	879		potassium translocating ATPase, subunit B
Mfumv2_0030	1719	kdpA		62	111	75	70	93	462		potassium translocating ATPase, subunit A
Mfumv2_0031	2295	priA		60	148	66	55	98	626		Primosomal protein N', superfamily II helicase
Mfumv2_0032	210			2235	442	1088	98	809	557		conserved protein of unknown function
Mfumv2_0033	483	guaD		284	119	251	61	272	394		Guanine deaminase
Mfumv2_0034	159			23	3	8	1	51	21		conserved protein of unknown function
Mfumv2_0035	132			21	2	0	0	11	4		conserved protein of unknown function
Mfumv2_0036	675	norC		429	307	372	119	197	362		Nitric-oxide reductase subunit C
Mfumv2_0037	1422	norB		125	215	84	56	178	848		putative nitric oxide reductase B subunit
Mfumv2_0038	321			34	16	50	10	37	37		conserved membrane protein of unknown function
Mfumv2_0039	519			443	250	323	85	864	1625		conserved protein of unknown function
Mfumv2_0040	141			689	89	411	30	258	71		conserved protein of unknown function
Mfumv2_0041	1620			1719	1109	5080	1215	583	571		conserved protein of unknown function
Mfumv2_0042	210			104	19	169	10	58	18		protein of unknown function
Mfumv2_0043	639	sodA		376	278	399	117	1002	2371		Superoxide dismutase
Mfumv2_0044	1704	recN	*	223	443	218	130	265	1054	*	DNA repair protein RecN
Mfumv2_0045	1014	prfB		425	542	452	165	805	2113		Peptide chain release factor 2
Mfumv2_0046	678			78	47	41	14	107	231		conserved exported protein of unknown function
Mfumv2_0047	474	moaC		313	176	540	85	950	1242		Cyclic pyranopterin monophosphate synthase accessory protein
Mfumv2_0048	504	mog		194	117	108	25	747	1292		Molybdopterin adenylyltransferase
Mfumv2_0049	429	hisI	*	444	216	254	45	532	630	*	Phosphoribosyl-AMP cyclohydrolase
Mfumv2_0050	1104			148	189	121	58	186	543		Acyl-CoA reductase, LuxC
Mfumv2_0051	1095			81	93	33	19	96	273		Acyl-protein synthetase, LuxE

Mfumv2_0052	243			664	161	362	41	939	887		Membrane protein CcmA involved in cell shape determination
Mfumv2_0053	765			213	195	159	50	210	490		Zn-finger protein
Mfumv2_0054	153			1799	261	1273	81	433	204		conserved protein of unknown function
Mfumv2_0055	312			150	44	363	35	79	65		conserved protein of unknown function
Mfumv2_0056	216			71	14	62	8	27	14		conserved protein of unknown function
Mfumv2_0057	342			7	2	17	2	11	8		conserved protein of unknown function
Mfumv2_0058	243			0	0	0	0	6	6		conserved protein of unknown function
Mfumv2_0059	120			15	1	0	0	8	4		conserved protein of unknown function
Mfumv2_0060	2496			665	1665	808	768	225	1053		conserved protein of unknown function
Mfumv2_0061	780			489	437	459	119	248	409		conserved protein of unknown function
Mfumv2_0062	768			278	236	373	93	156	305		conserved protein of unknown function
Mfumv2_0063	402			223	122	276	34	108	94		conserved protein of unknown function
Mfumv2_0064	1356			203	321	371	189	128	406		conserved protein of unknown function
Mfumv2_0065	150			42	9	121	5	88	38		conserved protein of unknown function
Mfumv2_0066	369			140	49	108	26	123	98		conserved protein of unknown function
Mfumv2_0067	1620			510	423	971	366	350	368		conserved protein of unknown function
Mfumv2_0068	195			11	2	52	2	22	7		protein of unknown function
Mfumv2_0069	153			6	1	8	1	13	4		conserved protein of unknown function
Mfumv2_0070	894	phuW		48	41	45	12	191	447		Uncharacterized iron-regulated protein
Mfumv2_0071	690			101	68	31	11	229	376		Phosphatase/phosphohexomutase HAD superfamily
Mfumv2_0073	1158			20	25	3	3	47	158		putative Predicted aspartyl protease
Mfumv2_0074	822	folE		1485	1100	1477	412	795	2004		GTP cyclohydrolase FolE2
Mfumv2_0075	1923	thiC		360	730	329	237	747	3639		thiamin (pyrimidine moiety) biosynthesis protein
Mfumv2_0076	2769	sucA		512	1422	461	505	1051	7590		2-oxoglutarate decarboxylase, thiamin-requiring
Mfumv2_0077	1179	sucB		552	706	557	287	907	2654		dihydrolipoyltranssuccinase
Mfumv2_0078	1401	lpd		196	343	208	133	462	1493		Dihydrolipoyl dehydrogenase 3
Mfumv2_0079	1170	alr	*	121	165	99	60	256	770	*	Alanine racemase
Mfumv2_0080	576			68	49	11	5	187	373		Glycoprotease family enzyme
Mfumv2_0081	426			87	36	51	11	128	145		ATP/GTP binding protein
Mfumv2_0082	948	thiL		119	118	112	38	177	395		Thiamine monophosphate kinase
Mfumv2_0083	780			65	55	74	20	80	183		Predicted metal-dependent membrane protease
Mfumv2_0084	993	sppA		463	494	719	235	471	1307		Periplasmic serine protease, ClpP class
Mfumv2_0085	1530	metG	*	179	321	162	111	274	1118	*	Methionyl-tRNA synthetase
Mfumv2_0086	339	himA		1431	441	945	127	2297	2791		Bacterial nucleoid DNA-binding protein
Mfumv2_0087	1218	hisS	*	323	441	361	164	720	2246	*	Histidine--tRNA ligase
Mfumv2_0088	1824	aspS	*	310	643	448	371	730	4066	*	Aspartate--tRNA ligase
Mfumv2_0089	1431	pykF	*	276	472	490	290	578	2347	*	Pyruvate kinase
Mfumv2_0090	852	dltE		85	82	123	32	205	427		Short-chain dehydrogenase
Mfumv2_0091	744			889	705	722	267	1814	3986		Alpha/beta superfamily hydrolase
Mfumv2_0092	570	dcd		379	259	370	92	643	746		Deoxycytidine triphosphate deaminase
Mfumv2_0093	1089	rlmN		427	484	282	162	795	2463		putative dual-specificity RNA methyltransferase RlmN
Mfumv2_0094	345			432	188	287	48	431	340		conserved protein of unknown function
Mfumv2_0095	1632			1502	1481	1696	743	859	2164		conserved exported protein of unknown function
Mfumv2_0096	1431	nfeD_1		55	82	81	33	49	196		Membrane-bound serine protease, NfeD family
Mfumv2_0097	144			6	1	0	0	2	1		conserved protein of unknown function
Mfumv2_0098	231			30	6	25	2	13	6		protein of unknown function
Mfumv2_0099	363			19	5	41	2	17	11		conserved protein of unknown function
Mfumv2_0100	126			9	1	0	0	4	2		conserved protein of unknown function
Mfumv2_0101	471	nfeD_2		76	42	49	9	113	200		Membrane-bound serine protease, NfeD family
Mfumv2_0102	1047			381	377	267	99	128	414		conserved protein of unknown function
Mfumv2_0104	534			209	116	237	36	229	358		conserved protein of unknown function
Mfumv2_0105	1416	cusB		94	130	70	40	129	482		Copper efflux pump membrane fusion protein
Mfumv2_0106	3210	cusA		128	423	109	136	120	1257		copper/silver efflux system, membrane component
Mfumv2_0107	408			48	22	43	8	14	17		conserved protein of unknown function
Mfumv2_0109	309			1763	539	748	132	1960	2239		conserved protein of unknown function
Mfumv2_0110	144			17	5	9	1	68	24		protein of unknown function

Mfumv2_0111	702	tolQ		1651	1134	1404	401	1692	3529		Biopolymer transport protein
Mfumv2_0112	417	exbD		682	323	1208	137	1228	1661		Biopolymer transport protein
Mfumv2_0113	285			539	176	463	36	631	364		conserved protein of unknown function
Mfumv2_0114	366	cdc	*	186	62	143	20	64	66	*	ATP-dependent DNA ligase
Mfumv2_0115	579			711	418	891	171	281	488		NUDIX family hydrolase
Mfumv2_0116	357			104	41	155	19	255	269		Large-conductance mechanosensitive channel
Mfumv2_0117	621			137	102	55	20	191	340		conserved protein of unknown function
Mfumv2_0118	759	suhB		157	132	92	38	195	381		Inositol-1-monophosphatase
Mfumv2_0120	1551	arnT		63	115	46	37	121	472		4-amino-4-deoxy-L-arabinose transferase or related glycosyltransferase of PMT family
Mfumv2_0121	654	smtA		280	171	185	60	290	532		SAM-dependent methyltransferase
Mfumv2_0122	717	purQ		229	197	265	72	352	544		Phosphoribosylformylglycinamidine synthase 1
Mfumv2_0123	255	purS		610	156	559	77	389	225		Phosphoribosylformylglycinamidine (FGAM) synthase, PurS component
Mfumv2_0124	1332	purB	*	377	578	599	282	388	1351	*	Adenylosuccinate lyase
Mfumv2_0125	600	ligT		35	23	6	3	51	93		2'-5' RNA ligase
Mfumv2_0126	123			14	3	0	0	54	19		conserved protein of unknown function
Mfumv2_0127	414	nuoA		379	151	244	63	474	650		NADH-quinone oxidoreductase subunit A
Mfumv2_0128	1770	ilvB		940	1708	574	492	751	3700		Acetolactate synthase large subunit
Mfumv2_0129	348		*	365	134	536	71	1603	2071	*	Phenylpyruvate tautomerase family protein
Mfumv2_0130	432	rplM	*	1454	622	890	179	4295	5418	*	50S ribosomal subunit protein L13
Mfumv2_0131	405	rpsI	*	2043	853	1042	171	4914	6040	*	30S ribosomal subunit protein S9
Mfumv2_0132	1038	argC	*	238	296	263	135	1256	3343	*	N-acetyl-gamma-glutamyl-phosphate reductase
Mfumv2_0133	1215	argJ	*	439	748	496	310	953	2831	*	Arginine biosynthesis bifunctional protein ArgJ [Includes: Glutamate N-acetyltransferase]
Mfumv2_0134	891	argB	*	355	411	650	245	795	1788	*	Acetylglutamate kinase
Mfumv2_0135	1224	argD		145	238	273	158	357	1216		Acetylornithine aminotransferase
Mfumv2_0136	918	arcB		161	174	239	91	278	702		Ornithine carbamoyltransferase
Mfumv2_0137	129			0	0	0	0	10	3		conserved protein of unknown function
Mfumv2_0138	1266			190	242	122	64	116	421		putative NADH dehydrogenase, FAD-containing subunit
Mfumv2_0139	717			144	98	63	21	164	334		Kynurenine formamidase
Mfumv2_0139a	528			649	409	401	90	160	245		Small cytochrome C like protein, 1 CxxCH motif
Mfumv2_0140	1665	glgA	*	222	432	195	160	256	1268	*	Glycogen synthase 1
Mfumv2_0141	615			207	156	284	51	298	604		conserved protein of unknown function
Mfumv2_0142	1041	des		802	868	785	460	594	2056		Fatty acid desaturase
Mfumv2_0143	795	thyA		228	186	312	86	282	724		thymidylate synthetase
Mfumv2_0144	2514	mutS	*	95	257	151	106	120	769	*	DNA mismatch repair protein MutS
Mfumv2_0145	372			364	132	448	60	208	227		conserved membrane protein of unknown function
Mfumv2_0146	2607	clpB		1162	3515	2163	1782	931	6509		protein disaggregation chaperone
Mfumv2_0147	378			702	256	586	112	512	427		conserved protein of unknown function
Mfumv2_0148	1014	gppA	*	262	280	189	89	504	1492	*	Exopolyphosphatase
Mfumv2_0149	1389	gadB		199	295	122	61	344	1182		Glutamate decarboxylase
Mfumv2_0150	1677	nfnB		47	77	49	26	115	608		Nitroreductase
Mfumv2_0151	912			256	238	374	113	597	1256		conserved exported protein of unknown function
Mfumv2_0152	171			0	0	0	0	0	0		protein of unknown function
Mfumv2_0153	201			0	0	0	0	5	2		conserved protein of unknown function
Mfumv2_0155	225			78	14	52	4	34	19		conserved protein of unknown function
Mfumv2_0156	771			46	34	71	17	16	24		putative Uncharacterized ABC transporter ATP-binding protein HI_1272
Mfumv2_0157	1029			33	37	29	10	32	61		Transport system permease protein
Mfumv2_0158	1038			34	36	37	18	27	50		Luciferase-like, subgroup
Mfumv2_0159	1140			13	14	7	4	8	19		putative Periplasmic binding protein
Mfumv2_0161	2865			33	80	34	32	21	174		Outer membrane receptor protein, mostly Fe transport
Mfumv2_0162	240			22	0	270	0	25	0		protein of unknown function
Mfumv2_0163	504			39	22	36	5	23	28		conserved membrane protein of unknown function
Mfumv2_0165	126			9	1	0	0	0	0		conserved protein of unknown function
Mfumv2_0166	198			164	33	168	11	75	41		conserved protein of unknown function
Mfumv2_0167	678	mltE		329	222	342	64	268	551		Soluble lytic murein transglycosylase
Mfumv2_0168	891	rbn		52	44	35	12	60	158		Ribonuclease BN family enzyme
Mfumv2_0169	474			2820	1174	1466	300	769	1249		conserved exported protein of unknown function

Mfumv2_0170	2478	cirA		218	520	25	35	235	2225		Outer membrane receptor protein, mostly Fe transport
Mfumv2_0171	423			23	11	17	3	38	56		conserved exported protein of unknown function
Mfumv2_0172	1326	thdF	*	116	169	49	34	147	595	*	tRNA modification GTPase MnmE
Mfumv2_0173	1029	pyrD		188	213	108	47	189	524		Dihydroorotate dehydrogenase (quinone)
Mfumv2_0174	681			210	143	157	64	360	790		conserved protein of unknown function
Mfumv2_0175	2448	cirA		61	137	51	48	74	550		Outer membrane receptor protein, mostly Fe transport
Mfumv2_0176	1095	trmA		32	41	50	16	65	220		SAM-dependent RNA methyltransferase, TrmA family
Mfumv2_0177	612			146	81	133	25	91	150		putative Lead, cadmium, zinc and mercury transporting ATPase
Mfumv2_0178	225			18	4	60	3	9	6		conserved protein of unknown function
Mfumv2_0179	276			16	4	5	1	12	5		conserved protein of unknown function
Mfumv2_0180	510			40	20	46	9	12	15		conserved protein of unknown function
Mfumv2_0181	126			0	0	0	0	0	0		conserved protein of unknown function
Mfumv2_0182	210			8	2	0	0	42	37		conserved protein of unknown function
Mfumv2_0183	270			127	31	116	12	188	144		conserved protein of unknown function
Mfumv2_0184	1125			169	202	161	60	68	146		High-affinity nickel-transporter
Mfumv2_0185	201			34	8	50	2	36	13		conserved protein of unknown function
Mfumv2_0186	1740			160	294	75	67	93	477		Oxidoreductase, molybdopterin-binding
Mfumv2_0187	411			194	88	103	17	80	107		conserved protein of unknown function
Mfumv2_0188	576	ymdB		257	130	124	30	156	235		conserved hypothetical protein
Mfumv2_0189	132			86	11	54	3	116	53		protein of unknown function
Mfumv2_0190	696	smtA		277	191	132	45	260	639		SAM-dependent methyltransferase
Mfumv2_0191	171			17	3	7	1	6	2		conserved protein of unknown function
Mfumv2_0192	201			104	19	97	6	25	8		conserved protein of unknown function
Mfumv2_0193	306	hupB		5940	1906	5202	568	9791	11016		DNA-binding protein HU-beta
Mfumv2_0194	1326	pcnB	*	266	365	241	132	220	749	*	tRNA nucleotidyltransferase/poly(A) polymerase
Mfumv2_0195	1008			35	42	73	27	84	250		Uncharacterized conserved membrane protein
Mfumv2_0196	2835	gcvP	*	150	502	190	260	299	1850	*	glycine decarboxylase, PLP-dependent, subunit (protein P) of glycine cleavage complex
Mfumv2_0197	390	gcvH	*	692	269	547	109	517	533	*	glycine cleavage complex lipoylprotein
Mfumv2_0198	1122	gcvT		401	551	543	225	252	684		Aminomethyltransferase
Mfumv2_0199	579			1282	797	507	94	876	1168		conserved protein of unknown function
Mfumv2_0200	1599			791	1363	918	632	522	1523		putative type I restriction enzyme HindVIIP M protein
Mfumv2_0202	1305			260	379	296	171	194	468		conserved protein of unknown function
Mfumv2_0203	3171			273	986	460	566	207	1098		putative type I restriction enzyme HindVIIP R protein
Mfumv2_0204	957			796	393	1000	164	370	471		Mrr restriction system protein
Mfumv2_0205	327			277	82	167	25	114	66		protein of unknown function
Mfumv2_0206	1017			63	47	258	32	48	51		Mrr restriction system protein
Mfumv2_0207	315			396	142	1077	104	280	243		conserved protein of unknown function
Mfumv2_0208	177			40	9	0	0	17	6		conserved protein of unknown function
Mfumv2_0209	135			1359	173	463	27	3692	1953		conserved protein of unknown function
Mfumv2_0210	279			1774	470	877	104	2946	2517		conserved protein of unknown function
Mfumv2_0211	1764			464	1004	491	320	938	3434		conserved protein of unknown function
Mfumv2_0212	165			7	1	27	1	64	29		conserved protein of unknown function
Mfumv2_0213	198			14	5	6	1	17	12		conserved protein of unknown function
Mfumv2_0214	1707			817	1067	2119	904	320	1037		transposase
Mfumv2_0215	219			35	11	31	2	73	43		conserved protein of unknown function
Mfumv2_0216	678	phoU		165	97	48	12	171	330		Phosphate-specific transport system accessory protein PhoU
Mfumv2_0217	552	rimL		368	185	243	72	147	204		Acetyltransferase, RimL family
Mfumv2_0218	138			24	3	9	1	11	2		conserved exported protein of unknown function
Mfumv2_0219	1173	hcaD		729	894	475	288	785	2520		NAD(FAD)-dependent dehydrogenase
Mfumv2_0220	243			3593	811	2825	327	710	370		conserved protein of unknown function
Mfumv2_0221	414			5154	1827	3516	762	1361	1603		Peroxiredoxin family protein
Mfumv2_0222	408	uspA		744	295	826	152	735	1089		Universal stress protein
Mfumv2_0223	1335			27	37	8	6	87	333		Membrane fusion component of tripartite multidrug resistance system
Mfumv2_0224	1515	proP		134	225	200	99	183	847		Inner membrane component of tripartite multidrug resistance system
Mfumv2_0226	1776	tolC		27	43	9	6	37	189		Outer membrane protein
Mfumv2_0227	531			37	26	68	9	73	123		Predicted small integral membrane protein

Mfumv2_0228	990	ribF		72	68	41	23	277	605		FAD synthase
Mfumv2_0229	714	truB		118	93	132	45	363	677		tRNA pseudouridine synthase B
Mfumv2_0230	984		*	231	222	150	82	517	1383	*	Exopolyphosphatase-related protein
Mfumv2_0231	165			126	21	78	5	101	42		conserved exported protein of unknown function
Mfumv2_0232	1929	manB		304	646	455	372	395	2063		Phosphomannomutase
Mfumv2_0233	1659	putP		63	116	70	48	94	383		Na+/proline symporter
Mfumv2_0234	918		*	668	566	455	185	229	597	*	Fructose-bisphosphate aldolase/6-deoxy-5-ketofructose 1-phosphate synthase
Mfumv2_0235	777	lpxA		161	156	174	61	221	408		UDP-N-acetylglucosamine acetyltransferase
Mfumv2_0236	1305	lpxC		711	947	648	355	619	2194		UDP-3-O-acyl-N-acetylglucosamine deacetylase and (3R)-hydroxymyristoyl-(Acyl carrier protein) dehydratase
Mfumv2_0239	1878	ddpA		61	130	59	41	173	898		ABC-type dipeptide transport system, periplasmic component
Mfumv2_0240	504	ada		164	83	99	24	137	208		Methylated DNA-protein cysteine methyltransferase
Mfumv2_0241	915			31	39	37	9	100	264		SAM-dependent methyltransferase
Mfumv2_0242	807			150	132	122	51	207	420		fragment of putative oligopeptide transport protein (ABC superfamily, atp_bind) (part 2)
Mfumv2_0243	843			130	118	133	44	245	552		fragment of putative oligopeptide transport protein (ABC superfamily, atp_bind) (part 1)
Mfumv2_0244	1068	dppC		100	130	56	29	211	665		ABC-type dipeptide/oligopeptide/nickel transport system, permease component
Mfumv2_0245	969	appB		80	85	44	19	124	333		Oligopeptide transport system permease protein AppB
Mfumv2_0246	1365			84	121	98	54	223	907		conserved protein of unknown function
Mfumv2_0247	948			403	197	526	80	203	303		protein of unknown function
Mfumv2_0248	951			364	161	565	116	203	126		protein of unknown function
Mfumv2_0249	951			232	208	227	93	181	259		protein of unknown function
Mfumv2_0250	267	rpsO	*	3118	807	2753	260	2399	1440	*	30S ribosomal subunit protein S15
Mfumv2_0251	2130	pnp		1555	3993	1769	1514	2337	12488		polynucleotide phosphorylase/polyadenylase
Mfumv2_0252	486	bcp		472	293	639	108	982	1077		Peroxiredoxin
Mfumv2_0253	1092	pfkA		211	293	191	89	455	1297		6-phosphofructokinase
Mfumv2_0254	549			370	210	392	87	765	1412		conserved membrane protein of unknown function
Mfumv2_0255	1236	ispH		910	1373	1145	498	1140	3827		4-hydroxy-3-methylbut-2-enyl diphosphate reductase
Mfumv2_0256	1440	pepA	*	666	1161	698	406	957	4312	*	putative cytosol aminopeptidase
Mfumv2_0257	1359			331	537	508	243	241	849		Beta-propeller repeats containing protein
Mfumv2_0258	828			1767	1405	1193	483	1789	3986		Predicted ATPase
Mfumv2_0259	717			971	819	687	239	1434	2248		conserved protein of unknown function
Mfumv2_0260	465			535	244	449	82	344	531		putative HTH-type transcriptional regulator rrf2-like
Mfumv2_0261	831			33	31	14	6	55	147		conserved exported protein of unknown function
Mfumv2_0262	741			135	112	88	24	151	280		conserved protein of unknown function
Mfumv2_0263	933	lysR		317	270	159	67	295	723		Transcriptional regulator, LysR/CysB family
Mfumv2_0264	1980	speA	*	314	687	258	239	716	4077	*	Biosynthetic arginine decarboxylase
Mfumv2_0266	501			1223	478	473	105	754	1246		conserved protein of unknown function
Mfumv2_0267	738	terC		111	79	70	21	275	455		Membrane protein TerC, possibly involved in tellurium resistance
Mfumv2_0268	222			129	28	0	0	149	102		Membrane protein TerC, possibly involved in tellurium resistance (fragment)
Mfumv2_0269	777	panC	*	105	80	30	13	165	369	*	Pantothenate synthetase
Mfumv2_0270	1032	amiC	*	478	554	391	182	702	2280	*	N-acetylmuramoyl-L-alanine amidase
Mfumv2_0271	1170	ntrB		225	282	180	104	291	914		Signal transduction histidine kinase with PAS domain
Mfumv2_0272	1386	atoC		264	422	387	215	293	974		Sigma54-type response regulator
Mfumv2_0273	1602			38	56	45	24	45	176		conserved exported protein of unknown function
Mfumv2_0274	1590			198	187	1291	323	494	1135		conserved exported protein of unknown function
Mfumv2_0275	1641			82	135	225	98	194	760		conserved exported protein of unknown function
Mfumv2_0276	1221	lysC	*	1408	1770	822	531	931	2870	*	Aspartokinase
Mfumv2_0277	594	nrfG		329	178	298	86	475	742		TPR repeats containing protein
Mfumv2_0280	675			223	165	162	46	120	193		conserved protein of unknown function
Mfumv2_0281	129			27	2	53	2	19	8		conserved protein of unknown function
Mfumv2_0282	2202	fhaB		98	222	241	184	78	466		Large exoprotein involved in heme utilization or adhesion
Mfumv2_0283	135			3	1	0	0	25	10		conserved protein of unknown function
Mfumv2_0284	309			8	3	19	2	6	4		conserved protein of unknown function
Mfumv2_0285	195			60	11	23	1	5	2		conserved protein of unknown function
Mfumv2_0286	3399			99	414	169	217	162	1453		conserved protein of unknown function
Mfumv2_0287	201			789	139	1052	88	125	81		conserved protein of unknown function
Mfumv2_0288	270			42	10	138	9	20	12		conserved protein of unknown function

Mfumv2_0289	144			24	4	40	2	34	16		conserved protein of unknown function
Mfumv2_0290	159			1498	259	1804	112	408	99		conserved protein of unknown function
Mfumv2_0291	144			16	1	77	2	10	2		conserved protein of unknown function
Mfumv2_0292	2520			143	400	197	163	163	1158		conserved protein of unknown function
Mfumv2_0293	1473			86	147	107	57	112	426		Predicted O-methyltransferase (Modular protein)
Mfumv2_0294	1197			104	130	124	46	147	489		Predicted O-methyltransferase (Modular protein)
Mfumv2_0295	1935			165	325	185	126	685	4271		protein of unknown function
Mfumv2_0296	582			170	44	94	11	253	158		conserved protein of unknown function
Mfumv2_0297	2457			120	254	216	150	216	1410		conserved protein of unknown function
Mfumv2_0298	2103			114	274	143	130	167	963		putative glycosyl transferase, family 2
Mfumv2_0299	957	smtA		164	177	271	108	226	536		SAM-dependent methyltransferase
Mfumv2_0300	2484	cirA		54	141	104	123	40	246		Outer membrane receptor protein, mostly Fe transport
Mfumv2_0302	414			72	27	75	15	59	82		conserved exported protein of unknown function
Mfumv2_0303	1038			61	65	64	30	67	215		conserved protein of unknown function
Mfumv2_0304	912	rbsK		111	93	93	36	106	282		Sugar kinase, ribokinase family
Mfumv2_0306	870	nadF		149	134	89	32	269	720		putative inorganic polyphosphate/ATP-NAD kinase
Mfumv2_0307	1083			386	493	541	236	319	899		conserved protein of unknown function
Mfumv2_0308	456	ptsN		129	59	151	18	128	186		Phosphotransferase system mannitol/fructose-specific IIA domain (Ntr-type)
Mfumv2_0309	585			328	232	119	37	389	670		conserved exported protein of unknown function
Mfumv2_0310	1422			146	229	49	38	207	895		conserved membrane protein of unknown function
Mfumv2_0311	3228	czcA		61	219	43	50	55	538		Cation efflux system protein CzcA
Mfumv2_0312	150			251	42	103	7	87	46		conserved protein of unknown function
Mfumv2_0313	147			0	0	0	0	12	3		protein of unknown function
Mfumv2_0314	1302			50	59	23	18	61	261		Permease of the major facilitator superfamily
Mfumv2_0315	1314			84	110	43	28	73	262		Permease of the major facilitator superfamily
Mfumv2_0316	1920	nrfG		199	387	101	103	424	2612		TPR repeats containing protein
Mfumv2_0317	828	trpC	*	629	620	618	208	764	1591	*	Indole-3-glycerol phosphate synthase
Mfumv2_0318	654	trpF	*	321	268	312	80	396	624	*	N-(5'-phosphoribosyl)anthranilate isomerase
Mfumv2_0319	1221	trpB	*	496	692	597	253	640	2122	*	tryptophan synthase, beta subunit
Mfumv2_0320	147			148	20	345	17	119	27		conserved protein of unknown function
Mfumv2_0321	174			191	36	240	11	97	33		protein of unknown function
Mfumv2_0323	624	gmk		369	206	271	75	352	519		Guanylate kinase
Mfumv2_0324	594	dfp		351	207	388	116	306	459		Phosphopantothenoylcysteine synthetase/decarboxylase
Mfumv2_0325	504	purE		252	156	219	55	452	528		N5-carboxyaminoimidazole ribonucleotide mutase
Mfumv2_0326	1155	purK		152	242	124	83	311	986		N5-carboxyaminoimidazole ribonucleotide synthase
Mfumv2_0327	231	thiS		115	28	78	9	115	62		Sulfur transfer protein involved in thiamine biosynthesis
Mfumv2_0328	1140	thiH		237	308	134	85	174	478		Thiamine biosynthesis enzyme ThiH
Mfumv2_0329	2466	rfaG		247	748	133	199	494	3417		Glycosyltransferase
Mfumv2_0330	804			69	76	63	20	177	359		UspA family protein
Mfumv2_0331	2448	lonB		235	632	222	215	396	2747		ATP-dependent protease La Type II
Mfumv2_0332	411			972	342	843	156	403	384		conserved exported protein of unknown function
Mfumv2_0333	1017	lysR		1725	1754	2958	1088	1652	5137		Transcriptional regulator, LysR/CysB family
Mfumv2_0334	1065	splB		597	714	1088	381	398	977		DNA repair photolyase
Mfumv2_0335	555	slp		117	63	32	9	139	228		Starvation-inducible outer membrane lipoprotein
Mfumv2_0336	387			213	77	82	18	203	250		conserved protein of unknown function
Mfumv2_0337	693	tonB		79	63	69	18	284	664		Periplasmic protein TonB
Mfumv2_0338	1014	smtA		154	178	107	49	258	808		SAM-dependent methyltransferase
Mfumv2_0339	999	glk		200	240	259	84	264	548		Glucokinase
Mfumv2_0340	1437			37	52	40	20	38	117		conserved protein of unknown function
Mfumv2_0341	993			519	485	273	146	188	567		putative NADP-dependent oxidoreductase
Mfumv2_0342	1584	ble		64	110	39	27	89	297		Uncharacterized domain involved in trehalose biosynthesis
Mfumv2_0343	1995	glgE		111	261	174	171	316	1663		Alpha-1,4-glucan:maltose-1-phosphate maltosyltransferase
Mfumv2_0344	441			1589	768	936	200	542	668		conserved protein of unknown function
Mfumv2_0345	384			140	55	124	16	460	603		Uncharacterized enzyme of heme biosynthesis
Mfumv2_0346	594			162	108	128	30	1340	3020		conserved protein of unknown function
Mfumv2_0347	996	mauB		99	122	135	65	235	615		Methylamine dehydrogenase heavy chain

Mfumv2_0348	543	mauE		23	19	17	7	58	96		Methylamine utilisation protein MauE
Mfumv2_0349	657	mauD		288	167	311	91	90	132		Methylamine utilization protein MauD
Mfumv2_0350	543	mauA		119	86	450	72	108	112		Methylamine dehydrogenase light chain
Mfumv2_0351	480	mauG	*	41	17	15	3	50	75	*	putative Cytochrome c, class I
Mfumv2_0352	123			0	0	37	1	22	5		conserved protein of unknown function
Mfumv2_0353	1233	rfaG		263	339	277	134	371	1275		Glycosyltransferase
Mfumv2_0354	726	wecD		118	101	168	38	163	334		Acetyltransferase, GNAT family
Mfumv2_0355	750			74	51	80	20	139	309		Predicted deacetylase
Mfumv2_0356	2415	rfaG		112	301	139	129	383	2317		PHP family phosphoesterase fused to glycosyltransferase
Mfumv2_0357	1731			262	537	421	256	424	2069		Oligoendopeptidase F
Mfumv2_0358	348		*	2385	715	936	205	1235	1558	*	Ribosome-associated protein Y (PSrp-1)
Mfumv2_0359	1590			634	1033	523	331	555	2326		Uncharacterized AIPM/Hcit synthase family transferase aq_356
Mfumv2_0360	177			4266	847	5608	386	3162	1726		conserved exported protein of unknown function
Mfumv2_0361	840	lgt		80	70	100	32	159	312		Prolipoprotein diacylglyceryl transferase
Mfumv2_0363	1848	thrS	*	416	864	559	371	647	3352	*	threonyl-tRNA synthetase
Mfumv2_0364	618	infC	*	879	612	984	195	1345	2621	*	Translation initiation factor IF-3
Mfumv2_0365	363	rplT	*	1884	773	2688	313	2117	2221	*	50S ribosomal subunit protein L20
Mfumv2_0366	1155	dxr		648	862	1342	480	852	2226		1-deoxy-D-xylulose 5-phosphate reductoisomerase
Mfumv2_0367	1437			567	871	935	461	555	1966		Predicted membrane-associated Zn-dependent protease
Mfumv2_0368	1755	ispG		729	1547	721	580	807	3969		4-hydroxy-3-methylbut-2-en-1-yl diphosphate synthase
Mfumv2_0369	936			2684	2555	5358	1867	727	1392		conserved protein of unknown function
Mfumv2_0370	360			10	3	16	2	18	21		conserved protein of unknown function
Mfumv2_0371	276	tatA		1141	299	899	94	820	717		Sec-independent protein translocase protein TatA
Mfumv2_0372	1008			316	350	421	140	1301	2711		Radical SAM superfamily enzyme
Mfumv2_0373	375			410	178	497	67	1111	876		conserved protein of unknown function
Mfumv2_0374	642			212	142	256	68	337	437		conserved protein of unknown function
Mfumv2_0375	162			88	16	158	9	42	20		conserved protein of unknown function
Mfumv2_0376	153			70	10	68	3	21	11		protein of unknown function
Mfumv2_0377	657	actA	*	260	202	203	54	360	820	*	Cytochrome c3 alternative oxidase operon
Mfumv2_0378	3138	actB		254	980	250	355	274	2399		Anaerobic dehydrogenase and Fe-S-cluster domain
Mfumv2_0379	1443	actC		174	279	203	123	170	718		Polysulphide reductase
Mfumv2_0380	597	actD		177	119	464	93	219	416		conserved protein of unknown function
Mfumv2_0381	618	actE	*	206	162	227	57	290	512	*	Cytochrome c family protein
Mfumv2_0382	1203	actF		184	283	127	71	182	691		conserved membrane protein of unknown function
Mfumv2_0383	372	actG		237	110	324	37	290	313		conserved protein of unknown function
Mfumv2_0384	1446	ccoN	*	80	138	46	39	113	487	*	Cbb3-type cytochrome oxidase, subunit 1
Mfumv2_0385	597	ccoO	*	251	173	187	56	253	495	*	Cbb3-type cytochrome oxidase, cytochrome c subunit
Mfumv2_0386	639	cccA	*	235	177	182	50	276	491	*	Cytochrome c family protein
Mfumv2_0387	1848	coxN	*	1089	1974	558	625	794	3648	*	Alternative cytochrome c oxidase subunit 1
Mfumv2_0388	792	cyoC	*	1419	1246	838	366	842	1725	*	Heme/copper-type cytochrome oxidase, subunit 3
Mfumv2_0389	297			928	290	512	102	423	310		Predicted small integral membrane protein
Mfumv2_0390	360			873	363	432	87	1130	1312		conserved protein of unknown function
Mfumv2_0391	285			1342	462	1014	95	800	687		conserved protein of unknown function
Mfumv2_0392	792	cyoA	*	1785	1517	822	331	1093	2923	*	Cytochrome c oxidase subunit 2
Mfumv2_0393	450			683	315	255	67	317	343		conserved protein of unknown function
Mfumv2_0394	615			79	63	71	23	101	153		conserved protein of unknown function
Mfumv2_0395	1128			175	203	104	57	209	641		conserved protein of unknown function
Mfumv2_0396	243			13	4	52	4	83	42		conserved protein of unknown function
Mfumv2_0397	420			4051	1828	6394	1043	10977	16274		conserved exported protein of unknown function
Mfumv2_0399	2043			61	151	56	61	155	925		Alpha-amylase/alpha-mannosidase
Mfumv2_0400	2157			149	371	107	121	288	1737		Alpha-amylase/alpha-mannosidase
Mfumv2_0401	1029	galT		122	151	147	65	397	776		Galactose-1-phosphate uridylyltransferase
Mfumv2_0402	132			0	0	0	0	109	60		conserved protein of unknown function
Mfumv2_0403	1143	tgt		48	52	16	12	228	841		tRNA-guanine transglycosylase
Mfumv2_0404	423	yajC		572	275	547	83	1133	1275		Preprotein translocase subunit YajC
Mfumv2_0405	2295	secD		301	818	361	324	535	2989		Preprotein translocase subunit SecD

Mfumv2_0406	741			195	190	189	66	381	730		Predicted periplasmic solute-binding protein
Mfumv2_0407	1110	mqnC		535	696	421	203	759	2476		Cyclic dehypoxanthine futalosine synthase
Mfumv2_0408	3234	carB		347	1395	674	766	514	3980		carbamoyl-phosphate synthase large subunit
Mfumv2_0409	471			165	87	308	59	173	219		DoxX family protein
Mfumv2_0410	1053			61	71	85	40	68	182		Calcineurin-like phosphohydrolase
Mfumv2_0411	2406	cirA		46	114	78	64	47	278		Outer membrane receptor protein, mostly Fe transport
Mfumv2_0412	720	hisA		135	134	190	71	270	512		1-(5-phosphoribosyl)-5-[(5-phosphoribosylamino)methylideneamino] imidazole-4-carboxamide isomerase
Mfumv2_0413	672	hisH		240	163	88	26	519	855		Imidazole glycerol phosphate synthase subunit HisH
Mfumv2_0414	822	dapF	*	654	548	352	132	634	1800	*	Diaminopimelate epimerase
Mfumv2_0415	906	dapA	*	482	489	574	217	534	1158	*	4-hydroxy-tetrahydrodipicolinate synthase
Mfumv2_0416	744	dapB	*	269	253	346	86	444	762	*	4-hydroxy-tetrahydrodipicolinate reductase
Mfumv2_0417	711	smtA		105	91	122	34	192	337		SAM-dependent methyltransferase
Mfumv2_0418	150			160	17	73	6	15	5		transposase
Mfumv2_0419	372			76	31	164	25	56	32		conserved protein of unknown function
Mfumv2_0420	531			183	118	797	117	157	187		conserved protein of unknown function
Mfumv2_0421	1650			1167	2165	3272	1734	529	1771		conserved protein of unknown function
Mfumv2_0422	165			90	20	188	10	112	26		conserved protein of unknown function
Mfumv2_0423	213			165	44	336	24	63	26		conserved protein of unknown function
Mfumv2_0424	93			275	32	1149	45	153	36		Pseudogene (CRISPR-repeat is encoded here)
Mfumv2_0425	159			397	61	840	45	311	182		Pseudogene (CRISPR-repeat is encoded here)
Mfumv2_0426	144			1181	164	1375	75	321	148		Pseudogene (CRISPR-repeat is encoded here)
Mfumv2_0427	117			923	100	962	66	286	99		Pseudogene (CRISPR-repeat is encoded here)
Mfumv2_0428	312			1036	350	835	119	1186	1243		Pseudogene (CRISPR-repeat is encoded here)
Mfumv2_0429	378	cas		33	17	30	3	47	40		CRISPR-associated endoribonuclease Cas2
Mfumv2_0430	1008	cas		12	12	9	2	29	79		CRISPR-associated endonuclease Cas1
Mfumv2_0431	1113			135	151	148	96	89	240		conserved exported protein of unknown function
Mfumv2_0432	954			105	122	125	45	170	446		conserved protein of unknown function
Mfumv2_0433	1233			79	116	119	54	110	285		conserved protein of unknown function
Mfumv2_0434	150			146	19	54	3	245	115		conserved protein of unknown function
Mfumv2_0435	480			178	102	350	71	127	143		conserved protein of unknown function
Mfumv2_0436	1422			237	370	235	156	217	851		conserved protein of unknown function
Mfumv2_0437	366			132	48	93	15	146	153		conserved protein of unknown function
Mfumv2_0438	951			170	215	134	55	178	497		conserved protein of unknown function
Mfumv2_0439	1299			249	373	238	144	221	835		conserved protein of unknown function
Mfumv2_0440	2997			176	587	106	163	158	1254		conserved protein of unknown function
Mfumv2_0441	132			27	3	0	0	9	5		protein of unknown function
Mfumv2_0442	1887			103	232	58	54	135	650		putative Adenosine deaminase
Mfumv2_0443	1272			576	819	552	327	466	1567		conserved protein of unknown function
Mfumv2_0444	171			30	5	34	2	17	10		conserved protein of unknown function
Mfumv2_0445	216			26	6	12	2	11	4		conserved protein of unknown function
Mfumv2_0447	1416	wcaJ		48	68	55	33	39	182		Sugar transferase, WcaJ family
Mfumv2_0448	1047			4230	4698	7785	2962	2614	8664		conserved exported protein of unknown function
Mfumv2_0449	1137	wza		569	721	1093	406	381	993		Periplasmic protein involved in polysaccharide export, contains SLBB domain of b-grasp fold
Mfumv2_0450	2322	gumC		191	543	217	258	228	1564		Capsular polysaccharide synthesis enzyme cpsD fused to Mrp family ATPase
Mfumv2_0451	1182			71	103	74	35	50	130		putative Rhs family protein fused to TPR repeats
Mfumv2_0452	1479			73	100	71	43	68	289		putative Lipid A core-O-antigen ligase or related enzyme
Mfumv2_0453	1230	rfaG		240	275	177	94	179	612		Glycosyltransferase
Mfumv2_0454	1437			786	208	2430	296	279	166		conserved protein of unknown function
Mfumv2_0455	219			152	37	791	61	89	33		protein of unknown function
Mfumv2_0456	585			577	89	1003	47	545	67		conserved protein of unknown function
Mfumv2_0457	687	citB		137	105	133	28	307	661		DNA-binding response regulator, NarL family (REC-HTH domains)
Mfumv2_0458	555			424	243	307	72	269	478		putative aromatic acid decarboxylase
Mfumv2_0459	873	ubiA		138	136	143	50	127	305		4-hydroxybenzoate polyprenyltransferase
Mfumv2_0460	1209	mqnE		276	383	310	143	442	1433		Aminodeoxyfutalosine synthase
Mfumv2_0461	2064	ftsH	*	2785	6059	3038	2138	1908	9527	*	ATP-dependent zinc metalloprotease FtsH 2
Mfumv2_0462	270			211	50	340	34	72	30		conserved protein of unknown function

Mfumv2_0463	192			746	112	585	45	145	72		protein of unknown function
Mfumv2_0464	1377			274	219	845	264	187	267		conserved protein of unknown function
Mfumv2_0465	183			19	4	119	7	7	3		protein of unknown function
Mfumv2_0466	492			84	0	368	0	0	0		RNA-directed DNA polymerase (Reverse transcriptase)
Mfumv2_0467	381			157	0	505	0	48	0		conserved protein of unknown function
Mfumv2_0468	138			3	1	33	1	0	0		conserved protein of unknown function
Mfumv2_0469	339			40	11	36	3	17	13		conserved protein of unknown function
Mfumv2_0470	393			36	13	30	4	29	25		Glycosyltransferase
Mfumv2_0472	192			7	2	24	1	34	19		conserved protein of unknown function
Mfumv2_0473	1050			218	215	195	81	131	422		conserved protein of unknown function
Mfumv2_0474	147			745	74	1042	46	1173	97		protein of unknown function
Mfumv2_0475	1707			1312	1378	2292	844	597	1587		transposase
Mfumv2_0476	1038			124	131	69	31	194	629		conserved protein of unknown function
Mfumv2_0477	1392			102	146	55	41	108	421		conserved membrane protein of unknown function
Mfumv2_0478	1212	rfaG		70	101	37	26	63	168		Glycosyltransferase
Mfumv2_0479	1347			122	156	34	27	91	356		conserved membrane protein of unknown function
Mfumv2_0480	999			134	155	176	67	59	164		VPS10 repeats containing protein
Mfumv2_0481	4086			96	448	91	126	107	1366		conserved exported protein of unknown function
Mfumv2_0482	951			21	24	9	4	63	141		Methyltransferase
Mfumv2_0483	858	wcaA		135	128	58	30	139	311		Glycosyltransferase
Mfumv2_0484	1689			507	175	1201	148	388	298		protein of unknown function
Mfumv2_0485	726	smtA		84	51	43	17	80	182		SAM-dependent methyltransferase
Mfumv2_0487	144			207	25	214	9	677	50		protein of unknown function
Mfumv2_0488	1707			987	1200	2721	1048	353	973		transposase
Mfumv2_0489	1620			200	48	664	66	109	56		conserved protein of unknown function
Mfumv2_0490	1023			25	17	54	8	15	17		protein of unknown function
Mfumv2_0491	102			4	1	0	0	10	2		conserved protein of unknown function
Mfumv2_0492	942			54	52	28	12	93	190		conserved protein of unknown function
Mfumv2_0493	1023			247	252	185	75	292	860		putative SAM-dependent methyltransferase
Mfumv2_0495	303			360	116	368	52	199	160		Glycosyltransferase
Mfumv2_0496	234			33	7	0	0	18	9		conserved protein of unknown function
Mfumv2_0497	189			19	2	7	1	113	76		conserved protein of unknown function
Mfumv2_0498	2160			78	188	97	92	230	1386		conserved protein of unknown function
Mfumv2_0499	1032	ccpA	*	741	802	757	350	655	1769	*	Cytochrome c551 peroxidase
Mfumv2_0500	1803			399	776	287	244	252	1234		SpoIVB-like peptidase, S55 family
Mfumv2_0501	1359	hemL		257	376	233	131	258	1006		Glutamate-1-semialdehyde 2,1-aminomutase
Mfumv2_0502	1887	pol	*	417	881	311	247	319	1654	*	DNA polymerase IV fused to PHP phosphatase
Mfumv2_0503	864	folP		161	148	233	66	167	427		Dihydropteroate synthase
Mfumv2_0505	744			127	101	173	47	151	346		conserved membrane protein of unknown function
Mfumv2_0506	213			120	30	241	21	77	49		conserved protein of unknown function
Mfumv2_0507	237			59	22	66	3	143	82		conserved protein of unknown function
Mfumv2_0508	1374	glmM		309	481	266	165	428	1782		phosphoglucosamine mutase
Mfumv2_0509	858			160	171	186	54	272	579		conserved exported protein of unknown function
Mfumv2_0510	579	pyrE		219	112	192	61	173	331		Orotate phosphoribosyltransferase
Mfumv2_0511	144			8	1	31	1	5	1		conserved protein of unknown function
Mfumv2_0512	519			145	96	220	49	80	103		conserved protein of unknown function
Mfumv2_0513	951			378	432	334	131	355	941		conserved protein of unknown function
Mfumv2_0514	1635	mviN		108	183	100	53	79	403		Protein MurJ homolog
Mfumv2_0515	1026	rfaF		100	121	124	45	99	310		ADP-heptose:LPS heptosyltransferase
Mfumv2_0516	1263	lpxK		310	445	424	229	432	1697		Tetraacyldisaccharide 4'-kinase
Mfumv2_0517	861	potD		103	78	67	34	281	706		Spermidine/putrescine-binding periplasmic protein
Mfumv2_0518	492			2585	1328	2782	563	1902	2650		conserved protein of unknown function
Mfumv2_0519	2538	pqqF		408	1347	680	664	718	4294		putative coenzyme PQQ synthesis protein F
Mfumv2_0520	381			270	129	497	75	455	484		conserved protein of unknown function
Mfumv2_0521	1011	moaA		253	327	289	118	537	1742		Cyclic pyranopterin monophosphate synthase
Mfumv2_0522	279			166	55	81	10	341	299		Molybdopterin converting factor, small subunit

Mfumv2_0523	411	moaE		167	96	171	28	293	339		Molybdopterin converting factor, large subunit
Mfumv2_0524	174			325	65	565	34	223	81		protein of unknown function
Mfumv2_0525	1917	cysN		653	1890	582	605	876	3638		Sulfate adenylyltransferase subunit 1 and adenylylsulfate kinase
Mfumv2_0526	804	cysD		757	825	267	91	1057	2075		sulfate adenylyltransferase, subunit 2
Mfumv2_0527	720	cysH		281	293	251	61	225	416		3'-phosphoadenosine 5'-phosphosulfate reductase
Mfumv2_0528	1161	nifV	*	66	94	197	54	149	434	*	Homocitrate synthase 1
Mfumv2_0530	894	nifH		1956	1911	16484	3183	92	198		Nitrogenase iron protein
Mfumv2_0531	1476	nifD		520	850	4562	1552	84	338		Nitrogenase molybdenum-iron protein alpha chain
Mfumv2_0532	1581	nifK		947	1717	5530	1992	104	298		Nitrogenase molybdenum-iron protein beta chain
Mfumv2_0533	1380	nifE		614	920	5845	1953	108	349		Nitrogenase iron-molybdenum cofactor biosynthesis protein NifE
Mfumv2_0534	1344	nifN		387	591	1858	586	88	276		Nitrogenase iron-molybdenum cofactor biosynthesis protein NifN
Mfumv2_0535	399	nifX		212	96	1440	139	81	102		Protein NifX
Mfumv2_0536	471			296	165	3049	339	88	131		conserved protein of unknown function
Mfumv2_0537	192			176	38	850	38	41	12		conserved protein of unknown function
Mfumv2_0538	579	ppa		1209	708	1231	271	3211	6855		Inorganic pyrophosphatase
Mfumv2_0539	420	merR		123	61	232	35	399	394		Mercuric resistance operon regulatory protein
Mfumv2_0540	1653	merA		26	44	38	17	41	167		Mercuric reductase
Mfumv2_0541	1569	nifB		104	172	697	237	26	96		FeMo cofactor biosynthesis protein NifB
Mfumv2_0543	225	frxA		133	34	716	33	95	66		Ferredoxin-like protein in nif region
Mfumv2_0544	342	sufA		274	109	1708	129	64	47		Fe-S cluster assembly scaffold protein SufA
Mfumv2_0545	516			134	78	1114	122	64	99		conserved protein of unknown function
Mfumv2_0546	822			213	202	1247	226	96	207		FeS4 cluster protein and leucine rich repeats
Mfumv2_0547	282			191	61	548	37	132	117		NifZ domain
Mfumv2_0548	1167	iscS		193	260	817	225	123	344		Cysteine desulfurase
Mfumv2_0550	594			56	39	267	35	82	119		NifQ family protein
Mfumv2_0551	858	sseA		287	278	1297	259	233	601		putative thiosulfate sulfurtransferase SseA
Mfumv2_0552	1590	nifA		66	103	264	93	136	570		Nif-specific regulatory protein
Mfumv2_0553	402	yadR		70	32	249	22	27	39		putative chaperone involved in Fe-S cluster assembly and activation; hesB-like
Mfumv2_0555	210	fixU		102	20	488	24	106	78		Protein FixU homolog
Mfumv2_0556	1149	wecE	*	114	146	610	155	97	244	*	Pyridoxal phosphate-dependent enzyme
Mfumv2_0557	225			93	21	194	9	74	52		conserved protein of unknown function
Mfumv2_0558	873			140	135	614	139	84	229		conserved protein of unknown function
Mfumv2_0559	366	nifW		156	65	1083	89	83	74		Nitrogenase-stabilizing/protective protein NifW
Mfumv2_0560	840	fixA		121	110	1128	228	68	146		Protein FixA
Mfumv2_0561	1125	fixB		168	216	1467	419	66	224		Protein FixB
Mfumv2_0562	1299	fixC		235	337	1347	440	95	347		Protein FixC
Mfumv2_0563	285	fixX		493	160	3387	239	148	110		putative 4Fe-4S ferredoxin
Mfumv2_0564	696			2494	1715	2226	842	830	1604		conserved exported protein of unknown function
Mfumv2_0565	1488			315	506	634	297	312	1185		conserved protein of unknown function
Mfumv2_0566	1014			149	187	248	96	263	712		Uncharacterized 37.6 kDa protein in cld 5'region
Mfumv2_0567	363			372	112	92	17	436	607		conserved protein of unknown function
Mfumv2_0568	519			116	65	68	14	160	257		conserved exported protein of unknown function
Mfumv2_0569	471			388	197	337	66	426	622		conserved protein of unknown function
Mfumv2_0570	387			4204	1844	6666	902	2985	2373		transposase (fragment)
Mfumv2_0571	171			68	13	83	6	49	15		conserved protein of unknown function
Mfumv2_0572	420			47	14	129	16	18	11		conserved protein of unknown function
Mfumv2_0573	2109			61	100	12	11	34	196		Polysulphide reductase
Mfumv2_0574	891	exo	*	232	159	102	50	173	317	*	5'-3' exonuclease
Mfumv2_0575	363	erpA		496	184	441	74	229	225		putative iron-sulfur cluster insertion protein ErpA
Mfumv2_0577	1254	proP		191	259	189	81	143	561		Permease of the major facilitator superfamily
Mfumv2_0578	633			2355	1485	1168	420	898	1906		conserved protein of unknown function
Mfumv2_0579	507	ruvC		205	98	244	42	196	235		component of RuvABC resolvasome, endonuclease
Mfumv2_0580	603	ruvA	*	45	33	83	17	220	471	*	Holliday junction ATP-dependent DNA helicase RuvA
Mfumv2_0581	1017	ruvB	*	190	192	183	76	195	599	*	ATP-dependent DNA helicase, component of RuvABC resolvasome
Mfumv2_0582	417			843	376	1799	235	730	933		conserved protein of unknown function
Mfumv2_0583	564	pspE		582	388	1683	279	689	926		Rhodanese-related sulfurtransferase

Mfumv2_0584	1251	smtA		720	978	454	289	1462	6007		SAM-dependent methyltransferase
Mfumv2_0585	453			606	329	866	144	1900	2174		conserved protein of unknown function
Mfumv2_0586	207			45	12	133	8	92	39		conserved protein of unknown function
Mfumv2_0587	183			51	14	269	15	83	25		conserved protein of unknown function
Mfumv2_0588	645	deoC	*	128	94	158	38	143	213	*	Deoxyribose-phosphate aldolase 1
Mfumv2_0589	1041			174	199	96	60	173	502		conserved protein of unknown function
Mfumv2_0590	729			27	25	27	7	54	118		Ribosomal RNA small subunit methyltransferase E
Mfumv2_0591	930	wcaG		95	99	78	25	142	356		Nucleoside-diphosphate-sugar epimerase
Mfumv2_0592	1551	clpX		345	587	191	140	399	1787		ATP-dependent protease Clp, ATPase subunit
Mfumv2_0593	345			8	2	26	2	34	15		conserved protein of unknown function
Mfumv2_0594	246			887	241	1194	159	411	218		conserved protein of unknown function
Mfumv2_0595	1179			241	309	224	99	405	1319		conserved protein of unknown function
Mfumv2_0596	1008			36	38	14	11	123	373		Alpha/beta hydrolase
Mfumv2_0597	1521	dhaS		1310	2183	1503	812	1125	5103		putative aldehyde dehydrogenase DhaS
Mfumv2_0598	282			410	56	630	29	167	48		conserved protein of unknown function
Mfumv2_0599	1047			177	113	472	96	103	87		protein of unknown function
Mfumv2_0600	147			366	31	312	13	474	45		exported protein of unknown function
Mfumv2_0601	678	rpe	*	217	151	172	47	231	442	*	Pentose-5-phosphate-3-epimerase
Mfumv2_0602	384	gloA		222	92	181	19	162	151		Lactoylglutathione lyase or related enzyme
Mfumv2_0603	1065	frvX		112	120	142	46	172	486		Cellulase M or related protein
Mfumv2_0604	303			791	230	1398	148	170	99		conserved protein of unknown function
Mfumv2_0605	219			2158	487	2964	264	2106	1767		conserved protein of unknown function
Mfumv2_0606	693	dedA		43	31	49	13	61	117		Protein DedA
Mfumv2_0607	489	ibpA		15620	6243	5433	921	1849	2984		Molecular chaperone, HSP20 family
Mfumv2_0608	984			933	959	988	384	912	2616		Opacity protein or related surface antigen
Mfumv2_0609	321			258	99	183	40	598	642		conserved exported protein of unknown function
Mfumv2_0610	624	azoR		258	213	193	59	584	1013		FMN-dependent NADH-azoreductase
Mfumv2_0611	1548			182	340	492	228	143	581		conserved membrane protein of unknown function
Mfumv2_0612	480	bcp		60	27	179	31	53	81		putative peroxiredoxin bcp
Mfumv2_0613	1362	glcD		202	320	275	127	249	1042		FAD/FMN-containing dehydrogenase
Mfumv2_0614	165			6603	1050	7456	558	1368	571		protein of unknown function
Mfumv2_0615	531			484	255	332	75	841	1319		conserved protein of unknown function
Mfumv2_0616	912	folB		238	224	166	68	316	706		Dihydroneopterin aldolase
Mfumv2_0617	450	fcbC		155	83	134	22	163	184		Thioesterase superfamily enzyme
Mfumv2_0618	996	qor		60	73	121	44	130	338		NADPH:quinone reductase or related Zn-dependent oxidoreductase
Mfumv2_0619	1563			184	294	181	122	245	1060		conserved protein of unknown function
Mfumv2_0620	1173	cysJ		454	578	281	173	622	1690		Sulfite reductase, alpha subunit (Flavoprotein)
Mfumv2_0621	948	manA		49	48	59	21	153	400		Phosphomannose isomerase
Mfumv2_0622	783			96	68	108	43	109	261		conserved protein of unknown function
Mfumv2_0623	684			501	338	564	129	367	690		Predicted phosphoribosyltransferase
Mfumv2_0624	2079	uvrB		278	719	325	250	357	1997		excinulease of nucleotide excision repair, DNA damage recognition component
Mfumv2_0625	759	pyrH		328	280	371	107	565	1418		uridylate kinase
Mfumv2_0626	561	frr	*	919	537	628	183	425	687	*	Ribosome-recycling factor
Mfumv2_0627	582			481	356	550	137	546	869		putative protease
Mfumv2_0628	1146	dadA	*	155	197	155	88	268	1013	*	Glycine/D-amino acid oxidase (Deaminating)
Mfumv2_0629	2601			101	324	71	99	146	1045		Endopolygalacturonase
Mfumv2_0630	1053			74	87	76	25	144	417		exported protein of unknown function
Mfumv2_0631	189	manC		0	0	41	0	0	0		Mannose-1-phosphate guanylyltransferase
Mfumv2_0632	756			562	411	444	171	719	1976		putative enzyme
Mfumv2_0633	174			5	1	0	0	0	0		conserved protein of unknown function
Mfumv2_0634	342			139	44	54	11	595	804		conserved protein of unknown function
Mfumv2_0635	336			98	34	87	11	177	148		conserved protein of unknown function
Mfumv2_0636	501	wecD		69	31	26	5	52	75		Acetyltransferase, GNAT family
Mfumv2_0637	1365	yfnA	*	291	400	349	162	185	715	*	Uncharacterized amino acid permease YfnA
Mfumv2_0638	207			70	16	136	6	38	14		conserved protein of unknown function
Mfumv2_0639	384			365	124	230	49	399	477		conserved exported protein of unknown function

Mfumv2_0640	525		*	27	21	23	6	179	255	*	RNA polymerase sigma-70 factor, ECF subfamily
Mfumv2_0641	162			389	60	273	22	507	291		conserved protein of unknown function
Mfumv2_0642	714			395	263	443	129	246	491		Transfer origin protein, TraL, ATPase
Mfumv2_0643	462			577	258	681	89	254	339		conserved exported protein of unknown function
Mfumv2_0644	417			1030	212	1197	133	438	94		transposase
Mfumv2_0645	1047			92	83	275	93	34	46		protein of unknown function
Mfumv2_0646	1047	hcaE		212	233	93	47	135	418		Ring-hydroxylating dioxygenase, large terminal subunit
Mfumv2_0647	942			538	469	559	196	379	929		conserved protein of unknown function
Mfumv2_0648	948			97	105	90	29	143	388		O-methyltransferase I
Mfumv2_0649	1224	acrA		82	116	62	34	63	245		Membrane-fusion protein
Mfumv2_0650	3231	acrB		74	258	50	63	84	764		Cation/multidrug efflux pump
Mfumv2_0651	453	slp		293	120	146	34	94	107		Starvation-inducible outer membrane lipoprotein
Mfumv2_0653	1359	mscS		58	86	55	28	80	323		Small-conductance mechanosensitive channel
Mfumv2_0654	765	ubiG		104	102	140	35	150	300		2-polyprenyl-3-methyl-5-hydroxy-6-metoxy-1,4-benzoquinol methylase
Mfumv2_0655	747	ubiG		47	45	52	10	91	175		2-polyprenyl-3-methyl-5-hydroxy-6-metoxy-1,4-benzoquinol methylase
Mfumv2_0656	1203	rfaG		57	80	55	25	95	289		Glycosyltransferase
Mfumv2_0657	807	cof		68	56	19	7	80	147		HAD superfamily hydrolase
Mfumv2_0658	642	tolQ		924	644	626	250	806	867		Biopolymer transport protein
Mfumv2_0659	447	exbD		266	142	167	44	526	377		Biopolymer transport protein
Mfumv2_0660	582	tonB		400	261	278	88	436	444		Periplasmic protein TonB
Mfumv2_0661	1488	trpE	*	124	203	60	50	259	1144	*	Anthranilate synthase component 1
Mfumv2_0662	606	pabA	*	301	204	229	69	338	456	*	aminodeoxychorismate synthase, subunit II
Mfumv2_0663	1257	gdhA		227	371	231	125	421	1470		Glutamate dehydrogenase
Mfumv2_0664	957			71	81	50	31	139	398		Dienelactone hydrolase or related enzyme (Modular protein)
Mfumv2_0665	2070	sqhC		648	1602	482	406	1416	9121		Squalene-hopene cyclase
Mfumv2_0666	696	pfs		581	513	676	152	791	1156		Nucleoside phosphorylase
Mfumv2_0667	342	sufA		269	109	166	31	262	191		Fe-S cluster assembly scaffold protein, HesB/SufA family
Mfumv2_0668	2481	leuS	*	230	682	310	338	618	4172	*	Leucine--tRNA ligase
Mfumv2_0669	1344	tldD	*	89	137	95	41	152	596	*	Predicted Zn-dependent protease, modulator of DNA gyrase, PmbA protein
Mfumv2_0670	1524	tldD	*	290	473	241	135	185	772	*	Predicted Zn-dependent protease, modulator of DNA gyrase, TldD protein
Mfumv2_0671	1248	argE	*	944	1325	1164	524	813	2752	*	Acetylornithine deacetylase/Succinyl-diaminopimelate desuccinylase
Mfumv2_0672	549	bfrB		713	396	524	94	888	1519		Ferritin BfrB
Mfumv2_0673	765	soj		93	74	130	39	186	449		Sporulation initiation inhibitor protein Soj
Mfumv2_0674	939			322	313	155	70	1041	3032		ABC-type transport system involved in gliding motility, ATPase component
Mfumv2_0675	750	nosY		78	73	54	19	322	732		ABC-type transport system involved in gliding motility, permease component
Mfumv2_0676	1479	gldG		315	557	295	214	605	2401		ABC-type transport system involved in gliding motility, auxiliary component
Mfumv2_0677	1800			308	710	166	169	519	2856		conserved protein of unknown function
Mfumv2_0678	555			176	94	176	40	265	401		conserved membrane protein of unknown function
Mfumv2_0679	612	acrR		85	45	30	11	143	227		Transcriptional regulator, TetR/AcrR family
Mfumv2_0680	1044	emrA		117	108	40	19	104	320		Multidrug resistance efflux pump
Mfumv2_0681	138			38	7	73	2	14	6		conserved protein of unknown function
Mfumv2_0682	1809	arnT		105	213	76	85	150	792		4-amino-4-deoxy-L-arabinose transferase or related glycosyltransferase of PMT family
Mfumv2_0683	183			358	77	28	4	135	67		conserved protein of unknown function
Mfumv2_0684	441	mutT		169	78	102	26	105	107		NUDIX family hydrolase
Mfumv2_0685	1359			125	150	78	46	101	383		Acid phosphatase
Mfumv2_0686	585			92	48	96	21	109	190		conserved protein of unknown function
Mfumv2_0687	525	coaD		147	94	202	37	265	352		Phosphopantetheine adenylyltransferase
Mfumv2_0688	1266	murA		556	745	765	380	450	1401		UDP-N-acetylglucosamine 1-carboxyvinyltransferase
Mfumv2_0689	828	hemK		144	135	198	61	368	642		Release factor glutamine methyltransferase
Mfumv2_0690	1137	prfA		1030	1376	974	475	967	2520		peptide chain release factor RF-1
Mfumv2_0691	243	rpmE	*	1667	497	1074	126	2495	2021	*	50S ribosomal protein L31
Mfumv2_0692	1245	fabF		1114	1733	1566	833	2120	5205		3-oxoacyl-[acyl-carrier-protein] synthase II
Mfumv2_0693	258	acpP		1259	402	1332	129	2591	1487		acyl carrier protein (ACP)
Mfumv2_0694	741	fabG		372	283	423	119	1281	2362		3-oxoacyl-[acyl-carrier-protein] reductase
Mfumv2_0695	921	fabD		149	148	175	51	472	1060		Malonyl CoA-acyl carrier protein transacylase
Mfumv2_0696	678	nth	*	331	271	446	110	400	625	*	Endonuclease III

Mfumv2_0697	1338	rimO	*	300	489	282	175	506	1896	*	Ribosomal protein S12 methylthiotransferase RimO
Mfumv2_0698	1134			225	313	150	86	443	1538		Predicted transcriptional regulator contains Xre-like HTH domain
Mfumv2_0699	2463	ftsK	*	304	817	253	243	366	2686	*	DNA segregation ATPase FtsK/SpoIIIE
Mfumv2_0700	636	sodA		460	290	288	76	862	1617		Superoxide dismutase [Mn]
Mfumv2_0701	297			370	108	172	23	449	356		conserved protein of unknown function
Mfumv2_0702	462			73	34	69	10	212	266		conserved protein of unknown function
Mfumv2_0703	777	his	*	145	136	75	27	559	1391	*	Histidinol phosphatase, PHP family
Mfumv2_0704	1038	erg		466	468	638	282	195	455		Squalene/phytoene synthase
Mfumv2_0705	612	coaE		1073	606	663	179	373	696		Dephospho-CoA kinase
Mfumv2_0706	1449	rho	*	793	1303	1265	550	1326	5326	*	transcription termination factor
Mfumv2_0707	213		*	2	1	53	3	9	4	*	transposase (fragment)
Mfumv2_0708	99			9	1	13	1	0	0		conserved protein of unknown function
Mfumv2_0709	966	galE		54	49	23	10	34	104		UDP-glucose 4-epimerase
Mfumv2_0710	906	oppC		15	14	20	6	37	95		oligopeptide transporter subunit ; membrane component of ABC superfamily
Mfumv2_0711	918	oppB		119	128	55	37	236	465		oligopeptide transporter subunit ; membrane component of ABC superfamily
Mfumv2_0712	354	rbfA	*	477	201	398	60	634	543	*	Ribosome-binding factor A
Mfumv2_0713	2088	infB	*	1035	2460	864	915	1114	6220	*	Translation initiation factor IF-2
Mfumv2_0714	1257	nusA	*	2343	2571	1444	798	1003	3517	*	Transcription termination/antitermination protein NusA
Mfumv2_0715	843			117	109	204	60	257	570		conserved protein of unknown function
Mfumv2_0716	927	mviM		144	157	211	69	191	484		Predicted dehydrogenase
Mfumv2_0717	1185	lpxB		114	158	112	60	210	731		Lipid-A-disaccharide synthase
Mfumv2_0718	1059			603	680	524	329	598	1944		conserved protein of unknown function
Mfumv2_0719	615	rpoE	*	1358	830	1576	325	1495	2887	*	DNA-directed RNA polymerase specialized sigma subunit
Mfumv2_0720	558			1136	656	884	177	561	813		conserved protein of unknown function
Mfumv2_0721	1119	degQ		356	477	205	121	459	1518		Serine protease Do (Heat-shock protein)
Mfumv2_0722	927	motB		1013	1008	862	333	512	1400		Flagellar motor protein
Mfumv2_0723	186	rpsU	*	2862	509	2465	231	1309	471	*	30S ribosomal protein S21
Mfumv2_0724	996	adhA		252	310	218	77	288	612		putative alcohol dehydrogenase AdhA
Mfumv2_0725	72			0	0	0	0	3	1		protein of unknown function
Mfumv2_0726	147			0	0	31	1	0	0		conserved protein of unknown function
Mfumv2_0727	192			12	1	0	0	0	0		conserved protein of unknown function
Mfumv2_0728	222			2	1	0	0	6	3		conserved protein of unknown function
Mfumv2_0729	2478			83	241	83	80	144	1003		Glycosyltransferase
Mfumv2_0730	147			59	7	61	7	24	6		conserved protein of unknown function
Mfumv2_0731	162			233	40	569	30	100	36		protein of unknown function
Mfumv2_0732	381			194	0	300	0	45	0		conserved protein of unknown function
Mfumv2_0733	492			142	0	403	0	38	0		RNA-directed DNA polymerase (Reverse transcriptase)
Mfumv2_0734	1620			428	610	1017	511	299	968		conserved protein of unknown function
Mfumv2_0735	129			0	0	0	0	0	0		conserved protein of unknown function
Mfumv2_0736	3879			654	3026	1380	1745	384	3022		conserved protein of unknown function
Mfumv2_0737	177			0	0	0	0	4	3		protein of unknown function
Mfumv2_0738	348			3	1	0	0	6	3		conserved protein of unknown function
Mfumv2_0739	405			16	5	11	1	4	5		conserved protein of unknown function
Mfumv2_0740	798	rfbF		611	570	1299	315	827	1815		Glucose-1-phosphate cytidylyltransferase
Mfumv2_0741	996	wcaG		617	821	1008	393	854	2254		Nucleoside-diphosphate-sugar epimerase
Mfumv2_0742	546	rmlC		202	151	440	83	564	943		dTDP-4-dehydrorhamnose 3,5-epimerase
Mfumv2_0743	933	galE		177	218	226	81	388	864		UDP-glucose 4-epimerase
Mfumv2_0745	1197	lhgO		114	167	219	119	347	1215		L-2-hydroxyglutarate oxidase LhgO
Mfumv2_0746	417			766	189	924	119	421	88		transposase
Mfumv2_0747	1047			25	17	145	28	12	9		protein of unknown function
Mfumv2_0748	1200	kefB		41	48	25	14	30	96		Na+/H+ antiporter
Mfumv2_0749	1137	nemA		248	341	334	162	405	1250		N-ethylmaleimide reductase, FMN-linked
Mfumv2_0750	672		*	158	121	263	64	153	262	*	Glutamine amidotransferase
Mfumv2_0751	2001	hyfB		185	455	187	232	246	1227		Formate hydrogenlyase subunit 3
Mfumv2_0752	951	hycD		98	137	212	110	209	501		Formate hydrogenlyase subunit 4
Mfumv2_0753	663	hyfA		130	109	443	129	228	334		Formate hydrogenlyase membrane component

Mfumv2_0754	1479	hyfF		113	205	244	158	151	437		Hydrogenase-4 component F homolog
Mfumv2_0755	1533	hycE		142	288	486	284	252	923		putative formate hydrogenlyase
Mfumv2_0756	537	mbhJ		198	157	789	121	233	266		putative membrane-bound hydrogenase subunit mbhJ
Mfumv2_0757	126			89	11	56	3	275	128		protein of unknown function
Mfumv2_0758	177			48	8	0	0	19	8		conserved protein of unknown function
Mfumv2_0759	1242			14	21	2	2	24	75		conserved protein of unknown function
Mfumv2_0760	345			2440	902	2694	486	1678	2061		conserved protein of unknown function
Mfumv2_0761	393			944	348	788	133	321	324		conserved protein of unknown function
Mfumv2_0762	192			341	78	232	18	289	144		conserved protein of unknown function
Mfumv2_0763	390			447	189	424	79	268	292		conserved protein of unknown function
Mfumv2_0764	468			139	72	256	54	93	103		Serine kinase of the HPr protein, regulates carbohydrate metabolism
Mfumv2_0765	1017			51	60	154	69	68	146		Radical SAM superfamily enzyme
Mfumv2_0766	306	ppqD		144	47	242	37	60	39		Coenzyme PQQ synthesis protein D
Mfumv2_0767	2130	acrA		28	68	83	63	63	372		Membrane-fusion protein
Mfumv2_0768	909	acrA		158	165	242	82	228	585		Membrane-fusion protein
Mfumv2_0769	2172			48	113	38	33	89	503		Membrane-fusion protein , contains peptidase family M50 domain
Mfumv2_0770	1518			32	43	38	16	76	318		conserved protein of unknown function
Mfumv2_0771	912			19	18	41	9	31	71		conserved protein of unknown function
Mfumv2_0772	2400			47	130	53	37	74	509		conserved protein of unknown function
Mfumv2_0773	858		*	63	54	124	36	72	174	*	putative Transglutaminase-like enzyme, cysteine protease
Mfumv2_0774	684	spr	*	61	43	61	15	119	259	*	Cell wall-associated hydrolase
Mfumv2_0775	153			51	9	99	6	56	19		conserved protein of unknown function
Mfumv2_0776	147			0	0	0	0	2	1		conserved protein of unknown function
Mfumv2_0777	774	csgD		49	33	42	16	55	83		Transcriptional regulator LuxR family
Mfumv2_0778	189			63	12	27	4	28	9		conserved protein of unknown function
Mfumv2_0779	681	ompR		211	146	172	68	330	645		DNA-binding response regulator, OmpR family (REC-wHTH domains)
Mfumv2_0780	231			90	10	95	8	23	7		protein of unknown function
Mfumv2_0781	1047			7	4	18	8	7	7		protein of unknown function
Mfumv2_0782	510			90	39	89	19	151	282		conserved exported protein of unknown function
Mfumv2_0783	909			44	41	56	19	63	155		conserved exported protein of unknown function
Mfumv2_0784	1626	gntK		109	207	113	70	129	555		Predicted APH family phosphotransferase fused to gluconate kinase family enzyme
Mfumv2_0785	2076	hyuA	*	111	283	197	126	221	1079	*	N-methylhydantoinase A/acetone carboxylase, beta subunit
Mfumv2_0786	1566		*	112	198	98	56	262	1165	*	conserved protein of unknown function
Mfumv2_0787	552			69	41	38	14	69	90		conserved exported protein of unknown function
Mfumv2_0788	3180	czcA		60	258	55	84	135	994		Cation efflux system protein CzcA
Mfumv2_0789	867	acrA		43	53	56	22	148	291		Membrane-fusion protein
Mfumv2_0790	1314	tolC		56	107	49	23	141	430		Heavy metal RND efflux outer membrane protein,CzcC family
Mfumv2_0791	1344	tolC		68	95	36	16	102	340		Heavy metal RND efflux outer membrane protein,CzcC family
Mfumv2_0792	750	gpmA		382	291	298	90	418	881		phosphoglyceromutase 1
Mfumv2_0793	1407	cusS		41	67	37	23	73	247		Copper sensor histidine kinase
Mfumv2_0794	672	czcR		233	164	302	69	223	394		Transcriptional activator protein CzcR
Mfumv2_0795	1089	sgaA	*	389	454	350	133	1751	6033	*	Serine-glyoxylate aminotransferase
Mfumv2_0796	153			16	2	0	0	96	32		protein of unknown function
Mfumv2_0797	798			104	85	92	26	121	232		conserved exported protein of unknown function
Mfumv2_0798	1065	namA		137	156	81	44	141	451		NADPH dehydrogenase
Mfumv2_0799	690	rpe		519	367	577	177	491	1030		Ribulose-phosphate 3-epimerase
Mfumv2_0800	1404	prpD		692	1097	1037	570	471	1439		2-methylcitrate dehydratase
Mfumv2_0801	1620			2021	1744	5741	1783	630	1222		conserved protein of unknown function
Mfumv2_0802	1404	mtoX		215	302	93	53	173	590		SBP56, 56kDa selenium binding protein
Mfumv2_0803	618			76	47	74	23	74	122		conserved membrane protein of unknown function
Mfumv2_0804	240			390	91	736	62	150	116		conserved protein of unknown function
Mfumv2_0805	1341			165	219	310	133	126	399		conserved protein of unknown function
Mfumv2_0806	1038			81	73	138	53	100	186		conserved protein of unknown function
Mfumv2_0807	306			84	23	127	11	114	92		conserved protein of unknown function
Mfumv2_0808	621	glpF		218	124	149	49	215	314		Glycerol uptake facilitator or related permease (Major Intrinsic Protein Family)
Mfumv2_0809	1422	phoD		142	214	160	93	74	253		Phosphodiesterase/alkaline phosphatase D

Mfumv2_0810	324			2933	954	2218	330	1377	1656		conserved exported protein of unknown function
Mfumv2_0811	1185	dinB	*	71	93	92	45	86	312	*	DNA polymerase IV
Mfumv2_0812	117			0	0	0	0	10	3		conserved protein of unknown function
Mfumv2_0813	1005	queA	*	258	258	204	99	153	393	*	S-adenosylmethionine:tRNA ribosyltransferase-isomerase
Mfumv2_0814	831	phnF		1934	1695	517	213	780	1633		Transcriptional regulator, GntR family
Mfumv2_0815	1728	cysI		1348	3329	713	569	1072	3341		Sulfite reductase [NADPH] hemoprotein beta-component
Mfumv2_0816	444	uspA		673	294	360	77	438	655		Universal stress protein
Mfumv2_0817	582	trxA		455	267	242	66	548	964		Thiol-disulfide isomerase or thioredoxin
Mfumv2_0818	312			260	88	89	10	190	125		conserved protein of unknown function
Mfumv2_0819	600	recR		413	265	748	153	436	508		Recombination protein RecR
Mfumv2_0820	309			1327	363	1497	192	576	319		Nucleoid-associated protein Minf_1672
Mfumv2_0821	1779	dnaX	*	328	647	409	257	529	2537	*	DNA polymerase III, gamma/tau subunit
Mfumv2_0822	150			77	12	114	10	215	96		most likely a pseudogene; at this position the SRP_RNA is encoded
Mfumv2_0823	489			808	527	1075	249	2073	2902		conserved protein of unknown function
Mfumv2_0824	2166	glyA		421	1047	362	422	630	3260		Serine hydroxymethyltransferase
Mfumv2_0825	834	accD		166	143	76	36	180	435		acetyl-CoA carboxylase, beta (carboxyltranferase) subunit
Mfumv2_0826	96			41	6	47	1	38	13		conserved protein of unknown function
Mfumv2_0827	75			0	0	0	0	0	0		conserved protein of unknown function
Mfumv2_0828	198			0	0	0	0	13	4		conserved protein of unknown function
Mfumv2_0830	177			0	0	0	0	14	6		conserved protein of unknown function
Mfumv2_0831	303			103	29	61	12	175	191		conserved exported protein of unknown function
Mfumv2_0832	138			104	18	221	8	62	19		conserved protein of unknown function
Mfumv2_0833	264			49	15	89	10	46	19		conserved protein of unknown function
Mfumv2_0834	141			103	13	85	6	44	11		protein of unknown function
Mfumv2_0835	201			720	141	1085	75	216	96		conserved protein of unknown function
Mfumv2_0837	216			18	3	0	0	3	1		conserved protein of unknown function
Mfumv2_0838	738			49	42	58	20	22	51		conserved protein of unknown function
Mfumv2_0839	2823	cirA		15	45	14	10	25	202		Outer membrane receptor protein, mostly Fe transport
Mfumv2_0840	588			29	15	2	1	9	15		conserved protein of unknown function
Mfumv2_0841	153			49	8	38	2	5	3		conserved protein of unknown function
Mfumv2_0842	450			58	27	16	3	43	64		Predicted amidophosphoribosyltransferase ComF (fragment)
Mfumv2_0843	852			54	46	34	11	84	252		CAAX amino terminal protease family
Mfumv2_0844	966	accA		324	350	329	129	162	519		Acetyl-coenzyme A carboxylase carboxyl transferase subunit alpha
Mfumv2_0845	435	ribH		301	114	136	32	93	120		6,7-dimethyl-8-ribityllumazine synthase
Mfumv2_0846	432	nusB		202	87	362	41	297	436		N utilization substance protein B homolog
Mfumv2_0847	849	ftsY	*	194	195	275	79	286	657	*	Signal recognition particle GTPase FtsY
Mfumv2_0848	1098	ribD		212	268	237	100	162	448		Riboflavin biosynthesis protein RibD
Mfumv2_0849	564	hpt		30	21	48	6	69	119		Hypoxanthine-guanine phosphoribosyltransferase
Mfumv2_0850	582			44	24	42	8	70	119		Predicted membrane protein (Modular protein)
Mfumv2_0851	1584	lnt		25	40	21	13	35	133		Apolipoprotein N-acyltransferase
Mfumv2_0852	1527	ppiD		126	210	114	78	210	1010		Peptidyl-prolyl cis-trans isomerase
Mfumv2_0853	1110			91	106	52	24	179	678		Predicted permease
Mfumv2_0854	591	gmhA		267	171	216	66	238	395		Phosphoheptose isomerase
Mfumv2_0855	447	accB		326	142	197	41	325	438		Biotin carboxyl carrier protein
Mfumv2_0856	198			118	28	241	18	122	52		conserved protein of unknown function
Mfumv2_0857	861	holB	*	139	153	287	71	225	539	*	ATPase involved in DNA replication HolB
Mfumv2_0858	642	tmk		101	86	164	44	181	299		Thymidylate kinase
Mfumv2_0859	1407	cysS	*	109	190	65	47	219	874	*	Cysteine--tRNA ligase
Mfumv2_0860	1422	lpd	*	82	140	137	66	202	626	*	Pyruvate/2-oxoglutarate dehydrogenase complex,dihydrolipoamide dehydrogenase (E3) component
Mfumv2_0861	693			89	70	70	22	179	281		HAD family hydrolase
Mfumv2_0862	783	fabI		125	104	149	45	240	544		Enoyl-[acyl-carrier-protein] reductase [NADH] FabI
Mfumv2_0863	180			132	20	109	7	22	8		conserved protein of unknown function
Mfumv2_0864	195			0	0	0	0	1	1		conserved protein of unknown function
Mfumv2_0865	375			15	4	3	1	5	4		transposase
Mfumv2_0866	126			0	0	36	1	18	3		conserved protein of unknown function
Mfumv2_0867	1047			64	42	171	50	35	36		protein of unknown function

Mfumv2_0868	417			980	102	940	67	169	79		transposase
Mfumv2_0869	177			1050	131	2227	108	575	71		conserved protein of unknown function
Mfumv2_0870	117			865	107	2183	74	666	225		protein of unknown function
Mfumv2_0871	1938			826	1828	1643	1029	536	2457		UDP-N-acetylglucosamine:LPS N-acetylglucosamine transferase fused to PHP family phosphoesterase
Mfumv2_0872	990	birA		209	233	746	166	146	362		Biotin-(Acetyl-CoA carboxylase) ligase
Mfumv2_0873	816	gloB		1359	1094	1564	471	734	1548		Zn-dependent hydrolase, glyoxylase family
Mfumv2_0874	441	phyH		11371	5614	20372	3330	6848	7804		Phytanoyl-CoA dioxygenase
Mfumv2_0875	978	lraI		119	125	98	39	173	370		ABC-type Mn2+/Zn2+ transport system, periplasmic component
Mfumv2_0876	762	znuC		92	76	105	26	103	163		ABC-type Mn2+/Zn2+ transport system, ATPase component
Mfumv2_0877	867	znuB		75	69	45	19	69	159		ABC-type Mn2+/Zn2+ transport system, permease component
Mfumv2_0878	630	gph		41	32	32	9	81	139		Phosphoglycolate phosphatase
Mfumv2_0879	1044		*	161	177	121	61	130	362	*	3-isopropylmalate dehydrogenase
Mfumv2_0880	1836	yitJ		171	351	238	168	183	933		Bifunctional homocysteine S-methyltransferase/5,10-methylenetetrahydrofolate reductase
Mfumv2_0881	1299	htpX		255	344	136	87	188	661		CAAX family Zn-dependent protease
Mfumv2_0883	618			118	68	68	13	87	169		conserved exported protein of unknown function
Mfumv2_0884	1140	dsbG	*	55	60	35	14	85	307	*	Protein-disulfide isomerase
Mfumv2_0885	798			59	54	64	24	84	162		PIN-domain and Zn ribbon
Mfumv2_0886	810	hoxQ		383	313	239	106	181	398		Hydrogenase expression/formation protein hoxQ
Mfumv2_0887	1530	mdoG		58	98	42	28	98	442		Periplasmic glucans biosynthesis protein G
Mfumv2_0888	1536	mdoG		107	209	147	93	234	935		Periplasmic glucans biosynthesis protein G
Mfumv2_0889	393			65	37	269	29	103	80		conserved protein of unknown function
Mfumv2_0890	2079	mdoH		38	82	46	32	80	405		glucan biosynthesis: glycosyl transferase
Mfumv2_0891	480	hycL		237	115	329	63	111	117		putative hydrogenase maturation protease
Mfumv2_0892	342	hypA		161	53	133	18	108	109		putative hydrogenase nickel incorporation protein HypA
Mfumv2_0893	693	nfi	*	136	90	207	41	107	177	*	Deoxyinosine 3'endonuclease/endonuclease V
Mfumv2_0894	303			3569	1016	2424	279	1418	1632		conserved protein of unknown function
Mfumv2_0895	120			16	2	124	2	6	2		protein of unknown function
Mfumv2_0896	141			9	0	0	0	14	0		conserved protein of unknown function
Mfumv2_0898	156			32	7	0	0	11	7		conserved protein of unknown function
Mfumv2_0899	123			0	0	10	1	16	6		conserved protein of unknown function
Mfumv2_0900	1014			76	81	52	20	54	150		putative Methylase involved in ubiquinone/menaquinone biosynthesis
Mfumv2_0902	585	cynT		386	217	236	66	1103	2398		Carbonic anhydrase
Mfumv2_0903	381			95	33	54	5	58	66		conserved protein of unknown function
Mfumv2_0904	174			33	4	0	0	43	25		conserved protein of unknown function
Mfumv2_0905	267			54	17	10	2	85	78		conserved protein of unknown function
Mfumv2_0906	441			19	12	18	3	68	79		conserved protein of unknown function
Mfumv2_0907	693	pflA		159	129	65	24	196	336		Ribonucleotide reductase of class III (Anaerobic), activating protein
Mfumv2_0908	1836	nrdD		192	423	80	79	267	1169		Ribonucleotide reductase of class III (Anaerobic), large subunit
Mfumv2_0909	381			315	128	107	20	477	608		conserved protein of unknown function
Mfumv2_0910	1035	rfaG		193	223	114	59	215	611		Glycosyltransferase
Mfumv2_0911	582			224	103	303	65	121	116		conserved protein of unknown function
Mfumv2_0912	696	dsbG	*	66	46	97	27	128	243	*	Protein-disulfide isomerase
Mfumv2_0913	297			90	30	74	8	362	401		conserved protein of unknown function
Mfumv2_0914	789	phnP		280	213	193	75	162	353		Metal-dependent hydrolase of the beta-lactamase superfamily
Mfumv2_0915	1386	rpoN	*	192	295	210	82	162	545	*	DNA-directed RNA polymerase specialized sigma subunit
Mfumv2_0916	177			0	0	0	0	27	16		conserved protein of unknown function
Mfumv2_0917	213	yrzS		182	36	169	13	112	80		Uncharacterized membrane protein YrzS
Mfumv2_0918	2748	alaS	*	209	793	269	309	435	3384	*	Alanine--tRNA ligase
Mfumv2_0919	747			575	451	871	201	461	980		putative transcriptional regulatory protein Minf_0651
Mfumv2_0920	1137	rnd		394	466	228	143	225	656		Ribonuclease D
Mfumv2_0921	1188	moeZ		1683	2125	1763	851	1333	4151		Sulfur carrier protein CysO adenylyltransferase ; Sulfur carrier protein CysO sulfurtransferase
Mfumv2_0922	306	clpS		1541	447	336	55	1554	1507		ATP-dependent Clp protease adapter protein ClpS
Mfumv2_0923	510			437	238	173	36	295	369		conserved protein of unknown function
Mfumv2_0924	2460	glgP	*	258	701	178	213	440	2961	*	Glycogen phosphorylase
Mfumv2_0925	1344			567	842	616	368	470	1619		conserved protein of unknown function
Mfumv2_0926	189			560	109	1018	85	223	85		conserved protein of unknown function

Mfumv2_0927	138			29	3	0	0	33	15		conserved protein of unknown function
Mfumv2_0928	141			23	0	0	0	9	0		conserved protein of unknown function
Mfumv2_0929	102			42	2	121	1	12	2		protein of unknown function
Mfumv2_0930	381			53	23	44	8	92	102		conserved protein of unknown function
Mfumv2_0931	2793	mgtB		91	265	84	86	174	1449		Magnesium-transporting ATPase, P-type 1
Mfumv2_0933	972	kdsD		848	850	563	289	2096	7417		Arabinose 5-phosphate isomerase KdsD
Mfumv2_0934	561	efp	*	1901	1127	1624	361	1336	2206	*	Elongation factor P
Mfumv2_0935	846	gloB		394	381	360	125	368	722		Zn-dependent hydrolase, glyoxylase family
Mfumv2_0936	969	prs	*	345	364	339	143	1051	2848	*	Ribose-phosphate pyrophosphokinase
Mfumv2_0937	510	yugG		312	166	327	73	551	718		Uncharacterized HTH-type transcriptional regulator YugG
Mfumv2_0938	1236	yugH	*	246	411	265	145	712	2113	*	putative aminotransferase YugH
Mfumv2_0939	462			1483	651	1297	329	1182	1544		ACT domain-containing protein
Mfumv2_0940	1191	metK	*	802	1085	885	372	1032	3468	*	methionine adenosyltransferase 1
Mfumv2_0941	1320	ahcY		722	1145	1409	733	1281	4219		Adenosylhomocysteinase
Mfumv2_0942	459		*	103	49	116	24	224	209	*	Cytochrome c family protein
Mfumv2_0943	1305			70	112	171	61	143	384		Sulfite oxidase or related enzyme
Mfumv2_0944	672			163	128	148	57	222	471		Uncharacterized erfK/srfK family protein (Modular protein)
Mfumv2_0945	162			92	17	24	3	215	113		conserved protein of unknown function
Mfumv2_0946	549			49	35	66	16	98	149		N6-adenine-specific methylase
Mfumv2_0947	1263			68	77	39	13	66	228		AsmA family protein
Mfumv2_0948	882	lcb		86	84	43	18	113	315		Diacylglycerol kinase family enzyme
Mfumv2_0949	426	rpsL	*	1768	707	615	130	2198	2679	*	30S ribosomal protein S12
Mfumv2_0950	153			854	130	42	5	605	292		protein of unknown function
Mfumv2_0951	471	rpsG	*	3542	1521	2481	686	2859	4313	*	30S ribosomal subunit protein S7
Mfumv2_0952	2172	fusA	*	1078	3031	1083	1115	2848	18220	*	protein chain elongation factor EF-G, GTP-binding
Mfumv2_0953	306	rpsJ	*	704	292	551	84	1625	1069	*	30S ribosomal protein S10
Mfumv2_0954	651	rplC	*	877	764	716	270	1750	3804	*	50S ribosomal subunit protein L3
Mfumv2_0955	657	rplD	*	999	846	895	371	1168	2050	*	50S ribosomal protein L4
Mfumv2_0956	285	rplW	*	576	216	465	63	1864	1871	*	50S ribosomal protein L23
Mfumv2_0957	852	rplB	*	1227	1333	941	397	1994	5648	*	50S ribosomal subunit protein L2
Mfumv2_0958	276	rpsS	*	1345	429	1360	202	1279	949	*	30S ribosomal protein S19
Mfumv2_0959	339	rplV	*	1590	644	1821	283	2341	2084	*	50S ribosomal subunit protein L22
Mfumv2_0960	648	rpsC	*	1015	902	806	226	2659	5814	*	30S ribosomal subunit protein S3
Mfumv2_0961	426	rplP	*	1699	966	2916	605	2762	2662	*	50S ribosomal subunit protein L16
Mfumv2_0962	213	rpmC	*	1092	335	896	112	2117	1428	*	50S ribosomal protein L29
Mfumv2_0963	294	rpsQ		1977	759	1830	282	3054	3066		30S ribosomal protein S17
Mfumv2_0964	366	rplN	*	1967	932	1748	285	2578	2640	*	50S ribosomal subunit protein L14
Mfumv2_0965	315	rplX	*	1467	605	1105	143	1868	1713	*	50S ribosomal protein L24
Mfumv2_0966	582	rplE	*	1093	790	975	228	1865	3290	*	50S ribosomal subunit protein L5
Mfumv2_0967	387	rpsH	*	548	290	488	98	1829	2575	*	30S ribosomal subunit protein S8
Mfumv2_0968	540	rplF	*	964	655	801	217	1642	2602	*	50S ribosomal subunit protein L6
Mfumv2_0969	354	rplR	*	348	171	248	51	1220	1553	*	50S ribosomal subunit protein L18
Mfumv2_0970	639	rpsE	*	1442	1122	1175	418	2414	4873	*	30S ribosomal protein S5
Mfumv2_0971	444	rplO	*	2612	1370	2319	456	2584	2437	*	50S ribosomal subunit protein L15
Mfumv2_0972	1491	secY		643	1259	819	508	817	3389		preprotein translocase membrane subunit
Mfumv2_0973	912	emrA		249	226	178	85	156	319		Multidrug resistance efflux pump
Mfumv2_0974	1953			79	165	66	57	149	859		conserved protein of unknown function
Mfumv2_0975	633			346	220	243	78	470	1092		Predicted proline hydroxylase
Mfumv2_0977	180			48	8	50	7	26	13		conserved protein of unknown function
Mfumv2_0978	1056	hhyS		4526	5015	4881	2323	2411	6904		Nickel/iron-hydrogenase I small subunit Group 1h/5
Mfumv2_0979	1815	hhyL		2228	4719	2028	1499	2359	12144		Nickel/iron-hydrogenase I large subunit Group 1h/5
Mfumv2_0980	546	hupD		342	210	660	121	523	658		Putative maturation protein
Mfumv2_0981	1245			260	340	207	113	257	766		conserved protein of unknown function
Mfumv2_0982	636			279	193	235	71	293	491		conserved protein of unknown function
Mfumv2_0984	669			23	12	17	3	11	17		Outer membrane receptor protein, mostly Fe transport
Mfumv2_0985	654			225	135	235	65	97	140		conserved protein of unknown function

Mfumv2_0986	330			67	26	48	4	39	33		conserved protein of unknown function
Mfumv2_0988	801	hhaB		1320	1082	1145	363	481	966		Hydrogenase nickel incorporation protein hhaB
Mfumv2_0989	711			313	250	259	88	274	528		High-affinity nickel transporter
Mfumv2_0990	2358	hhaF		65	185	53	76	101	628		(NiFe) hydrogenase metallocenter assembly protein HhaF
Mfumv2_0991	375	hhaC		420	162	399	39	724	810		(NiFe) hydrogenase metallocenter assembly protein HhaC
Mfumv2_0992	1113	hhaD		789	963	919	350	1113	3462		(NiFe) hydrogenase metallocenter assembly protein HhaD
Mfumv2_0993	1017	hhaE		936	1068	1435	493	556	1354		(NiFe) hydrogenase metallocenter assembly protein HhaE
Mfumv2_0994	1206	gmhA		281	376	503	202	313	1087		Phosphoheptose isomerase
Mfumv2_0995	126			10	2	0	0	14	5		conserved protein of unknown function
Mfumv2_0996	381			20	11	13	4	36	46		conserved exported protein of unknown function
Mfumv2_0997	1620			586	724	1443	694	230	549		conserved protein of unknown function
Mfumv2_0998	474			19	9	42	7	26	37		conserved exported protein of unknown function
Mfumv2_0999	126			0	0	0	0	0	0		conserved protein of unknown function
Mfumv2_1000	126			74	12	118	4	91	39		protein of unknown function
Mfumv2_1001	1620			827	882	2294	960	546	1195		conserved protein of unknown function
Mfumv2_1002	129			6	2	78	2	8	2		conserved protein of unknown function
Mfumv2_1003	177			18	3	7	1	8	2		conserved protein of unknown function
Mfumv2_1004	282			7	2	0	0	3	1		conserved protein of unknown function
Mfumv2_1005	366			2478	884	1566	274	417	459		conserved protein of unknown function
Mfumv2_1006	165			144	31	178	13	62	17		conserved protein of unknown function
Mfumv2_1007	777			135	100	98	36	271	626		conserved membrane protein of unknown function
Mfumv2_1008	747	ttg2A		298	241	223	73	339	704		Toluene tolerance efflux transporter (ABC superfamily, atp_bind)
Mfumv2_1009	816	ttg2C		287	272	261	92	359	855		Organic solvents resistance ABC-type transport system, periplasmic component
Mfumv2_1010	696	ispD		404	291	234	94	486	1104		2-C-methyl-D-erythritol 4-phosphate cytidylyltransferase
Mfumv2_1011	168	rpmG	*	1399	203	252	28	624	389	*	50S ribosomal protein L33
Mfumv2_1012	894			176	166	135	52	136	362		CHAD domain containing protein
Mfumv2_1013	1320			105	157	124	50	72	239		conserved exported protein of unknown function
Mfumv2_1014	1671	glgP	*	169	302	291	151	125	539	*	Glucan phosphorylase
Mfumv2_1015	852			150	154	182	50	486	1282		conserved protein of unknown function
Mfumv2_1016	1392	fumC	*	327	559	489	295	368	1053	*	fumarate hydratase (fumarase C),aerobic Class II
Mfumv2_1017	522	rfaE		218	139	252	50	509	834		ADP-heptose synthase, bifunctional sugar kinase/adenylyltransferase
Mfumv2_1018	609	purN		165	131	168	35	354	440		Phosphoribosylglycinamide formyltransferase
Mfumv2_1019	1353	xseA		257	455	579	275	357	1084		Exodeoxyribonuclease 7 large subunit
Mfumv2_1020	237			496	107	755	59	428	184		conserved protein of unknown function
Mfumv2_1021	198			22	3	188	6	42	8		conserved protein of unknown function
Mfumv2_1022	804			72	50	191	36	62	54		conserved protein of unknown function
Mfumv2_1023	183			46	7	21	3	15	5		conserved protein of unknown function
Mfumv2_1024	1185	tufB		4266	5390	4678	1853	6809	20051		protein chain elongation factor EF-Tu, possible GTP-binding factor (duplicate of tufA)
Mfumv2_1025	303	secE		1754	588	925	136	2610	2407		Preprotein translocase subunit SecE
Mfumv2_1026	603	nusG		1374	934	1414	330	4443	8200		transcription termination factor
Mfumv2_1027	426	rplK	*	1881	887	1016	236	2499	3115	*	50S ribosomal protein L11
Mfumv2_1028	720	rplA	*	1226	1071	1049	396	2452	4182	*	50S ribosomal subunit protein L1
Mfumv2_1029	537	rplJ	*	559	367	521	164	1088	1548	*	50S ribosomal protein L10
Mfumv2_1030	390	rplL	*	5145	2244	6043	1162	5288	3653	*	50S ribosomal subunit protein L7/L12
Mfumv2_1031	3870	rpoB	*	1006	5138	1612	2601	1869	16343	*	RNA polymerase, beta subunit
Mfumv2_1032	4173	rpoC	*	1069	5897	1224	2235	1692	19625	*	RNA polymerase, beta prime subunit
Mfumv2_1033	891	folD		257	256	173	58	261	521		Bifunctional protein FolD [Methylenetetrahydrofolate dehydrogenase ; Methenyltetrahydrofolate cyclohydrolase]
Mfumv2_1034	837	wcaG		84	60	44	16	59	150		Nucleoside-diphosphate-sugar epimerase
Mfumv2_1035	810	proB	*	539	491	318	130	867	2313	*	Glutamate 5-kinase
Mfumv2_1036	1263	proA	*	348	517	300	150	482	1608	*	gamma-glutamylphosphate reductase
Mfumv2_1037	666			1920	1222	1443	470	706	1446		conserved protein of unknown function
Mfumv2_1038	1110			24	28	3	3	13	43		conserved protein of unknown function
Mfumv2_1039	222			0	0	0	0	0	0		protein of unknown function
Mfumv2_1041	474			20	8	24	3	7	5		conserved protein of unknown function
Mfumv2_1042	249			0	0	5	1	0	0		conserved protein of unknown function
Mfumv2_1043	807	uppP		174	142	101	41	119	236		Undecaprenyl-diphosphatase

Mfumv2_1044	963	dacC		315	374	187	109	413	1236		D-alanyl-D-alanine carboxypeptidase (modular protein)
Mfumv2_1045	903	hisG	*	1392	1190	805	393	4191	13069	*	ATP phosphoribosyltransferase
Mfumv2_1046	510			363	190	248	57	461	734		AP-4-A phosphorylase
Mfumv2_1047	1131	holA	*	37	58	93	35	81	312	*	DNA polymerase III, delta subunit
Mfumv2_1048	768	wcaA		279	237	265	71	218	456		Glycosyltransferase
Mfumv2_1049	573	adk	*	209	122	151	40	208	356	*	Adenylate kinase
Mfumv2_1050	795	tolQ		366	291	269	100	230	535		Biopolymer transport protein ExbB/TolQ
Mfumv2_1051	429	exbD		409	198	283	49	230	256		Biopolymer transport protein ExbD/TolR
Mfumv2_1052	2361	nrfG		151	393	95	97	139	1057		TPR repeats containing protein
Mfumv2_1053	1077	ompA		211	291	177	98	205	588		Outer membrane protein or related peptidoglycan-associated (Lipo)protein
Mfumv2_1054	570			163	79	110	39	57	89		conserved protein of unknown function
Mfumv2_1056	1791	nrfG		105	218	66	44	149	735		TPR repeats containing protein
Mfumv2_1057	840			55	48	36	13	57	139		Uncharacterized membrane protein, predicted transporter
Mfumv2_1058	1071			160	169	101	49	98	320		conserved protein of unknown function
Mfumv2_1060	2799		*	127	409	125	136	164	1522	*	Inactivated superfamily I helicase and RecB family exonuclease
Mfumv2_1061	3186	recB	*	74	276	84	94	78	643	*	ATP-dependent exoDNAse (Exonuclease V) beta subunit (Contains helicase and exonuclease domains)
Mfumv2_1062	378			234	94	498	55	603	577		protein of unknown function
Mfumv2_1063	1578			704	1200	1939	994	394	1084		protein of unknown function
Mfumv2_1064	174			0	0	0	0	0	0		HNH endonuclease
Mfumv2_1065	486		*	86	45	127	19	93	117	*	conserved exported protein of unknown function
Mfumv2_1066	297			146	54	136	14	277	273		conserved protein of unknown function
Mfumv2_1067	783	tatC		246	204	200	63	155	295		Sec-independent protein translocase protein TatC
Mfumv2_1068	693			579	425	284	95	646	1414		conserved protein of unknown function
Mfumv2_1069	147			19	3	68	2	2	1		conserved protein of unknown function
Mfumv2_1070	168			336	47	409	31	92	24		conserved protein of unknown function
Mfumv2_1071	216			9	2	26	1	1	1		conserved protein of unknown function
Mfumv2_1072	264			14	3	17	1	8	3		conserved protein of unknown function
Mfumv2_1073	1347	pncB		97	137	50	36	127	544		Nicotinate phosphoribosyltransferase pncB2
Mfumv2_1074	1464	atpD	*	334	449	376	191	212	824	*	F1 sector of membrane-bound ATP synthase, beta subunit
Mfumv2_1075	408	atpC	*	136	59	129	21	164	206	*	F0F1-type ATP synthase, epsilon subunit
Mfumv2_1076	279		*	107	36	74	11	70	62	*	F0F1-type ATP synthase subunit, ATPase_gene1 family
Mfumv2_1077	657	atpB	*	78	55	57	16	84	180	*	ATP synthase subunit a 1
Mfumv2_1078	267	atpE	*	76	23	42	3	89	65	*	ATP synthase subunit c 1
Mfumv2_1079	747	atpF	*	132	121	223	70	251	497	*	ATP synthase subunit b
Mfumv2_1080	1515	atpA	*	98	191	109	59	192	768	*	F1 sector of membrane-bound ATP synthase, alpha subunit
Mfumv2_1081	885	atpG	*	137	152	257	54	238	504	*	F0F1-type ATP synthase, gamma subunit
Mfumv2_1082	1548	pckA	*	90	150	106	59	219	987	*	Phosphoenolpyruvate carboxykinase [ATP]
Mfumv2_1083	1029	rfaG		59	48	31	10	33	105		Glycosyltransferase
Mfumv2_1084	2202	gdb	*	89	209	81	61	213	1451	*	Glycogen debranching enzyme
Mfumv2_1085	1245	aceA	*	116	152	122	57	146	494	*	isocitrate lyase
Mfumv2_1086	1545	aceB	*	134	255	151	75	203	764	*	Malate synthase
Mfumv2_1087	471			82	35	8	3	105	127		conserved membrane protein of unknown function
Mfumv2_1088	168			48	8	84	6	19	7		conserved protein of unknown function
Mfumv2_1089	1485	glcD		269	409	241	112	182	789		glycolate oxidase subunit, FAD-linked
Mfumv2_1090	1077	glcE		96	105	142	37	147	512		glycolate oxidase FAD binding subunit
Mfumv2_1091	1248	glcF		83	114	69	25	99	298		glycolate oxidase iron-sulfur subunit
Mfumv2_1092	891			631	544	376	184	633	1502		Opacity protein or related surface antigen
Mfumv2_1093	1293			84	131	61	45	101	337		Membrane fusion component of tripartite multidrug resistance system
Mfumv2_1094	1503	tolC		64	104	48	38	90	348		Outer membrane component of tripartite multidrug resistance system
Mfumv2_1095	1569	proP		100	168	53	36	87	443		Inner membrane component of tripartite multidrug resistance system
Mfumv2_1097	1530	proP		144	220	124	95	80	345		Inner membrane component of tripartite multidrug resistance system
Mfumv2_1098	2370		*	246	599	336	316	274	1943	*	putative phosphoketolase 2
Mfumv2_1099	132			0	0	0	0	11	4		conserved protein of unknown function
Mfumv2_1100	180			200	44	71	10	101	41		protein of unknown function
Mfumv2_1101	2574	topA	*	286	855	227	265	454	3101	*	DNA topoisomerase
Mfumv2_1102	951	smf		78	93	119	39	117	322		Rossmann fold nucleotide-binding protein involved in DNA uptake

Mfumv2_1103	1083	aroB	*	108	143	89	38	149	365	*	3-dehydroquinate synthase
Mfumv2_1104	3486	dnaE	*	205	823	148	210	207	1760	*	DNA polymerase III subunit alpha
Mfumv2_1105	1902	uvrD	*	107	212	70	74	188	1030	*	ATP-dependent DNA helicase UvrD/PcrA
Mfumv2_1106	984			264	240	238	102	382	1146		putative lipopolysaccharide transport protein B (ABC superfamily, atp_bind) (LptB) (Modular protein)
Mfumv2_1107	798	ywfI		268	220	155	49	428	1192		Predicted heme peroxidase involved in anaerobic stress response
Mfumv2_1108	273	grxC		287	75	316	33	351	276		Glutaredoxin
Mfumv2_1109	573	def		428	249	218	71	562	939		Peptide deformylase
Mfumv2_1110	423	exbD		128	64	62	17	104	120		Biopolymer transport protein ExbD/TolR
Mfumv2_1111	675	tolQ		334	221	162	68	246	509		MotA/TolQ/ExbB proton channel family protein
Mfumv2_1112	1218			276	347	206	122	123	465		conserved protein of unknown function
Mfumv2_1113	228			1134	305	1812	124	1988	1383		PurA family ssDNA and RNA-binding protein
Mfumv2_1114	1077	ispA		431	545	636	265	1108	2931		Geranylgeranyl pyrophosphate synthase
Mfumv2_1115	387	rnpA		91	49	92	17	410	354		Ribonuclease P protein component
Mfumv2_1116	267			142	44	158	27	241	145		putative membrane protein insertion efficiency factor
Mfumv2_1117	1776	yidC		143	274	128	97	232	1081		Membrane protein insertase YidC
Mfumv2_1118	1575	nadB	*	182	344	164	116	447	1905	*	L-aspartate oxidase
Mfumv2_1119	408			127	60	132	17	479	411		Predicted transcriptional regulator
Mfumv2_1120	984	sufI		508	653	8	6	242	525		Multicopper oxidase
Mfumv2_1121	420	cccA	*	294	157	29	7	48	64	*	Cytochrome c family protein
Mfumv2_1122	699		*	137	113	19	4	30	50	*	Cytochrome c, class I
Mfumv2_1123	1080	sufI		327	341	11	9	28	75		Multicopper oxidase
Mfumv2_1124	1749	arnT		94	171	73	44	97	491		4-amino-4-deoxy-L-arabinose transferase or related glycosyltransferase of PMT family
Mfumv2_1127	210			7525	1665	10912	632	11634	4062		protein of unknown function
Mfumv2_1132	216			103	26	114	10	32	20		conserved protein of unknown function
Mfumv2_1133	525			145	78	157	26	107	162		conserved protein of unknown function
Mfumv2_1134	492			162	76	195	33	232	402		conserved membrane protein of unknown function
Mfumv2_1135	1446	icd	*	287	452	366	164	284	1035	*	Isocitrate dehydrogenase [NADP]
Mfumv2_1136	885	iolE		162	144	100	38	180	450		Sugar phosphate isomerase/epimerase
Mfumv2_1137	429			496	208	287	67	871	1412		conserved exported protein of unknown function
Mfumv2_1138	1695	malQ		89	183	59	46	118	549		4-alpha-glucanotransferase
Mfumv2_1139	1107			53	77	21	16	79	263		conserved protein of unknown function
Mfumv2_1140	309			41	13	31	5	51	45		Helix-turn-helix transcription regulator MerD
Mfumv2_1141	939	cbpA		331	336	230	115	223	467		Curved DNA-binding protein
Mfumv2_1142	897	alkA		94	91	106	29	137	343		3-methyladenine DNA glycosylase/8-oxoguanine DNA glycosylase
Mfumv2_1143	162			216	34	271	14	385	202		conserved protein of unknown function
Mfumv2_1144	138			0	0	0	0	20	7		protein of unknown function
Mfumv2_1145	291			26	0	74	0	8	0		conserved protein of unknown function
Mfumv2_1146	138			0	0	0	0	12	5		conserved protein of unknown function
Mfumv2_1147	141			17	2	9	1	3	2		conserved protein of unknown function
Mfumv2_1148	1398	argD		279	463	271	198	627	2581		Ornithine/acetylornithine aminotransferase
Mfumv2_1149	636	gloB		153	103	89	26	260	468		Zn-dependent hydrolase, glyoxylase family
Mfumv2_1150	1602	bglB		162	283	92	86	242	1069		Beta-glucosidase/6-phospho-beta-glucosidase/beta-galactosidase
Mfumv2_1151	1182	murG		193	281	163	101	299	986		UDP-N-acetylglucosamine:LPS N-acetylglucosamine transferase
Mfumv2_1152	507			128	64	39	13	292	470		conserved exported protein of unknown function
Mfumv2_1153	531			73	56	95	27	229	298		conserved protein of unknown function
Mfumv2_1154	789	kdsA		166	176	123	44	255	505		2-dehydro-3-deoxyphosphooctonate aldolase
Mfumv2_1155	1614	pyrG		288	514	221	165	278	1235		CTP synthetase
Mfumv2_1156	765	kdsB		175	120	90	30	193	413		3-deoxy-manno-octulosonate cytidylyltransferase
Mfumv2_1157	540			53	25	84	21	121	163		D,D-heptose 1,7-bisphosphate phosphatase
Mfumv2_1158	1020	rfaE		240	243	370	141	257	619		D-glycero-beta-D-manno-heptose 7-phosphate kinase
Mfumv2_1159	864	pheA	*	136	112	31	12	90	204	*	Prephenate dehydratase
Mfumv2_1160	537	slp		102	56	93	23	119	214		Starvation-inducible outer membrane lipoprotein
Mfumv2_1161	870			132	110	59	21	377	919		Predicted amidohydrolase
Mfumv2_1162	1068		*	101	128	69	44	279	904	*	Peptidylarginine deiminase or related enzyme
Mfumv2_1163	552			145	76	142	35	204	312		conserved membrane protein of unknown function
Mfumv2_1164	957	rfaF		37	48	40	19	151	318		ADP-heptose:LPS heptosyltransferase

Mfumv2_1165	1155	nifS		410	522	377	174	578	1961		Cysteine desulfurase
Mfumv2_1166	1173			225	265	115	80	243	763		Selenocysteine lyase/Cysteine desulfurase (Modular protein)
Mfumv2_1167	792	hisK	*	156	129	93	39	288	577	*	putative histidinol-phosphatase
Mfumv2_1168	624	mobA		65	40	37	9	129	231		putative molybdenum cofactor guanylyltransferase
Mfumv2_1169	531	mobB		153	84	221	43	164	222		Molybdopterin-guanine dinucleotide biosynthesis protein
Mfumv2_1170	1221	moeA		127	193	101	51	266	954		Molybdopterin biosynthesis enzyme
Mfumv2_1171	915			78	78	55	24	121	317		Alpha/beta superfamily hydrolase (Modular protein)
Mfumv2_1172	651		*	107	81	49	19	97	150	*	Cytochrome c family protein
Mfumv2_1173	426			44	18	29	7	67	75		conserved membrane protein of unknown function
Mfumv2_1174	474	bcp		138	71	58	19	299	480		Peroxiredoxin
Mfumv2_1175	423			697	295	405	76	378	454		conserved exported protein of unknown function
Mfumv2_1176	1551			592	858	279	203	185	833		conserved exported protein of unknown function
Mfumv2_1177	852	fdhD		139	135	146	45	172	419		Protein FdhD homolog
Mfumv2_1178	786	modA		177	134	159	43	92	226		ABC-type molybdate transport system, periplasmic component
Mfumv2_1179	678	modB		64	36	35	11	58	111		Molybdenum transport system permease protein ModB
Mfumv2_1180	1146	malK		42	46	26	14	59	202		ABC-type molybdate transport system, ATPase component
Mfumv2_1181	618			29	15	4	2	79	147		HAD superfamily hydrolase
Mfumv2_1182	1191	proP		63	84	52	29	68	250		Permease of the major facilitator superfamily
Mfumv2_1183	1836	xoxF		6220	12935	5291	4296	6041	29875		methanol dehydrogenase XoxF
Mfumv2_1184	873	xoxJ		714	709	1057	339	1478	2869		XoxJ periplasmic substrate binding protein
Mfumv2_1185	879	xoxGJ		611	725	829	323	1042	2162		Cytochrome c1 family protein involved in methanol oxydation [XoxG/XoxJ fusion]
Mfumv2_1186	369			3008	1014	1655	282	1871	1847		putative pterin-4-alpha-carbinolamine dehydratase
Mfumv2_1188	582			27	13	175	14	19	9		conserved protein of unknown function
Mfumv2_1189	282			125	33	527	35	48	27		conserved protein of unknown function
Mfumv2_1189a	771			256	209	878	189	155	228		hypothetical protein (Not annotated in Genoscope)
Mfumv2_1191	897	mmsB	*	2968	2711	2914	985	2984	8108	*	3-hydroxyisobutyrate dehydrogenase or related beta-hydroxyacid dehydrogenase
Mfumv2_1192	1386	lpd	*	362	657	338	208	525	1888	*	Dihydrolipoyl dehydrogenase
Mfumv2_1193	1236	pdhC	*	625	853	970	443	1061	3340	*	Dihydrolipoyllysine-residue acetyltransferase component of pyruvate dehydrogenase complex
Mfumv2_1195	1701	nrfG		95	177	74	53	151	637		TPR repeats containing protein
Mfumv2_1196	978	acoB	*	559	603	600	227	853	2380	*	Pyruvate/2-oxoglutarate dehydrogenase complex,beta subunit
Mfumv2_1197	1074	pdhA	*	2146	2197	1957	752	928	2866	*	Pyruvate dehydrogenase E1 component subunit alpha
Mfumv2_1198	1365	gabT	*	90	134	96	39	83	300	*	4-aminobutyrate aminotransferase or related aminotransferase
Mfumv2_1199	198			91	17	105	7	337	220		conserved protein of unknown function
Mfumv2_1200	138			3	1	49	2	22	4		protein of unknown function
Mfumv2_1201	291			38	0	119	0	9	0		conserved protein of unknown function
Mfumv2_1202	279			38	0	0	0	21	1		conserved protein of unknown function
Mfumv2_1203	327			66	23	124	14	126	79		conserved protein of unknown function
Mfumv2_1204	306			138	40	88	16	187	175		conserved protein of unknown function
Mfumv2_1205	567			548	307	533	145	1219	2359		Integral membrane protein CcmA involved in cell shape determination
Mfumv2_1206	900			884	980	734	316	1460	3092		conserved protein of unknown function
Mfumv2_1207	204			862	210	598	55	2487	1721		conserved protein of unknown function
Mfumv2_1208	264	rpmB		894	285	510	65	1911	1760		50S ribosomal protein L28
Mfumv2_1209	252	rpsR	*	1294	374	712	94	1752	1559	*	30S ribosomal protein S18
Mfumv2_1210	684	dksA		1162	733	574	199	1622	3071		DnaK suppressor protein
Mfumv2_1211	1644	pgi	*	114	194	82	66	170	868	*	glucosephosphate isomerase
Mfumv2_1212	195			4	1	53	3	8	2		conserved protein of unknown function
Mfumv2_1213	288			61	17	133	9	6	3		conserved protein of unknown function
Mfumv2_1214	2838	treY		201	657	145	210	223	1788		Maltooligosyl trehalose synthase
Mfumv2_1215	219			629	144	278	34	265	151		conserved protein of unknown function
Mfumv2_1216	450			36	16	171	21	52	32		protein of unknown function
Mfumv2_1217	417			519	162	393	53	222	108		transposase
Mfumv2_1218	1845	treZ	*	87	208	66	67	186	872	*	Malto-oligosyltrehalose trehalohydrolase
Mfumv2_1219	3408			193	780	254	354	183	1434		Trehalose synthase
Mfumv2_1221	1395	zraR		65	102	59	28	100	415		Transcriptional regulatory protein ZraR
Mfumv2_1222	1446			73	122	47	24	117	466		Signal transduction histidine kinase
Mfumv2_1223	1965	glgB	*	107	243	88	92	175	876	*	1,4-alpha-glucan branching enzyme

Mfumv2_1225	1314	mntH		76	111	50	20	99	349		manganese/divalent cation transporter
Mfumv2_1226	714	troR		360	237	138	71	275	542		Mn-dependent transcriptional regulator MntR
Mfumv2_1227	2745	acnA	*	258	753	155	176	322	2495	*	aconitate hydratase 1
Mfumv2_1228	579			86	53	27	9	83	119		Alpha/beta hydrolase superfamily protein
Mfumv2_1229	1638	oppA		42	76	44	29	101	382		ABC-type oligopeptide transport system,periplasmic component
Mfumv2_1230	321	secG		249	86	112	23	302	222		Preprotein translocase subunit SecG
Mfumv2_1231	768	tpiA	*	349	309	155	63	542	1181	*	Triosephosphate isomerase
Mfumv2_1232	1215	pgk	*	349	511	157	109	876	2923	*	phosphoglycerate kinase
Mfumv2_1233	1044	gapA	*	816	872	548	192	1497	4753	*	glyceraldehyde-3-phosphate dehydrogenase A
Mfumv2_1234	1077	msrA		104	97	11	9	112	353		Methionine sulfoxide reductase with associated domain
Mfumv2_1235	675			74	55	6	3	98	140		conserved protein of unknown function
Mfumv2_1236	315	cutA		242	72	195	18	326	374		Periplasmic divalent cation tolerance protein cutA
Mfumv2_1237	450			66	27	202	26	19	15		conserved protein of unknown function
Mfumv2_1238	1107	mnmA	*	375	520	528	198	332	887	*	tRNA-specific 2-thiouridylase MnmA 2
Mfumv2_1239	1239	clpX		523	798	371	210	747	2639		ATPase and specificity subunit of ClpX-ClpP ATP-dependent serine protease
Mfumv2_1240	651	clpP		998	677	874	271	1072	1844		ATP-dependent Clp protease proteolytic subunit (Endopeptidase Clp) (Heat shock protein F21.5)
Mfumv2_1241	1332	tig		501	698	804	343	380	1302		Trigger factor
Mfumv2_1242	195			490	95	477	40	147	79		conserved protein of unknown function
Mfumv2_1243	933	trxB		562	526	453	152	384	1075		Thioredoxin reductase
Mfumv2_1244	1326			322	423	324	159	328	1051		conserved protein of unknown function
Mfumv2_1245	1185	sucC	*	188	275	251	178	367	1074	*	succinyl-CoA synthetase, beta subunit
Mfumv2_1246	891	sucD	*	331	402	766	240	753	1920	*	succinyl-CoA synthetase, NAD(P)-binding, alpha subunit
Mfumv2_1247	2073	aroA	*	347	862	629	387	520	2595	*	3-phosphoshikimate 1-carboxyvinyltransferase
Mfumv2_1248	282			1807	470	646	92	603	530		conserved protein of unknown function
Mfumv2_1249	195			176	35	465	28	277	138		conserved protein of unknown function
Mfumv2_1250	441			36	14	119	15	16	10		conserved protein of unknown function
Mfumv2_1251	195			36	5	116	6	5	2		conserved protein of unknown function
Mfumv2_1252	648	plsC		42	30	67	13	116	153		1-acyl-sn-glycerol-3-phosphate acyltransferase
Mfumv2_1253	279	feoA		616	205	614	56	493	326		Fe2+ transport system protein A
Mfumv2_1254	138			84	13	9	1	120	48		protein of unknown function
Mfumv2_1255	2118	feoB		192	487	189	134	229	1312		Ferrous iron transport protein B
Mfumv2_1256	1677	rpsA	*	2205	4018	2638	1862	2092	9380	*	30S ribosomal protein S1
Mfumv2_1257	255			156	46	229	23	30	23		conserved protein of unknown function
Mfumv2_1258	156			303	51	591	30	35	12		conserved protein of unknown function
Mfumv2_1259	483	cyoA	*	257	126	304	51	90	130	*	Heme/copper-type cytochrome oxidase, subunit 2
Mfumv2_1260	1686	cbaA	*	203	370	119	101	120	627	*	Cytochrome c oxidase subunit 1
Mfumv2_1261	150			9	2	0	0	43	27		conserved protein of unknown function
Mfumv2_1262	1053			211	233	119	73	363	1217		conserved exported protein of unknown function
Mfumv2_1263	306			19	6	15	1	4	3		protein of unknown function
Mfumv2_1264	294			13	4	78	7	3	2		conserved protein of unknown function
Mfumv2_1265	225			12	0	50	0	3	0		conserved protein of unknown function
Mfumv2_1266	174			125	17	405	23	17	5		conserved protein of unknown function
Mfumv2_1267	117			1282	112	479	27	138	55		protein of unknown function
Mfumv2_1268	783			75	53	36	10	85	223		putative ROK family protein
Mfumv2_1269	183			63	15	28	4	89	36		conserved exported protein of unknown function
Mfumv2_1270	1263			122	164	62	49	230	828		L-sorbosone dehydrogenase
Mfumv2_1271	444			638	289	227	61	308	365		conserved protein of unknown function
Mfumv2_1272	531	sseA		107	75	224	32	95	117		Rhodanese-related sulfurtransferase
Mfumv2_1273	330			533	164	206	27	572	620		conserved exported protein of unknown function
Mfumv2_1274	279			1127	318	838	87	847	753		conserved protein of unknown function
Mfumv2_1275	1533	amtB		294	449	1082	411	391	1416		Ammonia channel
Mfumv2_1276	654			1506	432	1165	134	392	384		Ribonucleoside-diphosphate reductase (fragment)
Mfumv2_1277	708			62	19	40	4	38	24		ribonucleoside diphosphate reductase, alpha subunit (fragment)
Mfumv2_1278	153			130	13	33	4	88	41		conserved protein of unknown function
Mfumv2_1279	414			586	267	874	163	825	1125		Hemoglobin-like flavoprotein fused to Roadblock/LC7 domain
Mfumv2_1280	453			39	12	33	5	19	22		Ammonium transporter (fragment)

Mfumv2_1281	2832	nrdA		834	1883	482	424	415	2172		Ribonucleoside-diphosphate reductase subunit alpha
Mfumv2_1282	1056	nrdB		481	667	215	118	444	1263		Ribonucleoside-diphosphate reductase subunit beta
Mfumv2_1283	1287	lysA	*	404	668	324	164	508	2050	*	Diaminopimelate decarboxylase
Mfumv2_1284	531	pncA		412	254	535	94	392	453		Amidase
Mfumv2_1285	381	glnK		371	150	125	28	193	184		Nitrogen regulatory protein PII
Mfumv2_1286	429			158	63	170	26	172	241		conserved protein of unknown function
Mfumv2_1287	915	rbsK		60	64	111	32	135	306		Sugar kinase, ribokinase family
Mfumv2_1288	243			927	224	281	23	344	215		conserved protein of unknown function
Mfumv2_1289	444	osmC		136	69	82	15	239	311		Organic hydroperoxide reductase
Mfumv2_1290	363	rlpA		120	41	88	14	203	247		Lipoprotein
Mfumv2_1291	660	smtA		65	52	167	32	117	191		SAM-dependent methyltransferase
Mfumv2_1292	804	hmp		184	131	118	44	143	318		Hemoglobin-like flavoprotein fused to Roadblock/LC7 domain
Mfumv2_1293	2424	cirA		42	94	51	55	17	125		Outer membrane receptor protein, mostly Fe transport
Mfumv2_1294	1302	nasA		67	93	321	143	23	80		Nitrate transporter
Mfumv2_1295	360	nirD		63	24	308	38	33	33		Ferredoxin subunit of nitrite reductase
Mfumv2_1296	2442	nasD		140	382	854	720	19	107		Nitrite reductase [NAD(P)H]
Mfumv2_1297	2151	nasC		20	52	105	67	13	77		Assimilatory nitrate reductase catalytic subunit
Mfumv2_1299	1083	tauA		28	25	41	10	34	90		Nitrate ABC transporter, nitrate-binding protein
Mfumv2_1300	924	lysR	*	127	108	69	30	36	105	*	RuBisCO operon transcriptional regulator CbbR
Mfumv2_1301	843			244	232	221	71	263	731		(2R)-phospho-3-sulfolactate synthase
Mfumv2_1302	504			95	57	97	23	135	140		conserved exported protein of unknown function
Mfumv2_1303	579			168	81	178	48	229	345		conserved exported protein of unknown function
Mfumv2_1305	678	tonB		171	99	66	20	489	889		Periplasmic protein TonB
Mfumv2_1306	1437			175	276	119	83	203	903		Glycosyl hydrolase, family 57
Mfumv2_1307	1428			57	75	45	28	489	2807		conserved exported protein of unknown function
Mfumv2_1310	1020	smtA		334	387	257	124	261	736		SAM-dependent methyltransferase
Mfumv2_1311	300			283	82	720	70	163	71		protein of unknown function
Mfumv2_1312	144			19	2	0	0	14	4		conserved protein of unknown function
Mfumv2_1313	357			47	19	191	28	33	21		protein of unknown function
Mfumv2_1314	429			29	12	52	15	18	12		conserved exported protein of unknown function
Mfumv2_1315	180			100	18	96	11	11	4		conserved protein of unknown function
Mfumv2_1316	654	upp		95	69	107	26	139	221		Uracil phosphoribosyltransferase
Mfumv2_1317	1011	hemB		321	328	194	88	223	529		Delta-aminolevulinic acid dehydratase
Mfumv2_1318	2577	glnE		122	420	127	114	207	1233		Glutamine synthetase adenylyltransferase
Mfumv2_1319	729	rnc		66	53	48	15	106	198		Ribonuclease 3
Mfumv2_1320	924	xerD		163	145	88	29	213	540		Tyrosine recombinase XerC
Mfumv2_1321	705	gcd		114	94	143	31	191	310		Nucleoside-diphosphate-sugar pyrophosphorylase
Mfumv2_1322	591			72	43	51	12	89	174		Non-canonical purine NTP pyrophosphatase
Mfumv2_1323	393			76	29	25	5	8	5		conserved protein of unknown function
Mfumv2_1324	153			0	0	45	2	0	0		conserved protein of unknown function
Mfumv2_1325	117			199	19	80	4	25	11		protein of unknown function
Mfumv2_1326	174			119	20	37	5	10	3		conserved protein of unknown function
Mfumv2_1327	225			11	0	25	0	0	0		conserved protein of unknown function
Mfumv2_1328	294			44	11	68	4	13	3		conserved protein of unknown function
Mfumv2_1329	279			15	4	0	0	3	4		conserved protein of unknown function
Mfumv2_1330	168			10	2	66	2	24	8		protein of unknown function
Mfumv2_1331	1086	tal	*	141	148	100	50	243	931	*	Transaldolase
Mfumv2_1332	705		*	467	337	146	50	329	731	*	Zn-ribbon protein, possibly nucleic acid-binding
Mfumv2_1333	282	rpoZ	*	2680	684	2221	286	4795	5099	*	DNA-directed RNA polymerase, subunit K/omega
Mfumv2_1334	459	smpB		977	509	906	163	790	1023		SsrA-binding protein
Mfumv2_1335	531			36	24	109	14	30	42		conserved exported protein of unknown function
Mfumv2_1336	162			289	42	519	33	64	18		conserved protein of unknown function
Mfumv2_1337	318			322	76	4	1	12	5		protein of unknown function
Mfumv2_1338	411			14	4	0	0	10	5		protein of unknown function
Mfumv2_1339	291			227	59	271	29	79	43		conserved protein of unknown function
Mfumv2_1340	1533	purH		252	424	231	157	329	1451		Bifunctional purine biosynthesis protein PurH [Phosphoribosylaminoimidazolecarboxamide formyltransferase ; IMP cyclohydrolase]

Mfumv2_1341	714			696	501	298	105	837	2099		Metal-dependent phosphoesterase (PHP family)
Mfumv2_1342	711	ubiE		123	94	174	42	213	318		Demethylmenaquinone methyltransferase
Mfumv2_1343	546			324	195	271	72	562	768		conserved protein of unknown function
Mfumv2_1344	468	sufE		432	261	342	74	732	703		Cysteine desulfurase SufE subunit
Mfumv2_1345	855	sseA		1049	1027	672	321	1032	2322		putative thiosulfate sulfurtransferase SseA
Mfumv2_1346	951	bioB		235	261	216	81	374	1045		Biotin synthase
Mfumv2_1347	213			17	2	0	0	25	16		protein of unknown function
Mfumv2_1348	1455			51	80	65	32	57	205		Signal transduction histidine kinase
Mfumv2_1349	1335	atoC		98	148	84	36	103	351		DNA-binding response regulator, NtrC family (Contains REC, AAA-type ATPase, and DNA-binding Fis domains)
Mfumv2_1350	816			92	66	27	8	52	120		conserved protein of unknown function
Mfumv2_1351	336	trxA		1516	498	781	117	447	406		Thioredoxin
Mfumv2_1352	858			412	377	258	99	550	1449		conserved protein of unknown function
Mfumv2_1353	1914	ftsH	*	225	547	209	186	225	1009	*	ATP-dependent zinc metalloprotease FtsH 1
Mfumv2_1354	825			129	151	117	31	211	415		Uracil-DNA glycosylase
Mfumv2_1355	801	rluD	*	324	313	266	88	572	865	*	Ribosomal large subunit pseudouridine synthase D
Mfumv2_1357	276	gloB		6767	2184	3945	496	3746	3267		Zn-dependent hydrolase, glyoxylase family
Mfumv2_1358	645	bioD		188	120	135	38	172	278		ATP-dependent dethiobiotin synthetase BioD
Mfumv2_1359	1428			320	487	221	171	213	827		conserved protein of unknown function
Mfumv2_1360	651	pdxH		111	76	113	35	197	326		Pyridoxine/pyridoxamine 5'-phosphate oxidase
Mfumv2_1361	474	cycA	*	2539	1237	2168	483	1663	2020	*	Cytochrome c-552
Mfumv2_1364	261			102	24	142	8	112	81		conserved protein of unknown function
Mfumv2_1365	216			89	22	250	12	96	46		conserved protein of unknown function
Mfumv2_1366	1482			1666	1136	2349	765	311	263		conserved protein of unknown function
Mfumv2_1367	1191			0	0	0	0	2	0		protein of unknown function
Mfumv2_1368	417			446	113	1016	75	203	42		transposase
Mfumv2_1369	744			25	21	54	9	41	69		conserved exported protein of unknown function
Mfumv2_1370	657			47	26	22	8	35	73		conserved exported protein of unknown function
Mfumv2_1371	660			9	8	6	3	19	33		conserved protein of unknown function
Mfumv2_1372	576			34	20	29	7	22	32		conserved exported protein of unknown function
Mfumv2_1373	630			15	13	6	3	18	39		conserved protein of unknown function
Mfumv2_1374	399			81	29	10	3	28	27		conserved protein of unknown function
Mfumv2_1375	558			26	15	2	1	17	29		conserved membrane protein of unknown function
Mfumv2_1376	903			72	60	29	11	38	93		conserved membrane protein of unknown function
Mfumv2_1377	174			55	10	15	2	209	107		conserved protein of unknown function
Mfumv2_1378	984	rpoD	*	53	48	28	13	54	146	*	DNA-directed RNA polymerase, sigma subunit (Sigma70/sigma32)
Mfumv2_1379	1158			9	12	1	1	13	29		conserved protein of unknown function
Mfumv2_1380	246			16	3	10	2	11	9		conserved protein of unknown function
Mfumv2_1381	918	mxoR		31	29	26	12	32	88		ATPase family associated with various cellular activities (AAA) pfam07728
Mfumv2_1382	1155			29	36	18	8	25	76		conserved protein of unknown function
Mfumv2_1383	1101			52	39	17	11	43	85		conserved protein of unknown function
Mfumv2_1384	423			13	6	20	4	4	2		conserved protein of unknown function
Mfumv2_1386	1869			57	111	40	36	63	375		conserved exported protein of unknown function
Mfumv2_1387	633			27	15	7	1	24	35		conserved protein of unknown function
Mfumv2_1388	249			3	1	23	2	34	31		conserved exported protein of unknown function
Mfumv2_1389	393			10	6	0	0	16	12		conserved membrane protein of unknown function
Mfumv2_1390	2130	virD		12	25	6	8	21	110		Type IV secretory pathway, VirD4 component
Mfumv2_1391	660			14	8	4	2	6	14		conserved protein of unknown function
Mfumv2_1392	165			257	36	119	7	126	82		protein of unknown function
Mfumv2_1393	714			32	21	11	6	33	71		conserved protein of unknown function
Mfumv2_1394	597			28	16	30	9	77	129		conserved protein of unknown function
Mfumv2_1395	702			79	58	20	5	104	240		conserved protein of unknown function
Mfumv2_1396	927			22	17	19	6	38	101		conserved protein of unknown function
Mfumv2_1397	2058	pulE		14	29	2	3	20	97		Type II secretory pathway, ATPase PulE/Tfp pilus assembly pathway, ATPase PilB
Mfumv2_1398	789			1	1	0	0	6	17		conserved protein of unknown function
Mfumv2_1399	972			12	8	5	4	8	22		conserved exported protein of unknown function
Mfumv2_1400	1203	virB		17	18	1	1	24	72		Type IV secretory pathway, VirB10 component

Mfumv2_1401	903			35	27	3	2	30	96		conserved protein of unknown function
Mfumv2_1402	1596	spo0J		44	73	34	21	92	513		Chromosome (Plasmid) partitioning protein,ParB/Spo0J, contains ParB-like nuclease domain
Mfumv2_1403	978		*	28	24	38	9	49	164	*	DNA-directed RNA polymerase, sigma subunit (Sigma70/sigma32)
Mfumv2_1404	1368			66	83	102	40	33	139		conserved membrane protein of unknown function
Mfumv2_1405	765			45	36	25	6	60	116		conserved exported protein of unknown function
Mfumv2_1406	795			26	19	25	9	58	130		conserved exported protein of unknown function
Mfumv2_1407	345			12	7	47	6	22	29		conserved membrane protein of unknown function
Mfumv2_1409	468			64	29	14	5	35	49		conserved membrane protein of unknown function
Mfumv2_1410	408			19	6	3	1	23	29		conserved protein of unknown function
Mfumv2_1411	2664	virB		30	86	19	23	51	359		Type IV secretory pathway, VirB4 component
Mfumv2_1412	2478			25	71	18	16	27	198		conserved exported protein of unknown function
Mfumv2_1413	501	ompA		71	39	70	12	47	71		Outer membrane protein or related peptidoglycan-associated (Lipo)protein
Mfumv2_1415	783			79	50	48	9	98	229		SAM-dependent methyltransferase (Modular protein)
Mfumv2_1416	660			1042	681	1482	306	369	675		conserved exported protein of unknown function
Mfumv2_1417	1392	lpd		151	242	141	76	201	708		Dihydrolipoyl dehydrogenase
Mfumv2_1418	150			0	0	0	0	0	0		conserved protein of unknown function
Mfumv2_1419	345	glnB		943	302	719	99	883	998		regulatory protein P-II for glutamine synthetase
Mfumv2_1420	1434	glnA		746	1217	893	575	2065	7047		glutamine synthetase
Mfumv2_1423	1203	rfaG		97	103	39	22	114	403		Glycosyltransferase
Mfumv2_1424	768	pgpB		55	51	34	13	84	180		Membrane-associated phospholipid phosphatase
Mfumv2_1425	1203	nifS		146	217	129	67	194	578		Cysteine sulfinate desulfinase/cysteine desulfurase or related enzyme
Mfumv2_1426	798			234	221	114	54	198	464		conserved membrane protein of unknown function
Mfumv2_1427	354			530	196	446	59	1240	1568		conserved protein of unknown function
Mfumv2_1428	501	nrdR		697	388	465	94	290	410		Transcriptional repressor NrdR
Mfumv2_1429	453			120	56	54	9	202	279		Predicted metal-binding, possibly nucleic acid-binding protein
Mfumv2_1430	1044	plsX		202	244	220	101	541	1464		Phosphate acyltransferase
Mfumv2_1431	1074	fabH		500	606	282	158	533	1447		3-oxoacyl-[acyl-carrier-protein] synthase III
Mfumv2_1432	1089	frvX		79	100	59	31	113	362		Cellulase M or related protein
Mfumv2_1433	1455	purF		431	705	304	175	638	3037		Amidophosphoribosyltransferase
Mfumv2_1434	1014	purM		149	177	90	49	174	480		Phosphoribosylformylglycinamidine cyclo-ligase
Mfumv2_1435	186			7	2	56	3	36	9		protein of unknown function
Mfumv2_1436	138			6	1	66	2	9	3		conserved protein of unknown function
Mfumv2_1437	141			0	0	0	0	26	0		conserved protein of unknown function
Mfumv2_1438	180			0	0	0	0	4	1		conserved protein of unknown function
Mfumv2_1439	180			7	2	31	1	5	2		conserved protein of unknown function
Mfumv2_1440	138			0	0	0	0	2	1		conserved protein of unknown function
Mfumv2_1441	243			8	2	0	0	0	0		protein of unknown function
Mfumv2_1442	117			0	0	0	0	0	0		tmRNA Mfumv2_1442 is a pseudogene)
Mfumv2_1443	243			0	0	0	0	0	0		tmRNA Mfumv2_1443 is a pseudogene)
Mfumv2_1444	237			7	0	42	0	5	0		protein of unknown function
Mfumv2_1445	483			57	0	29	0	11	0		conserved protein of unknown function
Mfumv2_1446	132			0	0	0	0	11	2		protein of unknown function
Mfumv2_1447	120			94	9	75	2	25	4		conserved protein of unknown function
Mfumv2_1448	312			34	9	22	2	9	10		conserved protein of unknown function
Mfumv2_1449	585			420	281	570	164	247	405		Hemoglobin-like protein
Mfumv2_1450	594	mcrA	*	278	180	122	25	433	911	*	Restriction endonuclease, McrA/HNH family
Mfumv2_1451	2805			163	523	123	186	200	1499		Predicted exporter of the RND superfamily
Mfumv2_1452	648	erfK		118	79	132	30	146	230		Uncharacterized erfK/srfK family protein,probably secreted
Mfumv2_1453	336			167	56	101	18	58	50		conserved membrane protein of unknown function
Mfumv2_1455	687			168	126	326	86	625	1024		putative 3-methyladenine DNA glycosylase
Mfumv2_1456	240	fdsD		593	155	979	112	588	298		Formate dehydrogenase delta subunit
Mfumv2_1457	2973	fdh2A		568	1947	665	845	1342	9413		NAD-dependent formate dehydrogenase, molybdenum containing, alpha subunit
Mfumv2_1458	1569	fdsB		569	802	435	248	1149	3882		Formate dehydrogenase beta subunit
Mfumv2_1459	459	nuoE		475	201	240	50	672	605		NAD-dependent formate dehydrogenase gamma subunit
Mfumv2_1460	1233			35	44	12	8	169	723		conserved membrane protein of unknown function
Mfumv2_1461a	81	pqqA		2920	265	1919	57	2133	514		Coenzyme PQQ synthesis protein A

Mfumv2_1461	897	pqqB		1308	1128	588	247	620	1344		Coenzyme PQQ synthesis protein B
Mfumv2_1462	786	pqqC		1165	1055	560	244	622	1301		Pyrroloquinoline-quinone synthase
Mfumv2_1463	273	pqqD		451	146	153	25	249	135		Coenzyme PQQ synthesis protein D
Mfumv2_1464	1128	pqqE		747	1018	514	239	509	1593		Coenzyme PQQ synthesis protein E
Mfumv2_1465	204			6790	1386	3759	250	5495	4031		conserved exported protein of unknown function
Mfumv2_1466	3579			497	2040	628	982	441	4275		Indolepyruvate ferredoxin oxidoreductase, alpha and beta subunits
Mfumv2_1467	1374	glyQS	*	461	686	524	262	523	1943	*	Glycine--tRNA ligase
Mfumv2_1468	2439			70	170	31	35	72	479		conserved protein of unknown function
Mfumv2_1469	909	nfo	*	84	82	66	22	94	215	*	putative endonuclease 4
Mfumv2_1470	345			773	215	328	44	2135	2255		Glutaredoxin-related protein
Mfumv2_1471	2223			26	55	17	15	15	80		conserved protein of unknown function
Mfumv2_1472	261			27	1	0	0	0	0		protein of unknown function
Mfumv2_1473	207			14	3	0	0	4	1		protein of unknown function
Mfumv2_1474	357			58	0	37	0	2	0		conserved protein of unknown function
Mfumv2_1475	327			74	0	294	0	27	0		conserved protein of unknown function
Mfumv2_1476	240			23	2	5	1	18	5		conserved exported protein of unknown function
Mfumv2_1478	708			176	152	214	63	179	312		conserved protein of unknown function
Mfumv2_1479	468	FCA		365	196	647	105	538	846		Cytosine deaminase
Mfumv2_1480	189			4337	718	13986	945	1418	668		protein of unknown function
Mfumv2_1481	1239	thrC	*	361	465	169	98	375	1159	*	Threonine synthase
Mfumv2_1482	291			1006	348	742	119	1507	1441		conserved protein of unknown function
Mfumv2_1483	243	napF	*	540	169	314	28	698	520	*	Ferredoxin
Mfumv2_1484	324			309	125	348	37	864	938		conserved protein of unknown function
Mfumv2_1485	1842	greA	*	910	1837	838	570	862	4059	*	Transcription elongation factor GreA fused to uncharacterized associated domain
Mfumv2_1486	654			67	50	87	20	95	196		Transcriptional regulator ArsR family
Mfumv2_1487	1596	serA	*	793	1410	958	711	1026	4049	*	D-3-phosphoglycerate dehydrogenase
Mfumv2_1488	384			2996	1158	2074	378	1170	1253		conserved exported protein of unknown function
Mfumv2_1489	375			917	354	510	78	307	262		conserved protein of unknown function
Mfumv2_1490	705			212	150	257	75	312	534		Phosphatase/phosphohexomutase HAD superfamily
Mfumv2_1491	603	phoE	*	516	381	556	160	716	1036	*	Phosphoglycerate mutase, PhoE family
Mfumv2_1492	2040	tktB	*	2291	5848	2574	2266	2655	13274	*	transketolase 2, thiamin-binding
Mfumv2_1493	1023	udk	*	1125	1391	860	386	2616	6163	*	Phosphoribulokinase
Mfumv2_1494	951	cbbX	*	3224	3574	3294	1316	11114	23354	*	Protein CbbX
Mfumv2_1495	417	cbbS	*	7256	3330	3607	743	7955	8552	*	Ribulose bisphosphate carboxylase small chain
Mfumv2_1496	1464	cbbL	*	10312	15962	4553	3329	9150	38755	*	Ribulose bisphosphate carboxylase large chain
Mfumv2_1497	1344	mipB	*	473	543	254	134	320	1369	*	Transaldolase
Mfumv2_1498	333	rplU	*	727	242	690	74	4027	4196	*	50S ribosomal protein L21
Mfumv2_1499	258	rpmA	*	724	209	738	69	2208	1330	*	50S ribosomal protein L27
Mfumv2_1500	1095	obg		900	1015	1137	447	1215	2455		GTPase Obg
Mfumv2_1501	351			189	66	92	16	162	144		conserved membrane protein of unknown function
Mfumv2_1502	1710			163	194	227	127	175	559		transposase
Mfumv2_1504	444			11	4	10	1	14	15		conserved protein of unknown function
Mfumv2_1505	213			74	13	64	3	12	7		conserved protein of unknown function
Mfumv2_1506	231			466	33	479	21	10	2		conserved exported protein of unknown function
Mfumv2_1507	327			116	0	254	0	85	0		conserved protein of unknown function
Mfumv2_1508	357			16	0	18	0	4	0		conserved protein of unknown function
Mfumv2_1509	849			193	103	315	78	90	148		protein of unknown function
Mfumv2_1510	639	rnhB		421	320	721	208	567	884		Ribonuclease HII
Mfumv2_1511	345	rplS	*	723	282	888	142	1139	1143	*	50S ribosomal subunit protein L19
Mfumv2_1512	708	trmD		995	716	964	275	1612	2939		tRNA (guanine-1-)-methyltransferase
Mfumv2_1513	258	rpsP	*	2814	622	1256	171	2904	2585	*	30S ribosomal subunit protein S16
Mfumv2_1514	1314	ffh		451	699	802	373	460	1460		Signal recognition particle protein
Mfumv2_1515	1392			546	949	831	449	828	2448		putative proteasome component
Mfumv2_1516	768	pre		894	814	997	300	750	1458		20S proteasome, alpha subunit
Mfumv2_1517	837	pre		1052	1084	1437	360	1112	2283		20S proteasome, alpha subunit
Mfumv2_1518	198			6152	1291	5466	383	1582	622		conserved protein of unknown function

Mfumv2_1519	1548			1535	2647	1717	1043	1089	4599		putative proteasome component
Mfumv2_1520	1689	arc		1325	2370	1154	746	1181	5550		Proteasome-associated ATPase
Mfumv2_1521	735	pyrF		800	609	554	214	283	546		Orotidine 5'-phosphate decarboxylase
Mfumv2_1522	861	ispE		144	128	123	52	234	518		4-diphosphocytidyl-2-C-methyl-D-erythritol kinase
Mfumv2_1523	1185	hcaD		338	440	358	159	374	1217		NAD(FAD)-dependent dehydrogenase
Mfumv2_1524	690	queE		505	405	490	166	386	572		7-carboxy-7-deazaguanine synthase
Mfumv2_1525	747			1548	1082	1555	496	829	1542		conserved protein of unknown function
Mfumv2_1526	855			202	197	177	65	245	539		PDZ domain containing protein
Mfumv2_1527	300			527	174	657	61	220	154		conserved protein of unknown function
Mfumv2_1528	933			516	537	557	180	507	1296		Aldo/keto reductase family enzyme
Mfumv2_1529	363	ygcM	*	61	20	84	12	171	170	*	6-pyruvoyl tetrahydrobiopterin synthase (PTPS)
Mfumv2_1530	1461	lysS		132	220	113	74	276	1044		Lysine--tRNA ligase
Mfumv2_1531	576			177	104	23	8	387	680		conserved exported protein of unknown function
Mfumv2_1532	399			67	26	41	6	67	81		conserved protein of unknown function
Mfumv2_1533	135			0	0	0	0	0	0		conserved protein of unknown function
Mfumv2_1534	1620			424	457	998	458	258	465		conserved protein of unknown function
Mfumv2_1535	135			21	4	75	2	15	4		conserved protein of unknown function
Mfumv2_1536	126			10	2	0	0	20	4		conserved protein of unknown function
Mfumv2_1537	894			61	61	40	13	63	147		Methylase involved in ubiquinone/menaquinone biosynthesis (Modular protein)
Mfumv2_1538	195			2	1	0	0	5	4		conserved protein of unknown function
Mfumv2_1539	684			438	274	176	76	47	89		conserved protein of unknown function
Mfumv2_1540	366			136	39	30	6	11	17		conserved protein of unknown function
Mfumv2_1541	243			127	10	74	6	107	52		conserved protein of unknown function
Mfumv2_1542	366			43	13	0	0	36	27		conserved protein of unknown function
Mfumv2_1543	177			2	1	7	1	5	4		conserved protein of unknown function
Mfumv2_1544	159			0	0	0	0	9	2		conserved protein of unknown function
Mfumv2_1545	123			10	2	0	0	10	3		protein of unknown function
Mfumv2_1546	591			485	282	216	62	164	205		putative Repressor lexA
Mfumv2_1547	1266	dinP	*	369	454	191	101	178	501	*	Nucleotidyltransferase/DNA polymerase UmuC
Mfumv2_1549	381			197	76	296	37	47	33		transposase
Mfumv2_1550	129			117	15	153	7	15	6		protein of unknown function
Mfumv2_1551	186			507	93	1348	66	243	83		conserved protein of unknown function
Mfumv2_1552	1257	rfaG		99	130	62	35	100	302		Glycosyltransferase
Mfumv2_1553	456			12	6	18	4	41	49		Starvation-inducible outer membrane lipoprotein (Modular protein)
Mfumv2_1556	795			432	337	140	56	280	663		conserved protein of unknown function
Mfumv2_1557	1116			22	29	27	12	781	3430		Methylthioribose kinase
Mfumv2_1558	483	sixA		215	103	169	37	200	262		Phosphohistidine phosphatase SixA
Mfumv2_1559	288			45	15	63	9	29	26		conserved protein of unknown function
Mfumv2_1560	1890	zntA		63	112	37	29	68	356		Cation transport ATPase
Mfumv2_1561	1035			99	107	137	62	98	304		conserved exported protein of unknown function
Mfumv2_1562	714	hupH		457	406	410	138	93	248		Hydrogenase expression protein HupH
Mfumv2_1563	696	hupZ		645	541	420	145	80	192		Ni/Fe-hydrogenase B-type cytochrome subunit
Mfumv2_1564	1800	hupL		1461	3151	1854	1509	77	419		Ni/Fe-hydrogenase large subunit Group 1d
Mfumv2_1565	1119	hupS		2232	3316	2685	1152	119	467		Ni/Fe-hydrogenase small subunit Group 1d (with tat signal)
Mfumv2_1567	1197			738	805	863	303	1110	2166		Formate dehydrogenase
Mfumv2_1568	693	glpG		46	28	56	11	49	90		Membrane associated serine protease
Mfumv2_1570	1257			466	656	459	245	241	884		conserved protein of unknown function
Mfumv2_1571	330			35	14	17	1	43	27		conserved protein of unknown function
Mfumv2_1572	354			122	35	54	9	268	249		VanZ like family protein
Mfumv2_1573	414	gloA		118	47	112	17	77	69		Lactoylglutathione lyase or related enzyme
Mfumv2_1575	960	amyA		337	352	247	87	398	908		Glycosidase
Mfumv2_1576	1146			206	234	116	40	125	459		Alpha-amylase/alpha-mannosidase
Mfumv2_1577	1158	rfaG		628	735	688	302	150	520		Glycosyltransferase
Mfumv2_1578	1119	pucG	*	161	203	154	83	242	805	*	Purine catabolism protein PucG
Mfumv2_1579	405	arsC		280	98	331	63	322	445		Glutaredoxin family protein
Mfumv2_1580	942			230	246	335	105	327	721		conserved protein of unknown function

Mfumv2_1581	864			946	971	1864	531	805	1807		conserved protein of unknown function
Mfumv2_1582	234			3525	873	4884	531	694	376		conserved exported protein of unknown function
Mfumv2_1583	324			18540	5478	30561	4059	1646	1177		conserved protein of unknown function
Mfumv2_1584	120			27	3	0	0	41	16		protein of unknown function
Mfumv2_1585	2142	glgX	*	266	689	181	169	364	2184	*	Glycogen operon protein GlgX homolog
Mfumv2_1586	798	ubiE		56	49	42	12	225	603		Methylase involved in ubiquinone/menaquinone biosynthesis
Mfumv2_1587	1845	sga		137	287	131	107	203	1014		Glucoamylase or related glycosyl hydrolase
Mfumv2_1588	612	wrbA		385	214	155	46	286	500		putative conserved flavoprotein
Mfumv2_1589	711			18	13	2	1	5	12		conserved exported protein of unknown function
Mfumv2_1590	1155			32	7	58	3	65	44		conserved protein of unknown function
Mfumv2_1591	144			3	1	9	1	16	3		conserved protein of unknown function
Mfumv2_1592	201			69	11	82	7	37	20		conserved protein of unknown function
Mfumv2_1593	681			36	29	21	5	22	37		conserved exported protein of unknown function
Mfumv2_1594	1215			81	24	14	5	81	73		conserved protein of unknown function
Mfumv2_1595	96			19	1	0	0	8	1		protein of unknown function
Mfumv2_1596	2427			75	178	91	138	34	215		Outer membrane receptor protein, mostly Fe transport (Modular protein)
Mfumv2_1597	615			150	66	0	0	10	6		conserved protein of unknown function
Mfumv2_1598	384			54	16	100	15	31	14		conserved protein of unknown function
Mfumv2_1599	1524			47	29	80	19	58	58		RecG-like helicase (fragment)
Mfumv2_1600	612	wrbA		401	207	288	93	184	290		putative conserved flavoprotein
Mfumv2_1601	786			67	52	6	1	27	47		conserved membrane protein of unknown function
Mfumv2_1602	2442	pps		44	128	10	9	20	117		phosphoenolpyruvate synthase
Mfumv2_1603	585			19	10	0	0	9	8		conserved protein of unknown function
Mfumv2_1604	1242	pmoB3		58	73	13	6	28	84		Particulate methane monooxygenase subunit alpha
Mfumv2_1605	750	pmoA3		143	118	17	5	57	104		Particulate methane monooxygenase subunit beta
Mfumv2_1606	864	pmoC3		539	525	209	48	181	509		Particulate methane monooxygenase subunit gamma
Mfumv2_1607	159			5	2	0	0	5	1		protein of unknown function
Mfumv2_1608	390	gloA		153	66	0	0	90	122		Lactoylglutathione lyase family protein
Mfumv2_1609	576			124	72	44	12	56	98		conserved protein of unknown function
Mfumv2_1610	1581	betA		127	217	44	28	67	292		Glucose-methanol-choline (GMC) oxidoreductase:NAD binding subunit
Mfumv2_1611	1392	glcD		38	63	23	10	54	207		Glycolate oxidase subunit GlcD
Mfumv2_1612	1332	glpC		79	118	37	16	87	309		Glycolate dehydrogenase, iron-sulfur subunit GlcF
Mfumv2_1613	1977	acoR		525	1059	259	208	388	2011		Transcriptional activator of acetoin/glycerol metabolism
Mfumv2_1614	1065	aroF	*	1282	1484	1699	639	1285	4307	*	3-deoxy-D-arabino-heptulosonate-7-phosphate synthase, tyrosine-repressible
Mfumv2_1615	1098	mutY		55	26	43	9	85	113		A/G-specific DNA glycosylase
Mfumv2_1616	570			385	38	152	8	176	65		Ribonuclease
Mfumv2_1617	141			41	6	64	7	16	5		conserved protein of unknown function
Mfumv2_1618	144			134	19	233	17	64	30		conserved protein of unknown function
Mfumv2_1619	744			56	47	31	12	69	137		putative Transcriptional regulator, Crp/Fnr family
Mfumv2_1620	483	cyoA	*	3877	1632	792	245	36	51	*	Heme/copper-type cytochrome oxidase, subunit 2
Mfumv2_1621	1686	cyoB	*	1214	2126	421	429	50	282	*	Heme/copper-type cytochrome oxidase, subunit 1
Mfumv2_1622	150			374	66	144	11	21	11		protein of unknown function
Mfumv2_1623	132			213	35	230	12	22	6		conserved protein of unknown function
Mfumv2_1624	216			70	15	54	4	16	8		conserved protein of unknown function
Mfumv2_1625	153			15	1	8	1	8	3		conserved protein of unknown function
Mfumv2_1626	186			6	1	0	0	4	1		conserved protein of unknown function
Mfumv2_1628	318			18	7	46	3	5	3		conserved membrane protein of unknown function
Mfumv2_1629	162			42	8	0	0	17	7		conserved protein of unknown function
Mfumv2_1630	906			65	18	39	8	78	52		A/G-specific DNA glycosylase (fragment)
Mfumv2_1631	786	rnhC		268	89	205	28	126	110		Ribonuclease HIII
Mfumv2_1632	2073	recG		102	97	94	51	81	206		RecG-like helicase
Mfumv2_1633	1593	degQ		288	497	225	179	226	924		Serine protease Do (Heat-shock protein)
Mfumv2_1634	855	glpG		314	267	191	84	235	646		Membrane associated serine protease, Rhomboid family
Mfumv2_1635	1104	glpX	*	911	1016	626	329	591	1815	*	Fructose-1,6-bisphosphatase class 2
Mfumv2_1637	618	nadD		139	74	48	14	75	122		putative nicotinate-nucleotide adenylyltransferase
Mfumv2_1638	438	rsfS		214	94	83	14	164	246		Ribosomal silencing factor RsfS

Mfumv2_1639	1023	kch		65	79	119	42	146	397		Kef-type K+ transport system, predicted NAD-binding component
Mfumv2_1640	939	wcaG		65	73	86	34	342	723		Nucleoside-diphosphate-sugar epimerase
Mfumv2_1641	1062	tsaD		249	279	197	68	468	1319		tRNA N6-adenosine threonylcarbamoyltransferase
Mfumv2_1642	1170	ndk		430	576	366	212	1064	3351		Nucleoside diphosphate kinase
Mfumv2_1643	1164	aspC	*	415	582	330	199	986	3172	*	Aspartate aminotransferase
Mfumv2_1644	408			174	91	36	9	395	380		Predicted membrane protein
Mfumv2_1645	1173			97	111	44	21	148	493		conserved exported protein of unknown function
Mfumv2_1646	924	mdh		454	436	527	203	269	693		Malate dehydrogenase
Mfumv2_1647	531			38	11	32	3	23	12		conserved protein of unknown function
Mfumv2_1648	198			0	0	19	1	15	3		conserved protein of unknown function
Mfumv2_1649	102			140	13	244	6	68	12		conserved protein of unknown function
Mfumv2_1650	228			90	17	172	6	57	49		conserved protein of unknown function
Mfumv2_1651	354			84	31	69	10	84	79		Addiction module toxin, RelE/StbE
Mfumv2_1652	1248			45	65	30	18	81	279		conserved protein of unknown function
Mfumv2_1653	642	spoIVFB		243	143	174	42	325	636		Zn-dependent protease
Mfumv2_1654	1035	mrp		297	317	178	100	445	1080		Protein mrp homolog
Mfumv2_1655	219			495	116	214	15	286	153		conserved protein of unknown function
Mfumv2_1656	1425			232	313	268	130	418	1370		conserved protein of unknown function
Mfumv2_1657	564			124	67	59	20	130	214		conserved membrane protein of unknown function
Mfumv2_1658	225			3030	561	1012	88	268	175		conserved protein of unknown function
Mfumv2_1659	792			1138	666	286	87	201	339		ABC-type multidrug transport system, permease component
Mfumv2_1660	726	ccmA		142	93	40	15	95	207		ABC-type multidrug transport system, ATPase component
Mfumv2_1661	438	dtd	*	18	10	6	2	40	64	*	D-tyrosyl-tRNA(Tyr) deacylase
Mfumv2_1662	1158	kefB		81	104	55	34	103	366		Na+/H+ antiporter
Mfumv2_1664	993	pyrD		247	282	285	100	193	517		Dihydroorotate dehydrogenase
Mfumv2_1666	162			90	13	55	7	41	21		conserved protein of unknown function
Mfumv2_1667	606			176	99	315	48	192	365		TPR repeats containing protein
Mfumv2_1668	2703	cirA		103	129	107	54	173	399		Outer membrane receptor protein, mostly Fe transport
Mfumv2_1669	1683			228	270	478	231	83	283		protein of unknown function
Mfumv2_1670	2703	cirA		47	59	62	21	53	142		Outer membrane receptor protein, mostly Fe transport
Mfumv2_1671	180			56	10	132	11	119	70		conserved protein of unknown function
Mfumv2_1672	1482			646	660	889	395	471	1073		transposase
Mfumv2_1673	285			22	9	116	12	29	15		conserved protein of unknown function
Mfumv2_1674	117			0	0	0	0	0	0		conserved protein of unknown function
Mfumv2_1675	3255	acrB		56	218	33	67	75	715		Cation/multidrug efflux pump
Mfumv2_1676	3192	acrB		93	306	67	79	83	770		Cation/multidrug efflux pump
Mfumv2_1677	507			347	201	597	95	430	705		conserved exported protein of unknown function
Mfumv2_1678	585	rpoE	*	1476	846	1819	414	834	1447	*	RNA polymerase sigma factor
Mfumv2_1679	1545	tolC		96	143	50	41	126	555		Outer membrane protein
Mfumv2_1680	162			0	0	0	0	21	12		conserved protein of unknown function
Mfumv2_1681	1083			413	510	548	225	307	756		HD-superfamily hydrolase
Mfumv2_1682	897	purC		269	242	256	99	250	656		Phosphoribosylaminoimidazole-succinocarboxamide synthase
Mfumv2_1683	2532	wcaA		101	281	131	103	115	736		Glycosyltransferase
Mfumv2_1684	1854	mdlB		136	291	159	112	141	823		ABC-type multidrug transport system, ATPase and permease component
Mfumv2_1685	1146			318	414	305	152	184	492		Xre family transcriptional regulator fused to periplasmic substrate-binding domain
Mfumv2_1686	1113	ftsZ	*	356	417	267	124	556	2115	*	Cell division GTPase FtsZ
Mfumv2_1687	1239	ftsA	*	232	332	304	170	302	1055	*	Cell division protein ftsA
Mfumv2_1688	870	ftsQ	*	158	184	282	90	196	462	*	Cell division septal protein FtsQ
Mfumv2_1690	930	ddl		325	364	395	125	504	1420		D-alanine--D-alanine ligase
Mfumv2_1691	2286	murC		314	845	454	354	382	2301		UDP-N-acetylmuramate-alanine ligase and UDP-N-acetylmuramate dehydrogenase
Mfumv2_1692	1110	murG		244	326	323	134	342	1036		UDP-N-acetylglucosamine--N-acetylmuramyl-(pentapeptide) pyrophosphoryl-undecaprenol N-acetylglucosamine transferase
Mfumv2_1693	1182	ftsW	*	75	111	49	33	163	619	*	Cell division protein FtsW
Mfumv2_1694	687			201	144	93	34	314	666		Predicted glycosyl hydrolase
Mfumv2_1695	1101	mraY		109	133	161	60	175	457		phospho-N-acetylmuramoyl-pentapeptide transferase
Mfumv2_1696	1380	murF		164	276	300	156	219	892		UDP-N-acetylmuramoyl-tripeptide--D-alanyl-D-alanine ligase
Mfumv2_1697	795			275	73	798	72	161	62		protein of unknown function

Mfumv2_1698	258			8	0	0	0	0	0		conserved protein of unknown function
Mfumv2_1699	357			437	54	1269	55	278	59		conserved protein of unknown function
Mfumv2_1701	240			695	162	1026	106	215	84		conserved protein of unknown function
Mfumv2_1702	1488	murE	*	159	278	200	119	336	1640	*	UDP-N-acetylmuramoyl-L-alanyl-D-glutamate--2,6-diaminopimelate ligase
Mfumv2_1703	177			5	2	0	0	12	7		protein of unknown function
Mfumv2_1704	1815	ftsI	*	195	413	326	215	197	912	*	Cell division protein FtsI
Mfumv2_1705	339			300	125	531	70	345	303		conserved protein of unknown function
Mfumv2_1706	945	rsmH		338	439	575	187	540	1536		Ribosomal RNA small subunit methyltransferase H
Mfumv2_1707	441	mraZ	*	819	442	1382	248	824	1087	*	Protein MraZ
Mfumv2_1709	690	gcd		195	145	183	65	188	363		Nucleoside-diphosphate-sugar pyrophosphorylase involved in lipopolysaccharide biosynthesis
Mfumv2_1710	1005			105	116	123	47	100	308		conserved membrane protein of unknown function
Mfumv2_1711	918			126	119	157	49	105	256		conserved protein of unknown function
Mfumv2_1712	981			217	221	477	134	232	549		conserved protein of unknown function
Mfumv2_1713	159			7	1	35	1	52	28		conserved protein of unknown function
Mfumv2_1714	1050	ctaA	*	596	646	250	185	186	573	*	Heme A synthase, cytochrome oxidase biogenesis protein CtaA
Mfumv2_1715	951	ctaB	*	534	543	235	143	155	418	*	Protoheme IX farnesyltransferase
Mfumv2_1716	1434	nrfG		111	181	117	69	182	746		TPR repeats containing protein
Mfumv2_1717	882			72	58	115	29	182	408		conserved protein of unknown function
Mfumv2_1718	543	pncA		737	427	1052	224	441	440		Amidase
Mfumv2_1719	1833	typA	*	763	1411	702	454	628	3100	*	GTP-binding protein TypA/BipA homolog
Mfumv2_1720	927			176	168	138	47	216	527		conserved protein of unknown function
Mfumv2_1721	1095			38	44	31	16	36	107		Uncharacterized conserved membrane protein
Mfumv2_1722	360			0	0	0	0	0	0		protein of unknown function
Mfumv2_1723	243			24	0	37	0	10	0		conserved protein of unknown function
Mfumv2_1724	270			0	0	0	0	4	1		protein of unknown function
Mfumv2_1725	291			35	10	28	3	7	4		conserved protein of unknown function
Mfumv2_1726	129			32	2	63	3	4	2		conserved protein of unknown function
Mfumv2_1727	1299		*	89	112	130	60	104	387	*	Nuclease of restriction endonuclease-like fold,RmuC family
Mfumv2_1728	2154	ppk	*	159	415	126	92	249	1507	*	Polyphosphate kinase
Mfumv2_1729	396	rplQ	*	801	405	1046	209	922	953	*	50S ribosomal subunit protein L17
Mfumv2_1730	1014	rpoA	*	953	1251	1913	781	2350	5987	*	RNA polymerase, alpha subunit
Mfumv2_1731	609	rpsD	*	977	833	1418	322	3984	8314	*	30S ribosomal subunit protein S4
Mfumv2_1732	543	rpsK	*	3262	2130	5189	974	3226	4307	*	30S ribosomal protein S11
Mfumv2_1733	396	rpsM	*	1866	870	2322	338	4458	5959	*	30S ribosomal subunit protein S13
Mfumv2_1734	783	map		1922	1591	1169	333	2170	4020		Methionine aminopeptidase
Mfumv2_1735	270	rpsT	*	299	84	200	17	1337	1069	*	30S ribosomal protein S20
Mfumv2_1736	1743	argS	*	83	151	67	47	161	780	*	Arginine--tRNA ligase
Mfumv2_1737	1092	moxY		292	339	428	144	454	1453		Methanol utilization control sensor protein
Mfumv2_1738	699	mxaB		163	138	291	57	288	496		DNA-binding response regulator, NarL family (REC-HTH domains)
Mfumv2_1739	1521			35	58	29	14	73	306		conserved membrane protein of unknown function
Mfumv2_1741	1248			234	322	323	153	194	531		conserved protein of unknown function
Mfumv2_1742	417			3286	315	2144	187	998	349		transposase
Mfumv2_1743	1062			182	156	923	240	16	26		protein of unknown function
Mfumv2_1744	1512			57	92	170	117	38	167		conserved membrane protein of unknown function
Mfumv2_1745	1395	bioA	*	89	159	130	55	138	467	*	Adenosylmethionine-8-amino-7-oxononanoate aminotransferase
Mfumv2_1746	1179			202	275	306	117	229	667		8-amino-7-oxononanoate synthase
Mfumv2_1747	1221	ssnA		255	355	213	91	234	724		Cytosine deaminase or related metal-dependent hydrolase
Mfumv2_1749	660	pgpB		36	35	41	12	52	82		Membrane-associated phospholipid phosphatase
Mfumv2_1750	2187	copA		276	646	192	209	212	1089		Cation transport ATPase
Mfumv2_1751	996	asd	*	466	514	674	250	650	1717	*	Aspartate-semialdehyde dehydrogenase
Mfumv2_1752	561			734	455	805	195	574	825		Integral membrane protein CcmA involved in cell shape determination
Mfumv2_1753	1773			127	252	90	78	155	773		conserved protein of unknown function
Mfumv2_1754	522			341	207	417	95	293	396		conserved exported protein of unknown function
Mfumv2_1755	699			373	293	230	67	395	883		conserved exported protein of unknown function
Mfumv2_1756	468			674	368	250	57	654	1048		Predicted metal transporter
Mfumv2_1757	399			2246	946	632	149	786	1151		conserved exported protein of unknown function

Mfumv2_1758	1656	cueO		1708	2730	928	741	423	1999		Multicopper oxidase family protein
Mfumv2_1759	696			2944	1982	1109	356	431	961		conserved protein of unknown function
Mfumv2_1760	135			482	50	204	19	132	57		conserved protein of unknown function
Mfumv2_1761	843			139	144	211	78	189	422		conserved protein of unknown function
Mfumv2_1762	1851	mdlB		152	366	218	170	261	1454		ABC-type multidrug transport system, ATPase and permease component
Mfumv2_1763	783	mhpC		58	51	76	18	88	176		Alpha/beta superfamily hydrolase
Mfumv2_1764	807			259	219	335	92	325	726		conserved protein of unknown function
Mfumv2_1765	1557			472	780	408	283	208	916		conserved exported protein of unknown function
Mfumv2_1766	165			1436	193	568	49	419	236		protein of unknown function
Mfumv2_1767	2565	cirA		58	119	21	27	33	236		Outer membrane receptor protein, mostly Fe transport
Mfumv2_1768	537			186	97	84	20	85	141		conserved protein of unknown function
Mfumv2_1769	1146	pntA		359	411	199	138	454	1366		fragment of pyridine nucleotide transhydrogenase, alpha subunit (part 1)
Mfumv2_1770	288	pntA		61	19	106	18	306	276		fragment of pyridine nucleotide transhydrogenase, alpha subunit (part 2)
Mfumv2_1771	1377	pntB		257	371	198	168	384	1463		pyridine nucleotide transhydrogenase, beta subunit
Mfumv2_1772	633			208	119	161	41	525	882		Globin domain
Mfumv2_1773	759			211	151	222	77	526	1352		conserved membrane protein of unknown function
Mfumv2_1774	1566			3067	4707	1718	1243	957	4011		conserved protein of unknown function
Mfumv2_1775	1563	ompA		91	146	67	43	171	812		Outer membrane protein or related peptidoglycan-associated (Lipo)protein
Mfumv2_1776	3039			365	1263	382	412	415	3500		conserved protein of unknown function
Mfumv2_1777	147			15	3	46	2	51	25		conserved protein of unknown function
Mfumv2_1779	765			150	112	114	37	215	491		conserved membrane protein of unknown function
Mfumv2_1780	738			108	74	183	42	149	344		conserved exported protein of unknown function
Mfumv2_1781	237			16	0	67	0	5	0		protein of unknown function
Mfumv2_1782	483			58	0	42	0	18	0		conserved protein of unknown function
Mfumv2_1784	249			170	39	342	23	57	27		conserved protein of unknown function
Mfumv2_1785	678	pgl	*	105	86	176	38	278	408	*	6-phosphogluconolactonase
Mfumv2_1786	1569	zwf	*	239	353	250	176	450	2173	*	Glucose-6-phosphate 1-dehydrogenase
Mfumv2_1787	693			1185	715	371	121	500	997		conserved exported protein of unknown function
Mfumv2_1788	831	pmoC4		43425	17912	355	144	5068	5600		Particulate methane monooxygenase subunit gamma
Mfumv2_1789	594			113	45	66	13	27	29		conserved protein of unknown function
Mfumv2_1790	141			38	4	0	0	5	3		conserved protein of unknown function
Mfumv2_1791	1290	pmoB2		2139	2387	1265	565	14899	46162		Particulate methane monooxygenase subunit alpha
Mfumv2_1792	738	pmoA2		2383	1422	1119	315	21207	32369		Particulate methane monooxygenase subunit beta
Mfumv2_1793	831	pmoC2		18136	7564	5467	1647	37218	50459		Particulate methane monooxygenase subunit gamma
Mfumv2_1794	1311	pmoB1		9550	15207	5003	2767	164	626		Particulate methane monooxygenase subunit alpha
Mfumv2_1795	801	pmoA1		9772	9322	3755	1545	41	87		Particulate methane monooxygenase subunit beta
Mfumv2_1796	828	pmoC1		47785	43583	34734	12994	207	329		Particulate methane monooxygenase subunit gamma
Mfumv2_1797	192			31	6	27	4	6	3		conserved protein of unknown function
Mfumv2_1798	246			693	190	736	81	163	127		conserved protein of unknown function
Mfumv2_1799	699	citB		7016	5395	4126	1176	1063	2718		DNA-binding response regulator, NarL family (REC-HTH domains)
Mfumv2_1800	1110			460	492	459	220	52	163		ABC-type multidrug transport system, permease component
Mfumv2_1801	1125	ybhS		73	90	60	21	41	126		putative transporter subunit: permease component of ABC superfamily
Mfumv2_1802	1752	ybhF		60	110	84	50	61	274		putative transporter fused subunits of ABC superfamily: ATP-binding components
Mfumv2_1803	879			165	141	156	43	65	166		conserved protein of unknown function
Mfumv2_1804	1350	tolC		70	97	62	41	45	166		Outer membrane protein
Mfumv2_1805	2079	nadE	*	102	218	66	60	138	718	*	Glutamine-dependent NAD(+) synthetase
Mfumv2_1806	774			115	109	262	67	180	319		4-Amino-4-deoxychorismate lyase
Mfumv2_1807	2184			193	446	120	131	157	928		Thiol:disulfide interchange protein
Mfumv2_1808	1455	uvrC		154	248	129	78	98	401		Excinuclease ABC subunit C
Mfumv2_1809	1047			50	33	122	31	35	30		protein of unknown function
Mfumv2_1810	246			124	38	172	13	94	48		conserved protein of unknown function
Mfumv2_1811	2283	purL		266	665	352	264	251	1587		Phosphoribosylformylglycinamidine synthase 2
Mfumv2_1812	1161	nagA		330	409	351	161	120	335		N-acetylglucosamine-6-phosphate deacetylase
Mfumv2_1813	1395	miaB		331	464	124	97	380	1662		(Dimethylallyl)adenosine tRNA methylthiotransferase MiaB
Mfumv2_1814	1206	yhhT		203	266	300	145	260	727		Predicted permease, member of the PurR regulon
Mfumv2_1815	3072		*	64	207	95	110	73	568	*	Proline dehydrogenase and delta 1-pyrroline-5-carboxylate dehydrogenase

Mfumv2_1816	1422	nrfG		75	110	145	72	127	479		TPR repeats containing protein
Mfumv2_1817	714	fabG		463	357	578	157	501	865		Short-chain alcohol dehydrogenase
Mfumv2_1818	594	ygfA		1302	798	1370	300	949	1599		5-formyltetrahydrofolate cyclo-ligase
Mfumv2_1819	156			718	95	323	21	274	108		conserved protein of unknown function
Mfumv2_1820	687			970	708	943	257	1358	3099		conserved exported protein of unknown function
Mfumv2_1821	555			794	428	614	147	554	938		conserved exported protein of unknown function
Mfumv2_1822	207			1529	271	1880	139	736	450		conserved protein of unknown function
Mfumv2_1824	168			83	9	69	4	22	7		conserved protein of unknown function
Mfumv2_1825	246			15	3	24	2	4	4		conserved protein of unknown function
Mfumv2_1826	5103		*	471	2850	1418	2756	516	4746	*	putative Transcription elongation factor GreA/GreB domain protein
Mfumv2_1827	180			10	1	0	0	4	1		conserved protein of unknown function
Mfumv2_1828	264			0	0	0	0	95	0		conserved protein of unknown function
Mfumv2_1829	327			14	1	16	2	0	0		conserved protein of unknown function
Mfumv2_1830	240			221	17	106	7	14	2		conserved protein of unknown function
Mfumv2_1831	1068			67	75	47	16	44	144		conserved protein of unknown function
Mfumv2_1832	588			481	333	228	77	190	361		Globin domain
Mfumv2_1833	507	ispF		165	85	126	29	155	189		2-C-methyl-D-erythritol 2,4-cyclodiphosphate synthase
Mfumv2_1834	870			155	140	92	35	328	1031		3-hydroxyisobutyrate dehydrogenase and cupin domain
Mfumv2_1835	288		*	294	80	345	44	432	412	*	3-hydroxyisobutyrate dehydrogenase and cupin domain
Mfumv2_1836	1725			100	197	191	92	103	446		Signal transduction histidine kinase with GAF domain
Mfumv2_1837	2715	glnD		124	400	136	138	156	1178		Bifunctional uridylyltransferase/uridylyl-removing enzyme
Mfumv2_1838	744	pdxJ		217	164	220	63	280	610		pyridoxine 5'-phosphate synthase
Mfumv2_1839	372	acpS		1355	550	2095	271	507	493		Holo-[acyl-carrier-protein] synthase
Mfumv2_1840	333			8043	2817	10698	1328	8236	10899		conserved protein of unknown function
Mfumv2_1841	930	araC		246	247	306	117	469	1145		Transcriptional regulator containing HTH domain,AraC family
Mfumv2_1842	756	kdpE		190	168	275	78	187	359		DNA-binding response regulator in two-component regulatory system with KdpD
Mfumv2_1843	2712	kdpD		240	681	213	244	208	1691		Osmosensitive K+ channel histidine kinase KdpD
Mfumv2_1844	663	rplY	*	1315	867	1235	380	1695	2895	*	50S ribosomal protein L25
Mfumv2_1845	555	pth		238	173	269	58	889	1450		Peptidyl-tRNA hydrolase
Mfumv2_1846	336	rpsF	*	744	286	1154	147	629	401	*	30S ribosomal protein S6
Mfumv2_1847	441	ssb		2423	1182	4876	743	2418	2542		Single-stranded DNA-binding protein
Mfumv2_1848	579	rplI	*	656	451	989	273	1373	2086	*	50S ribosomal protein L9
Mfumv2_1849	2703			76	229	61	86	189	1348		Outer membrane protein
Mfumv2_1850	600	hlpA		2295	1195	1286	451	1104	1876		Outer membrane protein
Mfumv2_1851	1062	lpxD		259	322	383	183	563	1616		UDP-3-O-acylglucosamine N-acyltransferase
Mfumv2_1852	1317	hom	*	331	478	266	178	475	1605	*	Homoserine dehydrogenase
Mfumv2_1853	1101	thrC	*	259	353	178	101	473	1207	*	Threonine synthase
Mfumv2_1854	1368	leuC		774	1169	824	442	1205	3687		Isopropylmalate/citramalate isomerase large subunit
Mfumv2_1855	342			471	184	1007	100	510	416		conserved exported protein of unknown function
Mfumv2_1856	2367	mgtA		54	134	50	40	58	355		Cation transport ATPase
Mfumv2_1857	363			204	73	171	20	282	266		conserved exported protein of unknown function
Mfumv2_1858	174			17	2	7	1	15	7		conserved protein of unknown function
Mfumv2_1859	222			24	6	0	0	7	4		transposase (fragment)
Mfumv2_1860	150			39	3	0	0	0	0		conserved protein of unknown function
Mfumv2_1861	132			6	2	0	0	4	2		conserved protein of unknown function
Mfumv2_1862	1602			31	39	27	14	16	44		conserved membrane protein of unknown function
Mfumv2_1863	663			195	74	185	44	105	138		conserved protein of unknown function
Mfumv2_1864	159			148	0	110	0	188	0		protein of unknown function
Mfumv2_1865	531			22	11	2	1	14	11		conserved protein of unknown function
Mfumv2_1866	660			34	27	35	9	10	19		conserved protein of unknown function
Mfumv2_1867	537			22	0	7	1	9	1		conserved protein of unknown function
Mfumv2_1868	657			2	2	6	3	16	25		conserved protein of unknown function
Mfumv2_1869	1098			16	17	14	4	13	26		conserved membrane protein of unknown function
Mfumv2_1870	657			59	30	32	13	24	46		conserved protein of unknown function
Mfumv2_1871	531			31	2	26	0	27	4		conserved protein of unknown function
Mfumv2_1872	654			65	21	12	4	16	17		conserved protein of unknown function

Mfumv2_1873	159			179	0	48	0	173	0		protein of unknown function
Mfumv2_1874	1095			52	46	26	11	12	22		conserved membrane protein of unknown function
Mfumv2_1875	657			43	23	54	10	50	86		conserved protein of unknown function
Mfumv2_1876	654			24	15	36	10	19	33		conserved protein of unknown function
Mfumv2_1877	1236			25	44	34	14	92	308		conserved protein of unknown function
Mfumv2_1878	657			48	25	32	10	50	108		conserved protein of unknown function
Mfumv2_1879	678			97	70	67	24	177	344		conserved protein of unknown function
Mfumv2_1881	120			95	12	0	0	39	13		conserved protein of unknown function
Mfumv2_1882	840	dacC		302	254	184	80	258	647		D-alanyl-D-alanine carboxypeptidase
Mfumv2_1883	510		*	47	29	40	5	42	48	*	Uri superfamily endonuclease (Modular protein)
Mfumv2_1884	132			6	2	0	0	41	12		conserved protein of unknown function
Mfumv2_1885	561			88	51	25	11	164	255		conserved exported protein of unknown function
Mfumv2_1886	1458			91	147	71	47	243	1032		Glycosyltransferase
Mfumv2_1887	684	dfp		153	98	165	38	148	252		Phosphopantothenoylcysteine synthetase/decarboxylase
Mfumv2_1888	861	lipA		389	326	205	91	242	564		Lipoyl synthase
Mfumv2_1889	138			40	0	42	0	7	0		conserved protein of unknown function
Mfumv2_1890	141			17	0	32	0	17	0		conserved protein of unknown function
Mfumv2_1891	153			19	0	97	0	25	0		conserved protein of unknown function
Mfumv2_1892	150			190	26	114	5	83	37		conserved protein of unknown function
Mfumv2_1893	162			236	33	239	16	193	126		conserved protein of unknown function
Mfumv2_1894	768	amiC	*	261	232	459	125	265	456	*	N-acetylmuramoyl-L-alanine amidase
Mfumv2_1895	1071	ampC	*	102	115	94	32	90	261	*	Beta-lactamase class C and other penicillin binding protein
Mfumv2_1896	1089		*	30	37	31	13	61	200	*	conserved protein of unknown function
Mfumv2_1897	144			27	5	0	0	54	11		protein of unknown function
Mfumv2_1898	138			18	0	0	0	0	0		conserved protein of unknown function
Mfumv2_1899	141			0	0	0	0	12	3		conserved protein of unknown function
Mfumv2_1900	153			19	0	8	0	13	0		conserved protein of unknown function
Mfumv2_1901	1047			14	7	22	3	15	10		protein of unknown function
Mfumv2_1902	417			396	64	406	36	142	63		transposase
Mfumv2_1903	159			122	12	214	16	50	7		exported protein of unknown function
Mfumv2_1904	1299	potE	*	92	132	64	32	54	219	*	Amino acid transporter
Mfumv2_1905	1326	bioA	*	118	162	106	57	128	495	*	Adenosylmethionine-8-amino-7-oxononanoate aminotransferase
Mfumv2_1906	675			661	467	765	197	254	403		Predicted phosphoribosyltransferase
Mfumv2_1907	1221	argG		666	987	654	319	645	2277		Argininosuccinate synthase
Mfumv2_1908	1950	dxs		1034	2468	1288	1000	949	4322		1-deoxyxylulose-5-phosphate synthase, thiamine-requiring, FAD-requiring
Mfumv2_1909	246	xseB		1062	279	1246	102	971	767		Exodeoxyribonuclease 7 small subunit
Mfumv2_1910	516			615	354	1212	170	696	1077		conserved protein of unknown function
Mfumv2_1911	183			19	3	37	2	7	5		conserved protein of unknown function
Mfumv2_1912	2646	valS	*	202	634	122	154	365	2525	*	Valine--tRNA ligase
Mfumv2_1913	1041	recA		1216	1181	969	426	598	1821		DNA strand exchange and recombination protein with protease and nuclease activity
Mfumv2_1914	1878	sigA	*	1502	3106	1483	1022	2338	12695	*	RNA polymerase sigma factor SigA
Mfumv2_1915	1776	dnaG	*	138	255	201	112	188	919	*	DNA primase
Mfumv2_1916	1122			112	138	77	48	83	229		conserved protein of unknown function
Mfumv2_1917	786	mazG		177	144	153	54	368	757		Nucleoside triphosphate pyrophosphohydrolase/pyrophosphatase MazG
Mfumv2_1918	180			119	29	79	6	217	131		conserved protein of unknown function
Mfumv2_1919	753			37	20	11	4	60	145		Predicted deacylase
Mfumv2_1920	723			119	76	112	34	129	251		1-acyl-sn-glycerol-3-phosphate acyltransferase (Modular protein)
Mfumv2_1921	663	aat		189	122	86	21	286	575		Leucyl/phenylalanyl-tRNA--protein transferase
Mfumv2_1922	1239	cinA		216	324	339	142	245	728		putative competence-damage inducible protein
Mfumv2_1923	3021	secA		776	2586	749	966	672	5420		Protein translocase subunit SecA
Mfumv2_1924	1191	degQ		361	428	326	152	250	708		Serine protease Do
Mfumv2_1925	1083	trpD	*	172	225	287	103	217	541	*	Anthranilate phosphoribosyltransferase
Mfumv2_1926	1212	carA		318	488	350	139	453	1479		Carbamoyl-phosphate synthase small chain
Mfumv2_1927	1320	pyrC	*	214	313	208	104	328	1108	*	Dihydroorotase
Mfumv2_1928	981	pyrB	*	82	87	137	39	272	788	*	Aspartate carbamoyltransferase
Mfumv2_1929	549	leuD	*	582	300	475	131	730	1101	*	3-isopropylmalate dehydratase small subunit

Mfumv2_1930	1113	leuB	*	433	551	434	254	793	2059	*	3-isopropylmalate dehydrogenase
Mfumv2_1931	1140	lrp		886	996	556	370	813	2907		Transcriptional regulator, Lrp/AsnC family
Mfumv2_1932	1848	sul		118	250	113	80	161	801		Sulfate permease, MFS superfamily
Mfumv2_1933	1158	tyrS	*	396	567	927	336	451	1192	*	Tyrosine--tRNA ligase
Mfumv2_1934	768			1655	1485	2497	744	1055	1691		RNA-binding protein, RRM domain (Modular protein)
Mfumv2_1935	387			199	90	268	40	88	103		conserved protein of unknown function
Mfumv2_1936	774	thiD		659	513	412	153	365	729		Hydroxymethylpyrimidine/phosphomethylpyrimidine kinase
Mfumv2_1937	1029	qor		253	281	287	115	315	904		NADPH:quinone reductase or related Zn-dependent oxidoreductase
Mfumv2_1938	534			116	71	164	40	108	158		conserved protein of unknown function
Mfumv2_1939	1074	nrfG		116	122	104	39	189	648		TPR repeats containing protein
Mfumv2_1940	1194			36	55	18	10	203	700		conserved protein of unknown function
Mfumv2_1941	1596			117	215	82	72	156	594		conserved protein of unknown function
Mfumv2_1942	984			78	86	99	42	71	185		conserved exported protein of unknown function
Mfumv2_1943	1206			52	58	48	22	50	187		conserved exported protein of unknown function
Mfumv2_1944	435			436	175	381	79	112	110		Arsenate reductase
Mfumv2_1945	369	arsR		389	156	259	42	821	1173		Transcriptional regulator containing HTH domain,ArsR
Mfumv2_1946	1071			44	46	47	19	22	72		conserved membrane protein of unknown function
Mfumv2_1947	681			116	77	76	23	41	66		conserved protein of unknown function
Mfumv2_1948	1020	fixC		143	117	80	37	38	75		Dehydrogenase (Flavoprotein)
Mfumv2_1949	1056	bcsA		40	38	10	3	36	93		Naringenin-chalcone synthase
Mfumv2_1950	2412			99	204	56	57	68	397		conserved exported protein of unknown function
Mfumv2_1951	492			18	7	0	0	37	68		putative Starvation-inducible outer membrane lipoprotein
Mfumv2_1952	102			0	0	0	0	7	1		conserved protein of unknown function
Mfumv2_1953	222			16	4	41	2	13	4		conserved protein of unknown function
Mfumv2_1954	144			3	1	0	0	5	1		conserved protein of unknown function
Mfumv2_1955	276			20	9	98	6	40	29		conserved protein of unknown function
Mfumv2_1956	231			114	29	85	7	85	42		conserved protein of unknown function
Mfumv2_1958	135			57	12	192	6	69	22		protein of unknown function
Mfumv2_1959	252			684	193	908	89	1329	1286		conserved protein of unknown function
Mfumv2_1960	150			0	0	0	0	5	1		conserved protein of unknown function
Mfumv2_1961	1407	tolC		65	86	103	75	97	470		Outer membrane protein
Mfumv2_1962	1326		*	57	65	42	18	36	127	*	putative Glycine/d-amino acid oxidases (Deaminating)
Mfumv2_1963	132			10	2	0	0	4	2		conserved protein of unknown function
Mfumv2_1964	180			54	10	56	2	45	25		conserved protein of unknown function
Mfumv2_1965	1047			54	55	43	13	144	521		conserved protein of unknown function
Mfumv2_1966	720			50	40	52	15	32	76		conserved protein of unknown function
Mfumv2_1967	516			164	83	171	33	64	91		conserved protein of unknown function
Mfumv2_1968	2970			131	464	175	172	129	992		conserved protein of unknown function
Mfumv2_1969	753			231	187	250	88	253	553		conserved protein of unknown function
Mfumv2_1970	1440			156	230	73	64	200	744		putative amidase AF_1954
Mfumv2_1971	441	cynS	*	233	100	199	31	195	208	*	Cyanate hydratase
Mfumv2_1972	984			111	112	119	39	98	280		conserved exported protein of unknown function
Mfumv2_1973	1365			379	507	520	256	136	383		putative Nitrite reductase (NO-forming)
Mfumv2_1974	1764			59	129	51	39	86	429		conserved protein of unknown function
Mfumv2_1975	828			62	70	65	21	73	152		TPR repeats containing protein
Mfumv2_1976	261			170	40	31	3	45	40		conserved exported protein of unknown function
Mfumv2_1977	525			37	17	20	5	22	30		conserved protein of unknown function
Mfumv2_1978	1425	gltD		192	305	328	151	198	641		NADPH-dependent glutamate synthase beta chain or related oxidoreductase
Mfumv2_1979	1341	pyrD		130	205	149	68	201	727		Dihydroorotate dehydrogenase fused to Fe-S-cluster domain
Mfumv2_1980	1413	hyuA		160	264	300	116	223	867		D-hydantoinase
Mfumv2_1981	1245	argE	*	142	205	291	106	151	435	*	Acetylornithine deacetylase/Succinyl-diaminopimelate desuccinylase or related deacylase
Mfumv2_1982	1047			34	24	91	26	27	33		protein of unknown function
Mfumv2_1983	135			1815	128	1988	62	1318	118		conserved protein of unknown function
Mfumv2_1984	642			73	50	59	18	94	158		conserved exported protein of unknown function
Mfumv2_1985	1884			68	117	30	30	61	338		putative Peptidase S9, prolyl oligopeptidase
Mfumv2_1986	975			229	219	149	69	74	193		putative enzyme of poly-gamma-glutamate biosynthesis (Capsule formation)

Mfumv2_1987	747			34	26	37	18	175	290		Predicted permease
Mfumv2_1988	876	prkB	*	533	565	501	189	946	2116	*	phosphoribulokinase
Mfumv2_1989	1071	fbp	*	706	781	955	392	1068	3280	*	Fructose-1,6-bisphosphatase class 1
Mfumv2_1990	1608	tolC		83	139	44	47	110	564		Outer membrane protein
Mfumv2_1991	3252	acrB		65	247	54	89	104	1006		Cation/multidrug efflux pump
Mfumv2_1992	183			1985	348	1370	75	174	99		conserved protein of unknown function
Mfumv2_1993	1524	proS	*	196	329	179	108	350	1456	*	Proline--tRNA ligase
Mfumv2_1994	288	gatC	*	275	82	233	24	576	553	*	Glutamyl-tRNA(Gln) amidotransferase subunit C 1
Mfumv2_1995	1452	gatA	*	291	483	258	181	651	2550	*	Glutamyl-tRNA(Gln) amidotransferase subunit A
Mfumv2_1996	1458	gatB	*	457	765	226	195	852	3522	*	Aspartyl/glutamyl-tRNA(Asn/Gln) amidotransferase subunit B
Mfumv2_1997	1797			704	1426	1660	953	383	1729		N-acetylglucosamine kinase fused to sugar phosphate isomerase
Mfumv2_1998	2076	ligA	*	297	613	185	181	600	4354	*	DNA ligase
Mfumv2_1999	678			233	195	125	31	206	383		LysM domain containing protein (Modular protein)
Mfumv2_2000	1509	glpK		113	170	73	47	80	397		Glycerol kinase
Mfumv2_2001	444			822	242	1853	254	554	208		protein of unknown function
Mfumv2_2002	1047			4	4	68	10	4	4		protein of unknown function
Mfumv2_2003	765	coaX		215	184	357	92	299	583		Type III pantothenate kinase
Mfumv2_2004	1548	htrA		1143	1864	1266	724	896	3626		putative periplasmic serine endoprotease DegP-like
Mfumv2_2006	468			3106	1361	2079	457	635	689		PIN domain containing protein
Mfumv2_2007	972	trpS	*	356	407	485	159	597	1552	*	Tryptophan--tRNA ligase
Mfumv2_2008	816		*	151	158	152	55	340	777	*	Chorismate mutase
Mfumv2_2009	1566			167	297	115	69	204	954		conserved protein of unknown function
Mfumv2_2010	741	aroD	*	90	71	57	22	154	356	*	3-dehydroquinate dehydratase
Mfumv2_2011	1137	mreB	*	1428	1719	1716	792	954	2682	*	cell wall structural complex MreBCD, actin-like component MreB
Mfumv2_2012	912	mreC	*	288	335	271	111	385	841	*	Cell shape-determining protein MreC
Mfumv2_2013	549			54	36	12	5	69	86		conserved membrane protein of unknown function
Mfumv2_2014	2028	ftsI	*	426	940	483	398	386	2297	*	Cell division protein FtsI
Mfumv2_2015	1182	rodA	*	110	131	55	40	243	1023	*	Rod shape-determining protein rodA
Mfumv2_2016	1572	cafA		204	409	245	154	225	971		Ribonuclease G and E
Mfumv2_2017	1266	serS	*	613	770	415	225	520	1843	*	Serine--tRNA ligase
Mfumv2_2018	2178	prc		681	1592	364	440	621	3740		Periplasmic protease
Mfumv2_2019	276	infA	*	2490	579	1507	184	1076	959	*	Translation initiation factor IF-1
Mfumv2_2020	876			197	166	162	66	249	685		Zn-dependent hydrolase of the beta-lactamase fold
Mfumv2_2021	1068			163	169	103	58	115	384		Zn-dependent hydrolase of the beta-lactamase fold (Modular protein)
Mfumv2_2022	1302	purD		170	249	205	106	183	588		Phosphoribosylamine--glycine ligase
Mfumv2_2023	906	htpX		1021	882	670	388	641	1568		membrane-associated Zn-dependent endopeptidase; self-cleaved
Mfumv2_2024	1446	gabD		241	356	161	99	344	1495		Succinate-semialdehyde dehydrogenase [NADP(+)]
Mfumv2_2025	819			311	197	94	40	153	436		conserved protein of unknown function
Mfumv2_2026	231			1775	388	5069	339	393	204		conserved protein of unknown function
Mfumv2_2027	426			195	89	112	23	200	300		Conserved membrane protein
Mfumv2_2028	678			250	211	157	61	348	731		Cu2+-binding oxygen sensor (SCO1/SenC/PrrC family)
Mfumv2_2030	795			209	177	170	78	188	418		ABC-type multidrug transport system, permease component
Mfumv2_2031	1020	ccmA		334	391	375	150	490	1479		ABC-type multidrug transport system, ATPase component
Mfumv2_2032	1413	kdtA		211	373	287	153	303	1177		3-deoxy-D-manno-octulosonic-acid transferase
Mfumv2_2033	972	xerD		183	210	226	91	256	878		Tyrosine recombinase XerC
Mfumv2_2034	216			86	14	78	8	57	28		conserved protein of unknown function
Mfumv2_2035	2784	ppc		95	273	55	64	207	1622		Phosphoenolpyruvate carboxylase
Mfumv2_2036	213			995	180	136	20	1024	793		conserved protein of unknown function
Mfumv2_2037	1515	leuA	*	784	1461	1096	573	1057	3573	*	2-isopropylmalate synthase
Mfumv2_2038	1029	ilvC	*	685	796	680	276	1264	3382	*	Ketol-acid reductoisomerase
Mfumv2_2039	474	ilvH	*	1016	434	421	89	821	1356	*	acetolactate synthase III, thiamin-dependent, small subunit
Mfumv2_2040	276	acyP		94	18	80	8	86	67		Acylphosphatase
Mfumv2_2041	177			0	0	0	0	4	1		conserved protein of unknown function
Mfumv2_2042	1125	bglX		188	228	177	97	195	593		Beta-glucosidase-related glycosidase
Mfumv2_2043	2169	caiC		165	436	163	159	334	1791		Acyl-CoA synthetase (AMP-forming)/AMP-acid ligase II
Mfumv2_2044	555	recO		104	59	142	23	118	211		Recombinational DNA repair protein RecO

Mfumv2_2045	333			70	29	149	16	140	141		putative metalloprotease Minf_1752
Mfumv2_2046	1620			311	561	201	121	277	1390		Predicted membrane-associated HD superfamily hydrolase
Mfumv2_2047	972	ybeZ		550	582	429	185	525	1326		putative enzyme with nucleoside triphosphate hydrolase domain
Mfumv2_2048	621			48	21	25	9	50	95		Predicted flavoprotein
Mfumv2_2049	1107	ald		107	113	106	55	171	631		Alanine dehydrogenase 2
Mfumv2_2050	675	lplA		52	47	84	16	126	212		Lipoate-protein ligase A
Mfumv2_2051	447	fkpA		356	168	265	60	440	633		Peptidyl-prolyl cis-trans isomerase
Mfumv2_2052	900			416	444	1318	365	582	1440		Farnesyl diphosphate synthase
Mfumv2_2053	336			1067	398	2793	271	1146	1161		conserved protein of unknown function
Mfumv2_2054	132			399	52	648	33	308	72		conserved protein of unknown function
Mfumv2_2055	417			637	187	270	55	151	75		transposase
Mfumv2_2056	1113			273	211	417	119	103	131		protein of unknown function
Mfumv2_2057	264			29	5	142	14	6	2		conserved protein of unknown function
Mfumv2_2058	1134	pstS	*	95	116	78	30	97	323	*	Phosphate-binding protein PstS
Mfumv2_2059	1026	pstC	*	69	76	88	30	57	180	*	Phosphate transport system permease protein PstC
Mfumv2_2060	840	pstA	*	41	40	41	18	74	180	*	phosphate transporter subunit ; membrane component of ABC superfamily
Mfumv2_2061	822	pstB	*	111	107	157	49	192	377	*	phosphate transporter subunit ; ATP-binding component of ABC superfamily
Mfumv2_2062	1155			98	124	105	40	100	342		Predicted Na+-dependent transporter
Mfumv2_2063	762	sdhB	*	298	284	313	71	229	421	*	Succinate dehydrogenase catalytic subunit
Mfumv2_2064	1956	sdhA	*	368	773	341	243	425	2240	*	Succinate dehydrogenase flavoprotein subunit
Mfumv2_2065	690	sdhC	*	209	125	88	38	173	333	*	Succinate dehydrogenase cytochrome b subunit
Mfumv2_2066	729			38	30	45	17	84	148		HAD superfamily hydrolase
Mfumv2_2067	1326	folC		356	448	126	74	188	716		Bifunctional folylpolyglutamate
Mfumv2_2068	1356	tlyC		110	159	66	56	191	692		Hemolysin or related protein containing CBS domains
Mfumv2_2070	669			58	42	50	17	138	220		conserved protein of unknown function
Mfumv2_2073	375			84	33	77	16	46	40		conserved protein of unknown function
Mfumv2_2074	396			83	37	54	9	149	98		conserved protein of unknown function
Mfumv2_2075	270			190	45	22	2	65	56		conserved protein of unknown function
Mfumv2_2076	420			84	40	152	18	119	157		conserved protein of unknown function
Mfumv2_2077	987	yhdN		113	102	75	30	187	487		General stress protein 69
Mfumv2_2078	1821	lepA		159	321	147	124	282	1494		GTP-binding membrane protein
Mfumv2_2079	1143	lepB		148	175	118	55	221	701		Signal peptidase I
Mfumv2_2080	171			563	84	52	7	46	18		conserved exported protein of unknown function
Mfumv2_2081	465			82	38	126	19	113	120		Universal stress protein
Mfumv2_2082	1689	fhs		396	703	457	285	282	1298		Formate--tetrahydrofolate ligase
Mfumv2_2083	1914	resB	*	298	642	241	220	385	2207	*	ResB protein required for cytochrome c biosynthesis
Mfumv2_2084	1050	pheS	*	164	230	185	98	324	842	*	Phenylalanine--tRNA ligase alpha subunit
Mfumv2_2085	2706	ileS	*	206	681	202	266	360	2404	*	Isoleucine--tRNA ligase
Mfumv2_2086	135			113	16	112	6	39	9		protein of unknown function
Mfumv2_2087	897			26136	25529	21632	8810	32425	102597		Opacity protein or related surface antigen
Mfumv2_2088	960	qor		338	351	300	116	432	1350		NADPH:quinone reductase
Mfumv2_2089	669	thiE		409	321	438	131	702	1534		Thiamine-phosphate synthase
Mfumv2_2090	1362	accC		963	1457	1350	596	684	2473		acetyl-CoA carboxylase, biotin carboxylase subunit
Mfumv2_2091	453			15	5	0	0	3	2		protein of unknown function
Mfumv2_2093	393			102	53	171	20	205	127		conserved protein of unknown function
Mfumv2_2094	612	tsf		1157	828	1454	295	1226	1942		Elongation factor Ts
Mfumv2_2095	747	rpsB		2305	1746	2512	646	4320	10294		30S ribosomal subunit protein S2
Mfumv2_2096	756			1848	1535	1531	498	2770	5254		conserved protein of unknown function
Mfumv2_2097	189			917	190	651	70	586	269		conserved protein of unknown function
Mfumv2_2098	549			224	126	229	47	111	123		conserved protein of unknown function
Mfumv2_2099	372	fur		348	159	334	51	637	778		Fe2+/Zn2+ uptake regulation protein, fur/PerR
Mfumv2_2100	126			28	4	0	0	12	2		protein of unknown function
Mfumv2_2101	246			43	14	180	12	8	4		conserved protein of unknown function
Mfumv2_2102	624			94	57	383	60	24	30		conserved protein of unknown function
Mfumv2_2103	1455	zraR		581	876	400	287	533	2283		Transcriptional regulatory protein ZraR
Mfumv2_2104	504	trxA		265	139	136	36	612	1069		Thiol-disulfide isomerase or thioredoxin

Mfumv2_2105	387			907	324	282	66	1052	1313		conserved exported protein of unknown function
Mfumv2_2106	561			391	222	181	65	581	964		conserved exported protein of unknown function
Mfumv2_2107	930	wcaA		49	53	87	32	146	421		Glycosyltransferase
Mfumv2_2109	954			89	95	87	36	93	239		putative Glycosyl transferase family 2
Mfumv2_2110	804	wcaA		189	158	285	67	296	497		Glycosyltransferase
Mfumv2_2111	786	cda		257	200	432	91	629	1144		Polysaccharide deacetylase family protein
Mfumv2_2112	942			58	49	65	17	55	155		Predicted glycosyltransferase
Mfumv2_2113	1140	rfaG		70	77	39	23	82	291		Glycosyltransferase
Mfumv2_2114	1047			175	209	100	55	199	638		conserved exported protein of unknown function
Mfumv2_2115	774	panB		235	166	147	51	149	275		3-methyl-2-oxobutanoate hydroxymethyltransferase
Mfumv2_2116	504	folK		86	42	48	13	91	127		2-amino-4-hydroxy-6-hydroxymethyldihyropteridine pyrophosphokinase
Mfumv2_2117	969	smtA		121	99	49	23	68	190		SAM-dependent methyltransferase
Mfumv2_2118	519			237	132	137	47	224	298		conserved exported protein of unknown function
Mfumv2_2119	780	cda		664	569	646	241	814	1981		Polysaccharide deacetylase family protein
Mfumv2_2120	1125	prpC	*	242	256	108	59	808	2510	*	2-methylcitrate synthase
Mfumv2_2121	240			346	80	238	37	282	149		conserved protein of unknown function
Mfumv2_2122	1947			52	104	54	43	118	498		conserved protein of unknown function
Mfumv2_2123	2538	nrfG		83	250	107	112	154	1218		TPR repeats containing protein
Mfumv2_2124	2844	uvrA		147	472	163	177	134	1069		UvrABC system protein A
Mfumv2_2125	774	yqxC		396	295	186	61	413	1136		putative rRNA methyltransferase YqxC
Mfumv2_2127	126			35	4	0	0	0	0		conserved protein of unknown function
Mfumv2_2128	201			4	1	34	2	9	5		conserved protein of unknown function
Mfumv2_2130	750			401	318	265	69	224	348		conserved protein of unknown function
Mfumv2_2131	1242			227	315	199	101	220	531		putative DNA modification methylase
Mfumv2_2132	1164			275	338	405	167	230	465		conserved protein of unknown function
Mfumv2_2133	2145			302	737	716	455	233	1060		conserved protein of unknown function
Mfumv2_2134	1413			159	271	248	117	170	484		conserved protein of unknown function
Mfumv2_2135	2244			213	529	363	278	116	574		ATPase-like
Mfumv2_2136	594			22	17	39	8	20	18		conserved protein of unknown function
Mfumv2_2137	225			4	2	11	2	2	2		conserved protein of unknown function
Mfumv2_2138	540	sixA		601	330	380	79	117	193		Phosphohistidine phosphatase SixA
Mfumv2_2139	1575	proP		34	54	24	13	84	336		Permease of the major facilitator superfamily
Mfumv2_2140	1275	eno	*	565	769	563	318	1129	4687	*	enolase
Mfumv2_2141	375		*	203	92	145	26	232	259	*	Septum formation initiator
Mfumv2_2142	1095	hemN		115	141	57	32	82	226		Coproporphyrinogen III oxidase or related Fe-S oxidoreductase
Mfumv2_2143	927			65	67	56	23	73	226		Predicted glycosyltransferase
Mfumv2_2144	747			52	46	51	16	93	220		conserved protein of unknown function
Mfumv2_2145	660			286	154	103	33	175	338		conserved protein of unknown function
Mfumv2_2146	441			147	57	57	12	62	78		SHS2 domain protein implicated in nucleic acid metabolism
Mfumv2_2147	120			170	15	0	0	14	5		conserved protein of unknown function
Mfumv2_2148	252			33	11	59	9	66	50		conserved protein of unknown function
Mfumv2_2149	180			96	15	95	5	4	3		conserved protein of unknown function
Mfumv2_2150	1431	rtcB		428	568	155	114	134	616		tRNA-splicing ligase RtcB
Mfumv2_2151	1104	spoIVFB		95	109	50	37	237	933		Zn-dependent protease fused to CBS domain
Mfumv2_2152	1194	ackA		56	74	34	18	175	700		Acetate kinase
Mfumv2_2153	1326			44	69	53	34	155	535		conserved exported protein of unknown function
Mfumv2_2154	1230	csd		587	749	414	240	193	696		putative cysteine desulfurase
Mfumv2_2155	423	iscU		121	65	58	14	74	76		NifU-like protein involved in Fe-S cluster formation
Mfumv2_2156	402			61	22	52	8	32	39		conserved exported protein of unknown function
Mfumv2_2157	414			112	43	15	5	213	297		conserved protein of unknown function
Mfumv2_2158	3168	mfd		120	464	104	122	266	2248		Transcription-repair-coupling factor
Mfumv2_2159	990	surA		156	176	156	58	237	753		Parvulin-like peptidyl-prolyl isomerase
Mfumv2_2160	885	pdxA		236	231	126	39	210	483		4-hydroxythreonine-4-phosphate dehydrogenase
Mfumv2_2161	1284	argB	*	110	165	46	36	176	780	*	Acetylglutamate kinase and N-acetylglutamate synthase family enzyme
Mfumv2_2162	585			221	126	231	70	111	175		conserved protein of unknown function
Mfumv2_2163	837			153	114	154	43	163	429		Sirohydrochlorin ferrochelatase

Mfumv2_2164	975	fba	*	1231	1236	605	345	1654	4895	*	Fructose-bisphosphate aldolase
Mfumv2_2165	300	rbpE		5388	1634	18309	1630	3628	3164		putative RNA-binding protein RbpE
Mfumv2_2166	789			749	610	1080	315	733	1169		conserved protein of unknown function
Mfumv2_2167	879			108	103	97	30	146	375		conserved protein of unknown function
Mfumv2_2168	369			80	30	36	7	110	108		Predicted permease
Mfumv2_2169	1245	dapL		538	620	500	310	574	1489		LL-diaminopimelate aminotransferase
Mfumv2_2170	2085	dinG	*	50	108	32	31	143	773	*	Rad3-related DNA helicase
Mfumv2_2171	357			2224	556	126	35	378	296		conserved exported protein of unknown function
Mfumv2_2172	342			122	25	24	4	48	22		conserved protein of unknown function
Mfumv2_2173	165			152	23	158	12	27	8		conserved protein of unknown function
Mfumv2_2174	132			261	34	433	22	63	24		conserved protein of unknown function
Mfumv2_2175	774	rluA		68	65	71	25	62	118		Pseudouridine synthase
Mfumv2_2176	1014	adhT		160	176	154	61	208	596		Alcohol dehydrogenase
Mfumv2_2177	702			7877	5062	13650	3562	3075	3178		conserved protein of unknown function
Mfumv2_2178	174			1845	322	3022	190	1616	767		conserved protein of unknown function
Mfumv2_2179	384			261	95	370	48	74	47		conserved protein of unknown function
Mfumv2_2180	1374	ysh		923	1351	955	570	578	1935		Predicted exonuclease of the beta-lactamase fold involved in RNA processing
Mfumv2_2181	1893	coxN		2588	5929	1486	1830	53	326		Alternative cytochrome c oxidase subunit 1
Mfumv2_2182	186			18	3	0	0	20	13		conserved protein of unknown function
Mfumv2_2183	300			50	6	0	0	146	32		conserved protein of unknown function
Mfumv2_2184	384		*	335	69	233	25	115	44	*	conserved exported protein of unknown function
Mfumv2_2185	1512	zwf	*	353	598	265	176	540	2545	*	Glucose-6-phosphate 1-dehydrogenase
Mfumv2_2186	1482	ppx		140	219	259	122	139	483		Exopolyphosphatase
Mfumv2_2187	117			121	16	72	4	362	172		protein of unknown function
Mfumv2_2188	156			237	42	294	14	80	18		conserved protein of unknown function
Mfumv2_2189	825	rsmA		113	93	122	35	294	729		Ribosomal RNA small subunit methyltransferase A
Mfumv2_2190	2364	metE	*	460	1283	607	598	1059	6287	*	5-methyltetrahydropteroyltriglutamate--homocysteine methyltransferase
Mfumv2_2191	495			423	233	461	91	351	388		Ca2+/H+ antiporter
Mfumv2_2192	663			57	48	56	12	105	160		Ca2+/H+ antiporter
Mfumv2_2194	2520	glgP	*	623	1699	631	611	594	4276	*	Glucan phosphorylase
Mfumv2_2195	1371			119	192	94	59	198	789		conserved exported protein of unknown function
Mfumv2_2196	795	smtA		415	332	171	68	374	847		SAM-dependent methyltransferase
Mfumv2_2197	762	rsmI		85	58	124	31	108	234		Ribosomal RNA small subunit methyltransferase I
Mfumv2_2198	444			282	128	220	58	215	309		conserved protein of unknown function
Mfumv2_2199	771	tolQ		165	120	115	43	171	371		MotA/TolQ/ExbB proton channel family protein
Mfumv2_2200	426	exbD		56	24	93	20	212	326		Biopolymer transport protein ExbD/TolR
Mfumv2_2201	798			45	46	32	14	151	333		conserved protein of unknown function
Mfumv2_2202	1176	guaB	*	468	641	435	184	1166	3466	*	IMP dehydrogenase/GMP reductase
Mfumv2_2203	1563	guaA	*	236	505	296	211	639	2350	*	GMP synthetase (glutamine aminotransferase)
Mfumv2_2204	1281	hisD	*	282	466	355	200	644	2468	*	Histidinol dehydrogenase
Mfumv2_2205	1128		*	3377	3661	2249	1096	1564	4594	*	putative ATP:guanido phosphotransferase Hore_00860
Mfumv2_2206	2511	clpB		4559	11438	3605	3623	2509	15708		Chaperone protein ClpB
Mfumv2_2207	477			108	58	192	24	127	152		conserved protein of unknown function
Mfumv2_2208	960	apbA		266	277	249	82	307	821		2-dehydropantoate 2-reductase
Mfumv2_2209	231			4100	804	2879	335	2028	1730		conserved protein of unknown function
Mfumv2_2211	351			589	239	449	42	518	575		conserved protein of unknown function
Mfumv2_2212	1413	acrA		404	633	628	302	388	1656		Membrane-fusion protein
Mfumv2_2213	3192	acrB		142	469	157	151	299	3243		Cation/multidrug efflux pump
Mfumv2_2214	897	yfjR	*	110	97	77	30	302	878	*	Uncharacterized oxidoreductase YfjR
Mfumv2_2215	327			902	266	328	61	147	143		conserved membrane protein of unknown function
Mfumv2_2216	1110	hisJ		132	128	62	29	225	553		Ligand-gated ion channel, periplasmic domain
Mfumv2_2217	777			3741	2522	3286	733	4490	8452		conserved protein of unknown function
Mfumv2_2218	957	rfaF		73	78	25	8	102	281		ADP-heptose:LPS heptosyltransferase
Mfumv2_2219	738	apaH		112	79	123	36	100	193		Metallophosphoesterase, PP2A family
Mfumv2_2220	969	cysK	*	1019	1040	899	332	1327	3822	*	cysteine synthase A, O-acetylserine sulfhydrolase A subunit
Mfumv2_2221	861	rpoD	*	1538	1263	1686	628	1495	3650	*	RNA polymerase sigma factor

Mfumv2_2222	540	apt		268	172	365	71	575	972		Adenine phosphoribosyltransferase
Mfumv2_2223	456			56	22	30	4	17	13		conserved protein of unknown function
Mfumv2_2224	402			40	17	84	8	10	9		conserved protein of unknown function
Mfumv2_2225	168			48	10	116	6	10	3		conserved protein of unknown function
Mfumv2_2226	129			91	16	194	7	38	18		conserved protein of unknown function
Mfumv2_2227	798	lpxA		186	173	148	41	155	330		Acyl-[acyl-carrier-protein]--UDP-N-acetylglucosamine O-acyltransferase
Mfumv2_2228	1326	hflX		660	808	420	213	289	1054		putative GTPase
Mfumv2_2229	936	miaA	*	32	33	19	8	84	257	*	tRNA dimethylallyltransferase
Mfumv2_2230	741			45	35	21	9	19	39		conserved protein of unknown function
Mfumv2_2231	1338			784	967	445	336	252	869		conserved membrane protein of unknown function
Mfumv2_2232	837			289	229	296	90	349	884		conserved protein of unknown function
Mfumv2_2233	213			25	8	6	1	184	112		conserved protein of unknown function
Mfumv2_2234	1818	cysJ		211	413	167	128	1229	6527		Sulfite reductase [NADPH] flavoprotein alpha-component
Mfumv2_2235	1128	nuoH	*	217	283	247	152	656	2248	*	NADH-quinone oxidoreductase subunit H 2
Mfumv2_2236	1707	nuoG	*	573	1304	939	689	1192	4989	*	NADH-ubiquinone oxidoreductase chain G
Mfumv2_2237	1371	nuoF	*	537	1072	510	293	1616	6609	*	NADH:ubiquinone oxidoreductase, chain F
Mfumv2_2238	534	nuoE	*	486	367	338	88	1392	2160	*	NADH-ubiquinone oxidoreductase chain E
Mfumv2_2239	1257	nuoD	*	468	755	448	229	1524	5478	*	NADH-quinone oxidoreductase subunit D
Mfumv2_2240	639	nuoC	*	895	653	767	185	1272	2216	*	NADH-quinone oxidoreductase subunit C
Mfumv2_2241	513	nuoB	*	1293	635	959	187	1810	2930	*	NADH-quinone oxidoreductase subunit B
Mfumv2_2242	495	wzb		44	20	14	3	92	142		Protein-tyrosine-phosphatase
Mfumv2_2243	1125	hisC	*	134	161	104	65	280	937	*	Histidinol-phosphate aminotransferase
Mfumv2_2244	858	tyrA	*	130	135	100	32	210	434	*	Prephenate dehydrogenase
Mfumv2_2245	1251	lolE		49	73	34	12	155	600		ABC-type transport system, involved in lipoprotein release, permease component
Mfumv2_2246	696	lolD		214	174	196	55	228	429		outer membrane-specific lipoprotein transporter subunit ; ATP-binding component of ABC superfamily
Mfumv2_2247	885	ilvE	*	172	199	163	64	355	884	*	putative branched-chain-amino-acid aminotransferase
Mfumv2_2248	141			86	11	18	2	53	14		conserved protein of unknown function
Mfumv2_2249	486	mcsA		2208	1075	1081	288	793	1191		Modulator of heat shock repressor CtsR, McsA
Mfumv2_2250	354			537	193	299	66	312	213		conserved protein of unknown function
Mfumv2_2251	159			162	22	171	12	121	41		conserved protein of unknown function
Mfumv2_2252	174			268	44	224	22	75	34		conserved exported protein of unknown function
Mfumv2_2253	1416	gndA	*	122	148	53	38	396	1942	*	6-phosphogluconate dehydrogenase, NADP(+)-dependent, decarboxylating
Mfumv2_2254	444	ywlF	*	810	359	630	138	566	793	*	putative sugar phosphate isomerase YwlF
Mfumv2_2255	621			107	57	81	17	68	101		conserved protein of unknown function
Mfumv2_2256	1191			199	234	118	62	100	303		putative Abortive infection protein
Mfumv2_2257	2802			115	351	87	106	175	1152		conserved exported protein of unknown function
Mfumv2_2258	342			2729	980	1697	290	7951	10608		conserved exported protein of unknown function
Mfumv2_2259	864	mmt		54	52	77	28	106	280		Predicted Co/Zn/Cd cation transporter
Mfumv2_2260	138			0	0	9	1	7	2		conserved protein of unknown function
Mfumv2_2262	723			52	44	104	22	40	52		putative ABC transporter
Mfumv2_2263	441			71	29	23	2	21	22		conserved protein of unknown function
Mfumv2_2264	279			6	1	29	3	3	1		conserved protein of unknown function
Mfumv2_2265	177			12	2	0	0	7	3		conserved protein of unknown function
Mfumv2_2266	195			0	0	0	0	2	2		conserved protein of unknown function
Mfumv2_2267	810			1155	937	1439	466	642	1724		conserved membrane protein of unknown function
Mfumv2_2268	987			750	705	665	290	201	508		conserved exported protein of unknown function
Mfumv2_2269	165			80	13	128	4	76	29		conserved protein of unknown function
Mfumv2_2270	195			130	28	153	9	464	296		conserved protein of unknown function
Mfumv2_2271	810			615	616	340	137	520	1013		Thioredoxin domain-containing protein
Mfumv2_2272	663			243	202	159	38	339	525		Predicted O-methyltransferase
Mfumv2_2273	873	proC	*	226	207	142	50	464	1286	*	Pyrroline-5-carboxylate reductase
Mfumv2_2274	528	aroK	*	260	138	87	21	606	933	*	Shikimate kinase
Mfumv2_2275	1089	aroC	*	355	416	228	101	374	1048	*	Chorismate synthase
Mfumv2_2276	639	plsY		68	56	73	18	158	330		Glycerol-3-phosphate acyltransferase
Mfumv2_2277	888	gpsA		155	153	101	48	250	616		Glycerol-3-phosphate dehydrogenase [NAD(P)+]
Mfumv2_2278	1851	glmS		966	2023	1096	805	754	3667		Glutamine--fructose-6-phosphate aminotransferase [isomerizing]

Mfumv2_2279	1308	glgC	*	120	179	89	66	276	1182	*	Glucose-1-phosphate adenylyltransferase
Mfumv2_2280	945			113	124	54	23	243	678		Predicted dioxygenase
Mfumv2_2281	1347	gltX	*	609	801	401	258	539	1824	*	Glutamate--tRNA ligase
Mfumv2_2282	612	pgsA		217	133	105	42	221	348		Phosphatidylglycerophosphate synthase
Mfumv2_2283	1377	der		108	181	79	41	241	945		GTPase Der
Mfumv2_2285	2154	bglX		79	184	55	49	107	667		Periplasmic beta-glucosidase
Mfumv2_2286	942	atoC		105	95	82	29	158	405		AAA-type ATPase and DNA-binding domains, NtrC family
Mfumv2_2287	156			26	4	25	3	121	45		protein of unknown function
Mfumv2_2288	1947	acsA		284	607	178	161	394	2232		Acetyl-coenzyme A synthetase
Mfumv2_2289	327			137	40	223	26	187	142		conserved membrane protein of unknown function
Mfumv2_2290	168			121	20	258	18	28	7		conserved protein of unknown function
Mfumv2_2291	186			8	2	7	1	4	1		conserved protein of unknown function
Mfumv2_2292	738			375	322	537	128	512	1102		conserved exported protein of unknown function
Mfumv2_2293	447			63	41	35	9	145	154		conserved protein of unknown function
Mfumv2_2294	960	fmt	*	330	325	367	144	443	1152	*	10-formyltetrahydrofolate:L-methionyl-tRNA(fMet) N-formyltransferase
Mfumv2_2295	150			0	0	0	0	21	11		exported protein of unknown function
Mfumv2_2296	981	sua	*	105	107	91	39	78	177	*	Threonylcarbamoyl-AMP synthase
Mfumv2_2297	132			481	59	1001	46	136	47		protein of unknown function
Mfumv2_2298	2616	gyrA	*	490	1634	557	557	629	4939	*	DNA gyrase subunit A
Mfumv2_2299	2523	gyrB	*	1090	2921	1327	1204	750	5179	*	DNA gyrase subunit B
Mfumv2_2300	705	phoB		249	164	128	43	185	403		DNA-binding response regulator in two-component regulatory system with PhoR (or CreC)
Mfumv2_2301	1305			38	56	35	21	82	334		Phosphate regulon sensor protein phoR
Mfumv2_2302	816			133	118	206	52	136	336		conserved protein of unknown function
Mfumv2_2303	807			123	92	80	27	131	308		conserved exported protein of unknown function
Mfumv2_2304	2184			439	1158	472	424	460	2707		conserved protein of unknown function
Mfumv2_2305	141			245	34	18	2	516	292		protein of unknown function
Mfumv2_2306	876			63	49	53	16	54	107		conserved protein of unknown function
Mfumv2_2307	1635	groEL		10986	17138	12256	6629	13340	49004		chaperone Hsp60, peptide-dependent ATPase, heat shock protein
Mfumv2_2308	300	groS		11826	3290	6910	794	5241	4132		Cpn10 chaperonin GroES, small subunit of GroESL
Mfumv2_2309	1914	dnaK		10725	16666	5573	3937	3887	18900		chaperone Hsp70, co-chaperone with DnaJ
Mfumv2_2310	1431	mpl		414	684	616	279	571	1957		UDP-N-acetylmuramate:L-alanyl-gamma-D-glutamyl-meso-diaminopimelate ligase
Mfumv2_2311	717			521	351	267	74	557	1277		conserved protein of unknown function
Mfumv2_2312	681	tadA		87	54	82	30	121	301		tRNA-specific adenosine deaminase
Mfumv2_2313	879			233	218	151	59	482	1263		Predicted aminomethyltransferase
Mfumv2_2314	138			341	42	121	8	524	246		conserved protein of unknown function
Mfumv2_2315	453			1149	498	801	127	3326	5618		conserved protein of unknown function
Mfumv2_2316	324	nrfG		67	17	96	15	113	81		TPR repeats containing protein
Mfumv2_2317	1539	cysG		174	363	262	157	308	1111		Uroporphyrinogen-III methylase and Uroporphyrinogen-III synthase
Mfumv2_2318	900	hemC		490	531	736	217	518	1191		Porphobilinogen deaminase
Mfumv2_2319	1065	hemA	*	164	210	182	68	420	1353	*	Glutamyl-tRNA reductase
Mfumv2_2320	810	ccmC	*	295	240	147	71	229	500	*	ABC-type transport system involved in cytochrome c biogenesis, permease component
Mfumv2_2321	447			2290	862	1085	275	906	1411		conserved protein of unknown function
Mfumv2_2322	144			482	63	311	10	671	373		conserved protein of unknown function
Mfumv2_2323	1119			247	269	222	113	364	1225		Predicted permease
Mfumv2_2324	174			662	110	290	26	429	199		protein of unknown function
Mfumv2_2325	1104	pepP		167	217	214	75	388	1114		Xaa-Pro aminopeptidase
Mfumv2_2326	1659	tolC		109	177	129	92	192	1029		Outer membrane protein
Mfumv2_2327	5505	uvrA		104	628	73	168	105	1645		Excinuclease ABC subunit A, ATPase
Mfumv2_2328	1620		*	181	339	156	133	250	1110	*	Pyruvate decarboxylase or related thiamine pyrophosphate-requiring enzyme
Mfumv2_2329	681	cusR		171	125	54	16	194	295		DNA-binding response regulator in two-component regulatory system with CusS
Mfumv2_2330	972	baeS		65	71	18	11	110	269		Signal transduction histidine kinase
Mfumv2_2331	702	tonB		747	487	263	105	477	963		Periplasmic protein TonB
Mfumv2_2332	2763	cirA		74	172	69	70	121	841		Outer membrane receptor protein, mostly Fe transport
Mfumv2_2333	2694	cirA		90	231	196	156	83	478		Outer membrane receptor protein, mostly Fe transport
Mfumv2_2334	2253	erg		132	334	102	99	201	1233		Phytoene/squalene synthetase fused to flavin containing amine oxidoreductase
Mfumv2_2335	978	dys		274	256	165	70	509	1597		Deoxyhypusine synthase

Mfumv2_2336	2109	hppA		324	765	324	313	572	3252		K(+)-insensitive pyrophosphate-energized proton pump
Mfumv2_2337	1425	nuoN	*	186	322	157	128	543	2160	*	NADH-quinone oxidoreductase subunit N
Mfumv2_2339	1467	nuoM	*	224	384	230	235	559	2397	*	NADH dehydrogenase subunit M
Mfumv2_2340	147			267	48	223	18	164	75		Pseudogene in nuo operon
Mfumv2_2341	1806	nuoL	*	355	773	302	339	646	3500	*	NADH-quinone oxidoreductase subunit L
Mfumv2_2342	306	nuoK	*	144	57	123	16	430	377	*	NADH-quinone oxidoreductase subunit K
Mfumv2_2343	498	nuoJ	*	352	212	185	51	831	1202	*	NADH dehydrogenase subunit J
Mfumv2_2344	519	nuoI	*	528	318	405	77	911	1364	*	NADH-quinone oxidoreductase subunit I
Mfumv2_2345	228			117	29	39	7	24	16		conserved protein of unknown function
Mfumv2_2347	2520			22	51	25	22	24	172		Outer membrane receptor protein, mostly Fe transport
Mfumv2_2348	1029	tas		2213	2458	1747	844	1194	2977		Predicted oxidoreductase
Mfumv2_2349	348			416	167	209	40	329	305		Predicted membrane protein
Mfumv2_2350	582	thiJ	*	652	420	368	85	637	1085	*	putative intracellular protease/amidase
Mfumv2_2351	606			448	296	204	69	796	1557		conserved protein of unknown function
Mfumv2_2352	687	pgsA		464	291	166	69	290	600		Phosphatidylglycerophosphate synthase
Mfumv2_2353	339			898	266	183	40	910	1164		conserved exported protein of unknown function
Mfumv2_2354	615		*	92	52	45	15	116	213	*	Cytochrome c553
Mfumv2_2355	585			49	27	66	16	85	152		Intracellular proteinase inhibitor
Mfumv2_2356	978			254	312	190	67	179	469		GDP-mannose 3,5-epimerase
Mfumv2_2357	1392	rfaG		118	188	119	61	159	639		Glycosyltransferase
Mfumv2_2358	2424	cirA		39	93	37	26	32	206		Outer membrane receptor protein, mostly Fe transport
Mfumv2_2359	1026			163	177	149	46	168	395		Rsm22 family methyltransferase
Mfumv2_2360	126			68	10	20	2	119	39		conserved protein of unknown function
Mfumv2_2361	2619	cirA		65	164	53	64	785	8243		Outer membrane receptor protein, mostly Fe transport
Mfumv2_2362	1209			44	49	17	8	94	428		conserved protein of unknown function
Mfumv2_2363	2223	cirA		75	157	96	72	153	1264		Outer membrane receptor protein, mostly Fe transport
Mfumv2_2364	1317	murD		80	119	76	45	197	727		UDP-N-acetylmuramoylalanine--D-glutamate ligase
Mfumv2_2365	1434			91	158	76	52	198	860		conserved protein of unknown function
Mfumv2_2366	744	gcd		157	146	133	48	380	890		Nucleoside-diphosphate-sugar pyrophosphorylase
Mfumv2_2367	1764	ptsA		254	521	112	105	436	1962		Phosphoenolpyruvate-protein phosphotransferase
Mfumv2_2368	318	phbH		773	260	301	63	1293	1423		Phosphocarrier protein HPr
Mfumv2_2369	981	hprK		474	486	137	64	943	2750		HPr kinase/phosphorylase
Mfumv2_2370	1110	trpE	*	87	116	129	48	159	381	*	Anthranilate/para-aminobenzoate synthase component I
Mfumv2_2371	1161	dnaJ		1369	1940	1357	584	1504	4623		Chaperone protein DnaJ
Mfumv2_2372	639	grpE		917	645	903	251	1370	3002		Protein GrpE
Mfumv2_2373	1242	rsbU		455	671	722	312	824	3232		Serine phosphatase RsbU, regulator of sigma subunit
Mfumv2_2374	528	rsbS		891	534	701	140	1435	2164		Anti-anti-sigma regulatory factor RsbS (Antagonist of anti-sigma factor)
Mfumv2_2375	189			32	5	24	1	51	32		conserved protein of unknown function
Mfumv2_2376	795	phnP		108	100	105	22	272	540		Metal-dependent hydrolase of the beta-lactamase superfamily
Mfumv2_2377	807	tatD		89	76	51	18	245	551		Mg-dependent DNase
Mfumv2_2378	1767	ilvD	*	632	1329	1349	794	697	2938	*	Dihydroxy-acid dehydratase
Mfumv2_2379	1338	trmFO	*	361	417	325	164	164	567	*	Methylenetetrahydrofolate--tRNA-(uracil-5-)-methyltransferase TrmFO
Mfumv2_2380	2328	pheT	*	492	1210	512	413	566	3703	*	Phenylalanine--tRNA ligase beta subunit
Mfumv2_2381	771			6925	4854	7303	1836	994	2134		Opacity protein or related surface antigen
Mfumv2_2382	645	trmB	*	557	349	351	104	240	378	*	tRNA (guanine-N(7)-)-methyltransferase
Mfumv2_2383	381	xylA		467	154	261	56	247	294		Dimeric dUTPase
Mfumv2_2384	447			82	25	28	4	63	75		Predicted thiol-disulfide oxidoreductase
Mfumv2_2385	240			14	3	5	1	9	5		protein of unknown function
Mfumv2_2386	753	wcaA		261	243	372	87	290	578		Glycosyltransferase
Mfumv2_2387	789	cda		78	59	82	26	135	238		Predicted xylanase/chitin deacetylase
Mfumv2_2388	1170			156	215	137	51	246	719		conserved membrane protein of unknown function
Mfumv2_2389	774	hisF	*	300	278	274	73	403	761	*	Imidazole glycerol phosphate synthase subunit HisF
Mfumv2_2390	522	trmL	*	143	71	146	23	191	284	*	tRNA (cytidine(34)-2'-O)-methyltransferase
Mfumv2_2391	462			331	141	616	69	330	407		conserved membrane protein of unknown function
Mfumv2_2392	774	truA	*	90	81	123	30	229	395	*	tRNA pseudouridine synthase A
Mfumv2_2393	684	ompW		429	365	472	117	891	1940		Outer membrane protein W

Mfumv2_2394	441			2234	1204	1678	302	1914	2170		putative Holliday junction resolvase
Mfumv2_2395	822	trpA	*	202	182	130	41	202	486	*	Tryptophan synthase alpha chain
Mfumv2_2396	186			10	1	0	0	55	38		conserved protein of unknown function
Mfumv2_2397	4560	gltB		906	4851	696	1479	1300	16372		Glutamate synthase [NADPH] large chain
Mfumv2_2398	357			1626	535	498	97	1376	1556		LysM domain containing protein
Mfumv2_2399	924	smtA		39	42	18	8	79	228		SAM-dependent methyltransferase
Mfumv2_2400	903	folP		126	129	95	38	208	457		Dihydropteroate synthase or related enzyme
Mfumv2_2401	612	hisB	*	229	170	253	65	396	606	*	Imidazoleglycerol-phosphate dehydratase
Mfumv2_2402	123			176	22	113	5	301	133		conserved protein of unknown function
Mfumv2_2403	624			223	125	82	23	176	287		conserved protein of unknown function
Mfumv2_2404	504			237	104	197	44	248	401		conserved exported protein of unknown function
Mfumv2_2405	1158			93	124	116	47	198	622		conserved membrane protein of unknown function
Mfumv2_2406	156			5	2	0	0	25	10		conserved protein of unknown function
Mfumv2_2407	732			35	32	34	11	64	122		conserved membrane protein of unknown function
Mfumv2_2408	1497	dhaS		130	236	317	138	108	338		putative aldehyde dehydrogenase DhaS
Mfumv2_2409	846			19	25	21	4	34	73		conserved protein of unknown function
Mfumv2_2410	1086	vioA	*	18	23	26	8	23	49	*	dTDP-4-amino-4,6-dideoxy-D-glucose transaminase
Mfumv2_2411	174			109	20	102	8	56	36		conserved protein of unknown function
Mfumv2_2412	234			38	12	25	2	29	20		conserved protein of unknown function
Mfumv2_2413	864	ubiB		114	124	254	65	228	525		2-polyprenylphenol hydroxylase or related flavodoxin oxidoreductase
Mfumv2_2414	1146	napF	*	237	367	259	99	357	1178	*	Ferredoxin
Mfumv2_2415	1026	pyrD		242	286	211	77	877	3430		Dihydroorotate dehydrogenase
Mfumv2_2416	3549	ydbK		224	866	153	238	125	1322		putative 2-oxoacid-flavodoxin fused oxidoreductase:conserved protein; 4Fe-4S cluster binding protein
Mfumv2_2417	1149	napF	*	273	347	252	111	181	584	*	Ferredoxin
Mfumv2_2418	462	crp		84	46	85	15	86	109		Transcriptional regulator, Crp family
Mfumv2_2419	846	ubiB		149	136	205	64	92	217		2-polyprenylphenol hydroxylase or related flavodoxin oxidoreductase
Mfumv2_2420	762	frhG		110	90	85	23	133	302		Coenzyme F420-reducing hydrogenase, gamma subunit
Mfumv2_2421	1314	frhA		86	133	52	32	109	397		Coenzyme F420-reducing hydrogenase, alpha subunit
Mfumv2_2422	1818		*	138	261	153	88	262	1024	*	putative Pyruvate kinase
Mfumv2_2423	129			166	24	65	4	116	39		conserved protein of unknown function
Mfumv2_2424	165			33	6	27	1	130	85		conserved protein of unknown function
Mfumv2_2425	2721			76	213	41	50	73	489		conserved protein of unknown function
Mfumv2_2426	126			321	48	457	34	93	30		protein of unknown function
Mfumv2_2427	156			179	32	392	21	67	29		conserved protein of unknown function
Mfumv2_2428	351			92	39	162	25	110	77		conserved protein of unknown function
Mfumv2_2429	891	rffH		204	227	170	56	365	984		glucose-1-phosphate thymidylyltransferase
Mfumv2_2430	1089	rffG		233	305	306	109	450	1344		dTDP-glucose 4,6-dehydratase
Mfumv2_2431	1047			56	34	222	49	31	33		protein of unknown function
Mfumv2_2432	228			656	53	1053	43	310	85		conserved protein of unknown function
Mfumv2_2433	603	ribE		161	113	280	55	391	674		Riboflavin synthase
Mfumv2_2434	729	fabG		275	228	472	110	392	754		Short-chain alcohol dehydrogenase
Mfumv2_2435	984			91	86	29	14	117	293		conserved protein of unknown function
Mfumv2_2436	1389			16	19	8	6	28	89		conserved membrane protein of unknown function
Mfumv2_2437	654			19	13	61	12	35	54		conserved protein of unknown function
Mfumv2_2438	762	pspE		120	115	121	27	221	497		Rhodanese-related sulfurtransferase
Mfumv2_2439	1899	gidA	*	382	743	113	87	508	2449	*	glucose-inhibited cell-division protein
Mfumv2_2440	1449	dnaB	*	142	235	89	60	286	1051	*	Replicative DNA helicase
Mfumv2_2441	795	thiG		447	352	391	121	374	710		thiamin biosynthesis ThiGH complex subunit
Mfumv2_2442	849			439	436	574	176	1042	3345		Predicted periplasmic solute-binding protein
Mfumv2_2443	945			459	456	158	65	768	1923		conserved protein of unknown function
Mfumv2_2444	225			358	76	343	28	162	68		conserved protein of unknown function
Mfumv2_2445	615			144	63	85	11	18	9		conserved protein of unknown function
Mfumv2_2446	555		*	345	211	386	106	640	803	*	Cytochrome c family protein (Modular protein)
Mfumv2_2448	906	thrB	*	73	76	13	7	143	382	*	Homoserine kinase
Mfumv2_2449	810	glpG		170	136	101	37	125	280		Membrane associated serine protease
Mfumv2_2450	885	nadC		61	66	100	29	137	316		quinolinate phosphoribosyltransferase

Mfumv2_2451	801			347	261	92	39	271	592		PATAN domain containing protein
Mfumv2_2452	564		*	594	362	421	121	1470	2684	*	Predicted GTPase
Mfumv2_2453	375			1675	691	1228	260	4482	6308		Roadblock/LC7 family protein
Mfumv2_2454	858	atpB	*	1059	965	967	398	1932	4390	*	ATP synthase subunit a
Mfumv2_2455	213	atpE	*	871	207	673	50	2366	1118	*	ATP synthase subunit c 2
Mfumv2_2456	531	atpF	*	2393	1405	1739	449	4087	5959	*	ATP synthase subunit b
Mfumv2_2457	396	atpH	*	427	196	333	66	1231	1244	*	F0F1-type ATP synthase, delta subunit
Mfumv2_2458	1554	atpA	*	1023	1718	959	781	2444	9889	*	F1 sector of membrane-bound ATP synthase, alpha subunit
Mfumv2_2459	882	atpG	*	602	637	441	225	1354	2660	*	ATP synthase gamma chain
Mfumv2_2460	1404	atpD	*	732	1265	1203	780	2177	8193	*	membrane-bound ATP synthase , F1 sector, beta-subunit
Mfumv2_2461	438	atpC	*	803	451	943	170	1376	1527	*	ATP synthase epsilon chain
Mfumv2_2462	306			425	123	209	24	564	465		conserved protein of unknown function
Mfumv2_2463	936	mesJ	*	261	251	148	52	517	1001	*	tRNA(Ile)-lysidine synthase
Mfumv2_2464	753	rsuA		95	73	91	27	125	234		Pseudouridine synthase
Mfumv2_2465	1395	argH		78	132	68	51	203	818		Argininosuccinate lyase
Mfumv2_2466	1686	ccmC	*	245	365	58	63	347	1654	*	ABC-type transport system involved in cytochrome c biogenesis, permease component
Mfumv2_2467	162			80	9	0	0	51	30		conserved protein of unknown function
Mfumv2_2468	1248	ribBA		242	330	120	54	785	2908		Riboflavin biosynthesis protein RibBA [Includes: 3,4-dihydroxy-2-butanone 4-phosphate synthase ; GTP cyclohydrolase-2]
Mfumv2_2469	765			128	114	76	23	303	706		conserved protein of unknown function
Mfumv2_2470	609			188	121	187	38	290	434		conserved protein of unknown function
Mfumv2_2471	972	haoB		179	222	163	66	302	771		Hydroxyalamine oxidoreductase associated protein
Mfumv2_2472	2148	haoA		402	917	109	116	351	2066		Hydroxylamine oxidoreductase
Mfumv2_2474	231			14	3	25	2	16	5		conserved protein of unknown function
Mfumv2_2475	210			6	3	34	3	30	9		conserved protein of unknown function
Mfumv2_2476	504			82	45	56	12	144	237		conserved protein of unknown function
Mfumv2_2477	300			43	13	105	7	27	17		conserved protein of unknown function
Mfumv2_2478	198			0	0	0	0	8	2		conserved protein of unknown function
Mfumv2_2479	678			159	104	60	26	243	503		conserved protein of unknown function
Mfumv2_2480	516			108	59	39	13	247	443		Isopentenyl-diphosphate delta-isomerase
Mfumv2_2481	627			79	50	39	13	120	220		Predicted membrane protein
Mfumv2_2482	1170			100	147	91	46	272	943		Predicted secreted hydrolase
Mfumv2_2483	2553	salY		113	299	125	113	145	1085		ABC-type antimicrobial peptide transport system,permease component
Mfumv2_2484	678	lolD		321	248	507	123	474	861		Lipoprotein-releasing system ATP-binding protein LolD
Mfumv2_2485	1350			205	299	263	135	255	965		Stand-alone CHASE2 sensor domain
Mfumv2_2486	945			86	87	69	21	164	407		conserved protein of unknown function
Mfumv2_2487	735			176	117	109	38	115	215		Biopolymer transport protein (Modular protein)
Mfumv2_2488	429	exbD		137	60	89	14	66	73		Biopolymer transport protein
Mfumv2_2489	798			78	63	69	21	128	324		putative Periplasmic protein TonB
Mfumv2_2490	228			62	13	37	4	57	37		conserved protein of unknown function
Mfumv2_2491	1146	tolB		118	145	55	33	131	420		Periplasmic component of the Tol biopolymer transport system
Mfumv2_2492	540	ompA		128	82	104	31	228	322		Outer membrane protein or related peptidoglycan-associated (Lipo)protein
Mfumv2_2493	1371			217	316	163	99	258	1049		conserved protein of unknown function
Mfumv2_2494	1032			54	59	50	16	114	372		conserved membrane protein of unknown function
Mfumv2_2495	786			166	141	124	37	189	441		conserved exported protein of unknown function
Mfumv2_2496	2613	nrfG		79	263	102	102	130	920		TPR repeats containing protein
Mfumv2_2497	1167			269	397	248	108	280	648		conserved protein of unknown function
Mfumv2_2498	309	nirD		2602	800	1308	247	1991	2337		Ferredoxin
Mfumv2_2499	1038	erfK		512	608	414	200	743	2475		Uncharacterized erfK/srfK family protein
Mfumv2_2500	759	sufC		687	457	487	127	716	1177		component of SufBCD complex, ATP-binding component of ABC superfamily
Mfumv2_2501	1425	sufB		994	1473	579	402	880	3831		component of SufBCD complex
Mfumv2_2502	1293	sufB		321	483	268	149	637	2273		Cysteine desulfurase activator SufB
Mfumv2_2503	555	paaD		2738	1480	1668	458	1383	2210		Predicted metal-sulfur cluster biosynthetic enzyme
Mfumv2_2504	1287			210	305	209	124	260	991		conserved protein of unknown function
Mfumv2_2505	579			456	285	278	83	804	1805		conserved exported protein of unknown function
Mfumv2_2506	1098			71	90	43	24	139	471		Predicted nucleoside-diphosphate-sugar epimerase
Mfumv2_2507	1137	iscS		85	113	106	43	177	540		Cysteine desulfurase

Mfumv2_2508	873	srpH	*	171	180		203	59		252	642	* Serine acetyltransferase, plasmid
Mfumv2_2509	888	aroE	*	268	232		240	88		291	757	* Shikimate dehydrogenase
